# Amplified seasonality in western Europe in a warmer world

**DOI:** 10.1126/sciadv.adl6717

**Published:** 2024-05-15

**Authors:** Niels J. de Winter, Julia Tindall, Andrew L. A. Johnson, Barbara Goudsmit-Harzevoort, Nina Wichern, Pim Kaskes, Philippe Claeys, Fynn Huygen, Sonja van Leeuwen, Brett Metcalfe, Pepijn Bakker, Stijn Goolaerts, Frank Wesselingh, Martin Ziegler

**Affiliations:** ^1^ Department of Earth Sciences, Vrije Universiteit Amsterdam, Amsterdam, Netherlands.; ^2^ Archaeology, Environmental Changes and Geochemistry group, Vrije Universiteit Brussel, Brussels, Belgium.; ^3^ School of Earth and Environment, University of Leeds, Leeds, UK.; ^4^ School of Science, University of Derby, Derby, UK.; ^5^ Department of Estuarine and Delta Systems, Royal Netherlands Institute for Sea Research, Texel, Netherlands.; ^6^ Department of Earth Sciences, Utrecht University, Utrecht, Netherlands.; ^7^ Institut für Geologie und Paläontologie, Universität Münster, Münster, Germany.; ^8^ Laboratoire G-Time, Université Libre de Bruxelles, Brussels, Belgium.; ^9^ Institut für Geowissenschaften, Universität Kiel, Kiel, Germany.; ^10^ Laboratory of Systems and Synthetic Biology, Wageningen University & Research, Wageningen, Netherlands.; ^11^ Directorate Earth and History of Life, Royal Belgian Institute for Natural Sciences, Brussels, Belgium.; ^12^ Naturalis Biodiversity Center, Leiden, Netherlands.

## Abstract

Documenting the seasonal temperature cycle constitutes an essential step toward mitigating risks associated with extreme weather events in a future warmer world. The mid-Piacenzian Warm Period (mPWP), 3.3 to 3.0 million years ago, featured global temperatures approximately 3°C above preindustrial levels. It represents an ideal period for directed paleoclimate reconstructions equivalent to model projections for 2100 under moderate Shared Socioeconomic Pathway SSP2-4.5. Here, seasonal clumped isotope analyses of fossil mollusk shells from the North Sea are presented to test Pliocene Model Intercomparison Project 2 outcomes. Joint data and model evidence reveals enhanced summer warming (+4.3° ± 1.0°C) compared to winter (+2.5° ± 1.5°C) during the mPWP, equivalent to SSP2-4.5 outcomes for future climate. We show that Arctic amplification of global warming weakens mid-latitude summer circulation while intensifying seasonal contrast in temperature and precipitation, leading to an increased risk of summer heat waves and other extreme weather events in Europe’s future.

## Teaser

Seasonal-scale climate reconstructions and models show that CO_2_ increase warms up Europe’s summers faster than its winters.

## INTRODUCTION

The mid-Piacenzian Warm Period (mPWP) represents an interval with global mean surface temperature estimated on average 3.2°C (1.7° to 5.2°C) higher than in preindustrial modern times due predominantly to elevated atmospheric CO_2_ concentrations [~400 parts per million by volume (ppmV)] (*1*–*3*). Temperatures during this period closely resemble the global mean surface temperature projected for the year 2100 following the Shared Socioeconomic Pathway (SSP) 2-4.5 (*4*–*7*), the SSP scenario most likely to be realized given current global climate policy (*8*). The Pliocene Model Intercomparison Project 2 (PlioMIP2) consortium has targeted the mPWP using an ensemble of General Circulation Models (*9*). This allows a detailed data-model comparison, which is essential for assessing the performance of climate models simulating climate scenarios with higher temperatures than represented in instrumental records, similar to conditions that will most likely define future climate on Earth (*2*, *5*).

One region under extreme threat in a global warming scenario is the Arctic region, which is anticipated to warm up two to four times faster than the global average, a phenomenon called Arctic amplification (*10*). Model experiments using future CO_2_ forcing scenarios imply that Arctic amplification caused by global warming of mPWP magnitude might affect the storm track–affected regions of Europe by weakening atmospheric summer circulation (*11*), significantly reducing summer cloud cover (*12*) and leading to persistently elevated summer temperatures and droughts (*13*, *14*). Taking into account these “dynamic” drivers of summer weather extremes, related to changes in atmospheric circulation, more extreme weather variability would be projected for the future than the variability expected from “thermodynamic” drivers of temperature changes alone (*14*). Given this season-specific response of weather patterns to warming, seasonal-scale reconstructions of mPWP climate are crucial to validate climate model simulations and improve our understanding of the impact of higher atmospheric greenhouse gas concentrations on European climate.

Yet, climate proxy records from pelagic sediment cores, which form the predominant means of estimating mPWP temperatures (*15*–*17*), rarely resolve seasonal-scale variability in the temperature cycle due to low temporal resolution. Furthermore, short-lived marine organisms that make up pelagic sediments preferentially grow only during specific seasons with favorable conditions. This seasonal bias places considerable uncertainty on marine climate reconstructions and data-model comparisons based on common temperature proxies, such as Mg/Ca and stable isotope ratios in foraminifera (*18*), TEX_86_ from membrane lipids in Crenarchaeota (*19*), and U_37_^K′^ based on ketones in haptophyte algae (*20*). These uncertainties probably account for the difficulty of reconciling seasonal-scale mPWP climate reconstructions and models of terrestrial seasonality (*21*).

One approach to facilitate seasonal data-model comparison is to use information from marine mollusks, which record daily- to decadal-scale variability in the chemistry of their calcium carbonate shells (*22*–*25*) and preserve this archive of high-resolution environmental variability over geological timescales (*26*–*28*). Climate reconstructions at the seasonal scale from mid-latitude shelf seas, where appropriate mollusks are common, are instrumental in assessing the impact of warming on the seasonal scale because these regions experience high seasonal temperature contrast and their ecosystems are strongly affected by climate change (*29*–*32*). Going a step further, by using the clumped isotope composition of shell carbonate, it becomes possible to reconstruct seasonal temperature changes of the water in which mollusks grow without relying on uncertain assumptions of the isotopic composition of past ocean water, leading to accurate reconstructions of past seasonality (*26*, *33*, *34*).

**Fig. 1.**
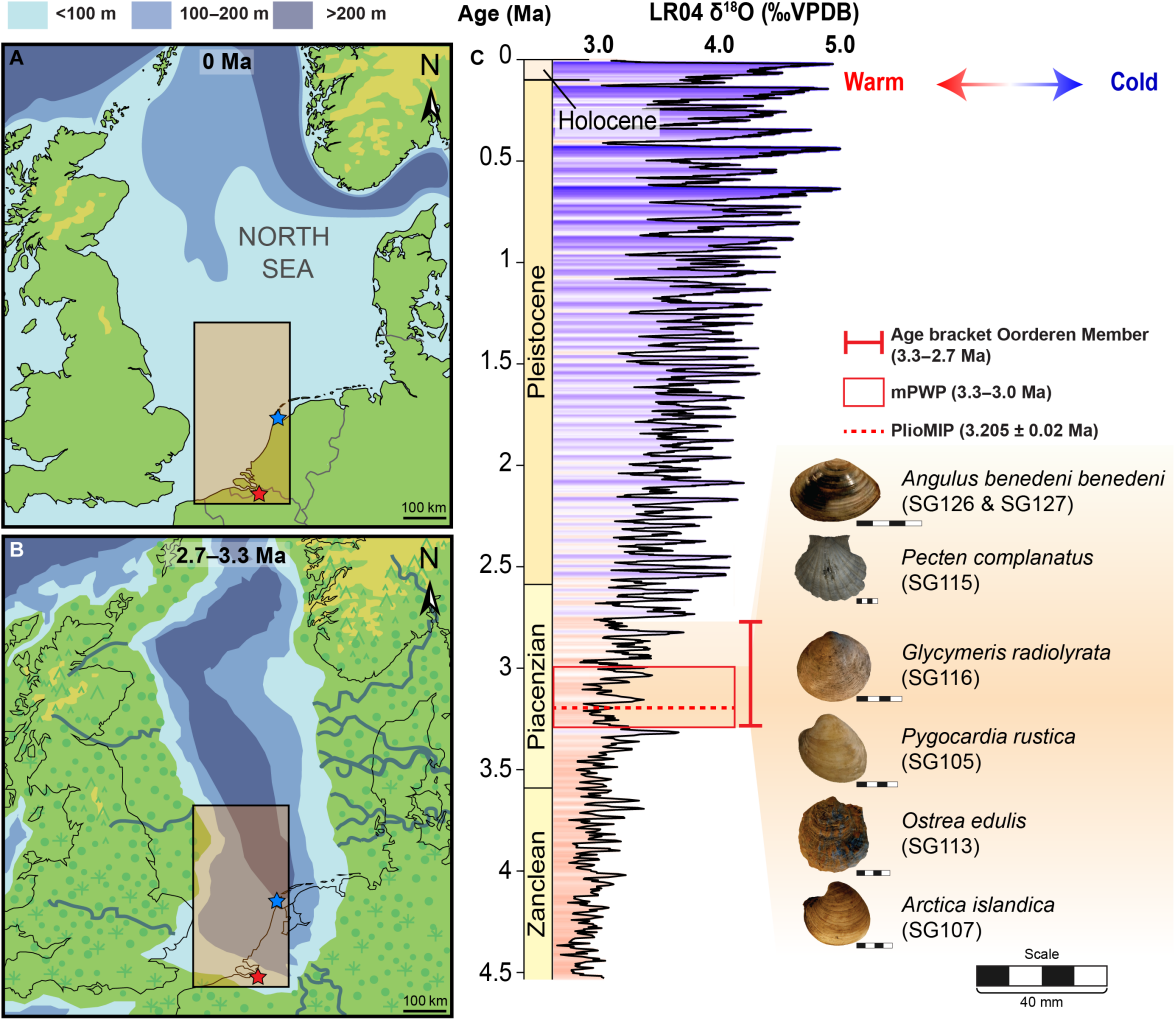
Locality of the study site and long-term climate context. Maps of northwestern Europe showing the present (**A**) and estimated mPWP (**B**) [after (*35*, *37*, *67*)] SNS bathymetry. The locality of fossil shells (red star), the local modern temperature record (blue star), and the area containing the SNS data integrated from extended reconstructed SST (Extended Reconstructed SST v5), PlioMIP2, and CMIP6 models (black rectangle with orange filling; 51°N to 55°N, 2°E to 4°E; Figs. 2 and 3) are indicated. (**C**) Age bracket of the Oorderen Member (Lillo Formation) containing the mollusk specimens (*35*) (orange shaded region) relative to the mPWP (*82*) (red box) and PlioMIP2 (*1*) interval (red dashed line) and the global benthic foraminifera oxygen isotope stack (*83*) with the relative temperature change indicated. Note that mollusk specimens likely originate from highstand (i.e., warmer) periods within the age bracket (see Materials and Methods).

**Fig. 2.**
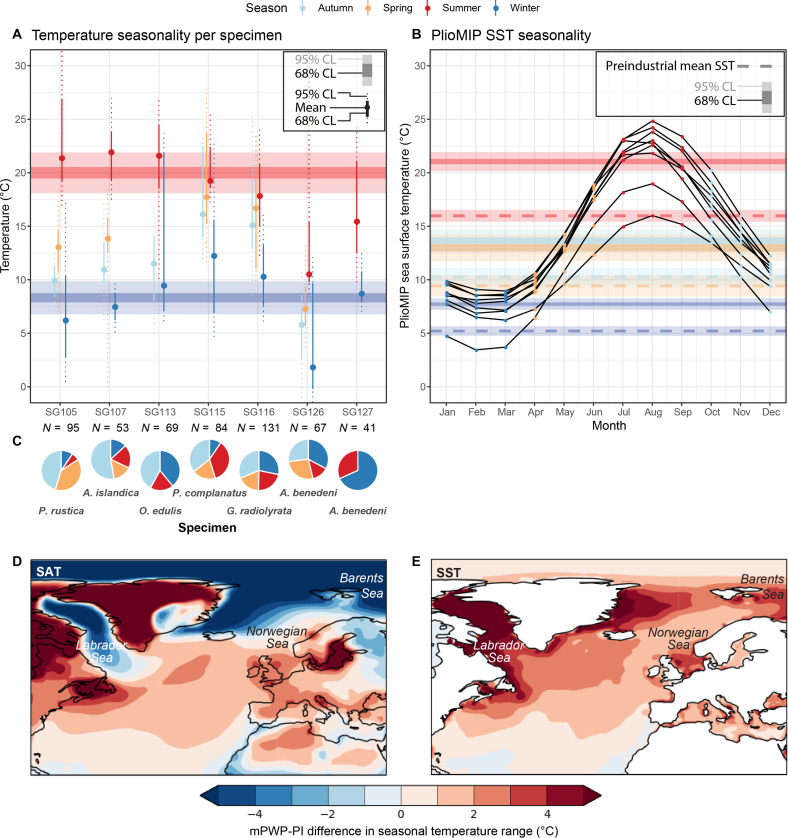
Data-model comparison. (**A**) Weighted mean seasonal temperatures per specimen (filled circles with solid and vertical dotted lines indicating 68 and 95% confidence intervals, respectively) and summer and winter temperatures based on the complete dataset (horizontal shaded bars in red and blue, respectively) with propagated uncertainties [68 and 95% confidence level (CL); see Materials and Methods]. Horizontal dashed lines in the same colors indicate mean preindustrial SNS SST. (**B**) Local monthly SST outcomes of individual PlioMIP2 models with winter (January to March; blue) and summer (July to September; red) temperature estimates (horizontal shaded bars showing 68 and 95% CL). Dashed lines in the same colors indicate mean SST outcomes [with uncertainty; 95% CL (*9*)] from preindustrial control simulations using the same models. (**C**) Clumped isotope sample sizes and seasonal representation of samples per specimen. (**D** and **E**) Spatial variability in the difference in seasonal contrast (July to January) in SAT(D) and SST (E), respectively, between PlioMIP2 multi-model mean mPWP runs and PlioMIP2 preindustrial (PI) control runs of the same models.

**Fig. 3.**
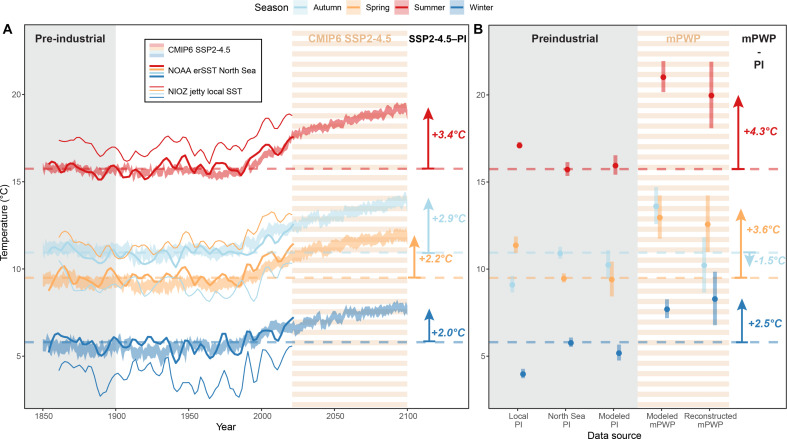
Pliocene reconstructions inform future climate projections. (**A**) Seasonal local (NIOZ jetty, Texel, The Netherlands) SST measurements (thin solid lines; LOESS-smoothed with span of 10% of the record), National Oceanic and Atmospheric Administration Extended Reconstructed SST v5 (NOAA ERSSTv5) averages for the SNS (thick solid lines; LOESS-smoothed with span of 10% of the record), and CMIP6 SSP2-4.5 scenario transient SNS SST simulations (shaded area) between 1850 and 2100. Arrows and figures on the right indicate temperature differences between CMIP6 SSP2-4.5 projections for 2100 CE and preindustrial SNS SSTs (dashed lines). (**B**) Preindustrial (before 1900 CE) seasonal SST averages based on historical SST records [NOAA ERSSTv5 (*41*) SNS averages and NIOZ jetty local record (*42*)] and PlioMIP2 preindustrial model outcomes are compared with mPWP seasonality based on PlioMIP2 mPWP model simulations and mollusk-based reconstructions. Arrows and figures on the right indicate seasonal temperature differences between mollusk-based mPWP temperature reconstructions and preindustrial SNS SST (dashed lines). The gray background color indicates the preindustrial reference period (1850 to 1900 CE), while horizontal orange and white banding indicates the CMPI6 SSP2-4.5 model projections and mPWP model outcomes and reconstructions.

We present seasonal sea surface temperature (SST) reconstructions based on clumped isotope analyses from well-preserved fossil mollusk shells originating from the Oorderen Member of the Lillo Formation (northern Belgium; see Materials and Methods). These sediments were deposited below fair-weather wave base (30 to 50 m water depth) in the southern North Sea (SNS) under warm, high sea-level conditions during and slightly after the mPWP [2.7 to 3.3 million years ago (Ma) (*35*, *36*); Fig. 1]. The shells record marine temperatures in the mPWP SNS (*37*), an important model ecosystem for temperate shelf seas worldwide (*38*). On the basis of the observation of sedimentary structures indicative of strong tidal mixing (*39*), the absence of dysoxic fauna (see Materials and Methods), and the observation of high spring temperatures (see Results; Figs. 2 and 3), it seems likely that the shells used in this study record conservative summer temperatures close to the true summer SST during the mPWP.

We directly compare our seasonal SST reconstructions with ensemble model simulations of seasonal temperature change in the mPWP SNS, previously produced by the PlioMIP2 consortium (*40*) as well as with instrumental temperature records in the region and Coupled Model Intercomparison Project Phase 6 (CMIP6) SSP2-4.5 projections of European climate for the year 2100. This approach offers a detailed glimpse of the potential future climate in Europe under a moderate emission scenario.

## RESULTS

### Data-model agreement on enhanced summer warming in the North Sea

Aggregated results from all bivalve specimens give weighted winter (8.3° ± 1.5°C), spring (12.6° ± 1.6°C), summer (20.0° ± 1.9°C), and autumn (10.2° ± 1.6°C) SST averages during the mPWP in the SNS (Fig. 2A). The reconstructed winter and summer temperatures agree with winter (January to March; 7.7° ± 0.5°C) and summer (July to September; 21.1° ± 0.9°C) temperatures in the SNS from aggregated PlioMIP2 model results (Fig. 2B). Both reconstructed and modeled winter and summer temperatures are significantly higher than winter (5.2° ± 0.9°C) and summer (16.0° ± 0.6°C) temperatures in preindustrial (280 ppmV CO_2_) PlioMIP2 control runs and instrumental records (Fig. 2, A and B). Our reconstructions yield 2.5° ± 1.5°C warmer winters and 4.3° ± 1.9°C warmer summers during the mPWP compared to mean winter (5.8° ± 0.3°C) and summer (15.7° ± 0.4°C) temperatures in the SNS over the period 1854 to 1900 CE [Fig. 3A; based on National Oceanic and Atmospheric Administration Extended Reconstructed SST v5 (NOAA ERSSTv5) (*41*)].

### Locally elevated spring temperatures

Intriguingly, mollusk-based reconstructions yield significantly higher spring (April to June; 12.6° ± 1.6°C) than autumn (October to December; 10.2° ± 1.6°C) temperatures during the mPWP. This contrasts with regional SNS SST averages from both preindustrial (spring: 9.4° ± 1.0°C; autumn: 10.3° ± 0.8°C) and mPWP (spring: 13.0° ± 1.2°C; autumn: 13.6° ± 1.1°C) PlioMIP2 model runs as well as ERSST SNS SST averages (spring: 9.5° ± 0.3°C; autumn: 10.9° ± 0.3°C), which consistently yield higher temperatures in autumn compared to spring. Contrarily, local preindustrial instrumental SST time series from the Netherlands Institute for Sea Research (NIOZ) monitoring station on the SNS coast [island of Texel, northwestern Netherlands (*42*); see Fig. 1, A and B; Supplementary Methods] do exhibit significantly elevated spring (11.4° ± 0.5°C) compared to autumn (9.1° ± 0.5°C) temperatures. Shallow coastal waters, such as the tidal flats of the Wadden Sea close to the NIOZ monitoring station, are highly responsive to air temperature, which leads to elevated SSTs in the spring season, while more offshore areas of the North Sea have higher thermal inertia, explaining elevated autumn temperatures (*42*). The elevated spring and reduced autumn SST reconstructions thus highlight that the mollusk shells record detailed seasonal SST variability in the coastal SNS during the mPWP.

### Interspecimen variability

Reconstructed seasonal temperatures vary between fossil shell specimens, with some specimens recording warmer temperatures (e.g., *Pecten complanatus*; SG115; winter: 12.3° ± 4.4°C; summer: 19.3° ± 1.8°C) and others cooler temperatures (e.g., *Angulus benedeni benedeni*; SG126 and SG127; winter: 1.9° ± 4.9°C; summer: 15.5° ± 4.3°C; Fig. 2A). These interspecimen differences probably occur due to the occurrence of growth halts and variations in growth rates between seasons (Fig. 2C) and between consecutive growth years (see figs. S13 to S26), which dampen seasonality due to the averaging of samples around the seasonal extreme. In addition, variability between specimens may arise from differences in the seasonal temperature range within their age range (2.7 to 3.3 Ma; see Fig. 1C). The latter may explain the relatively warm spring temperatures recorded by specimens SG115 and SG116 from the *Cultellus* level and comparatively cooler temperatures recorded by SG126 and SG127 from the *benedeni* level at the top of the Oorderen Member (see table S1). Our weighted averages of summer and winter temperatures (Fig. 2A) ensure that this interspecimen variability does not bias the reconstructions.

### PlioMIP2 models reproduce SST seasonality in the modern North Sea

Transient simulations of SNS SST seasonality from before the Industrial Revolution (1850 CE) to present day (2023 CE) by the CMIP6 model ensemble (*6*) mimic historical SST records from the region (Fig. 3A) (*41*). Seasonal SNS SST simulations by the PlioMIP2 model ensemble under preindustrial radiative forcing (*9*) (winter: 5.2° ± 0.5°C; summer: 16.0° ± 0.6°C) closely resemble preindustrial (1850 to 1900 CE) CMIP6 seasonal SNS SST simulations (winter: 5.5° ± 0.3°C; summer: 15.7° ± 0.7°C) and historical winter (5.8° ± 0.3°C) and summer SNS SST (15.7° ± 0.4°C) records (Fig. 3B) (*41*). Local SST records from the NIOZ monitoring station in Texel (*42*) reveal slightly higher seasonal SST ranges (winter: 4.0° ± 0.3°C; summer: 17.1° ± 0.2°C; Fig. 3B), which are well explained by their coastal location (see above). This agreement shows that PlioMIP2 models successfully reproduce summer and winter SSTs in the SNS.

### The mid- to high-latitude seasonal temperature response to mPWP-like warming

Maps of PlioMIP2 seasonal SST outcomes show that the enhanced seasonal contrast observed in our reconstructions is present throughout the mid-latitudes to high latitudes in the North Atlantic (Fig. 2E). Enhanced SST seasonality is more pronounced in areas that are characterized by high seasonal contrast in surface air temperature (SAT) and cold winters (e.g., Labrador Sea and Barents Sea) than in regions with milder winters (e.g., Norwegian Sea). Contrarily, SAT only shows enhanced seasonal contrast in the mid-latitudes under mPWP conditions (Fig. 2D). Areas with the strongest enhanced seasonal SST contrast (e.g., Labrador Sea and Barents Sea; Fig. 2E) show a reduced seasonal SAT contrast under mPWP conditions compared to the preindustrial control runs (Fig. 2D). PlioMIP2 model simulations of sea ice extent (fig. S33) show that these regions where the SST and SAT seasonality respond differently to mPWP-scale warming are characterized by winter sea ice cover in preindustrial runs. They lose much of their winter sea ice under mPWP conditions; a 53% decrease in mean annual sea ice extent during the mPWP compared to preindustrial climate is simulated in PlioMIP2 models (*43*). Contrarily, areas with enhanced summer warming in both SST and SAT are sea ice–free year round in both mPWP and preindustrial model runs. SAT seasonality over land (both in North America and Europe; Fig. 2D) is enhanced at both mid-latitudes and high latitudes, following the SST pattern. PlioMIP2 model outcomes show that summer cloud cover is severely reduced in the mid-latitudes of the North Atlantic, where both SST and SAT seasonality is enhanced (figs. S34 and S35).

## DISCUSSION

Recent terrestrial mPWP data-model comparisons highlight that PlioMIP2 models cannot reproduce the full extent of the mPWP warming that data suggest (*2*, *21*, *44*). Likewise, in both the pelagic and terrestrial realm, reconstruction of the full seasonal cycle is challenging due to proxy limitations (*2*, *21*). Detailed temperature monitoring studies show that shallow marine temperatures are strongly linked to air temperatures and local weather systems (*45*). Together with the enhanced occurrence of summer stratification in warmer climates (*24*, *46*, *47*), a change in atmospheric circulation patterns may drive the summer increase in SST during the mPWP and in the future observed in data and models (Figs. 2 and 3). The seasonal cycle is much more pronounced in the terrestrial realm, which could explain part of the large seasonal data-model mismatch at terrestrial Northern Hemisphere high latitudes (*21*).

PlioMIP2 models closely agree with mollusk-based reconstructions of SNS summer and winter SST during the mPWP (Figs. 2 and 3B). The summer warming observed in SST data and model outcomes is also reflected in PlioMIP2 SAT seasonality in the SNS region (Fig. 2D and fig. S37). This highlights that PlioMIP2 models can reconstruct seasonal SST variability in the North Atlantic despite the difficulties with terrestrial data-model comparisons (*21*). However, large intermodel variability in regions with poor data-model agreement suggests that structural model uncertainties also contribute to the mismatch (*21*).

The agreement between PlioMIP2 preindustrial control simulations and present-day climate (represented in CMIP6 models and instrumental records; Fig. 3) (*1*, *9*, *48*) lends confidence to the application of mPWP reconstructions and model simulations to better understand the seasonal impact of moderate global warming on future European climate. The seasonal-scale data-model agreement in the SNS, situated between the pelagic and terrestrial realms, for both present-day and mPWP climate, sheds light on the seasonal response to warmer climate in the mid-latitudes: The unique potential of marine mollusks to record seasonal-scale SST variability in coastal regions (*28*, *49*) in combination with the strong relationship between mid-latitude coastal SST and SAT (*45*, *50*) (see Fig. 2D and figs. S28 and S32) highlights the key role of seasonally resolved shallow marine climate archives for better understanding past and future climates.

Our mollusk-based data and PlioMIP2 model estimates of mPWP SST seasonality resemble projections of future (2081 to 2100 CE) SNS winter (7.7° ± 0.3°C) and summer SST (19.1° ± 0.5°C; Fig. 3A) by transient CMIP6 model simulations of the “middle-of-the-road” SSP2-4.5 scenario (Fig. 3) (*48*). Note that these are two different climate scenarios: CMIP6 represents a transient climate disturbance, while the mPWP simulations and reconstructions sample an equilibrated climate under long-term atmospheric CO_2_ forcing of 400 ppmV (*9*). The difference is evident from atmospheric *P*co_2_ (partial pressure of CO_2_) projections of ~600 ppmV in 2100 CE and ~580 ppmV in the long term (2500 CE) under SSP2-4.5, which notably exceed the mPWP estimate of ~400 ppmV (*3*, *5*). Notwithstanding this difference, the results of our data-model comparison and the similarity of the SSP2-4.5 scenario to the current global emission trajectory (*4*) show that the mPWP presents valuable lessons for climate in Europe by the end of this century and models are able to project seasonal climate in this region in a warmer world.

The spatial variability in seasonal response to mPWP warming of SST and SAT reveals a clear signature of sea ice extent: SST and SAT respond in opposite directions to mPWP warming in the higher-latitude areas that lose winter sea ice cover under mPWP conditions (Fig. 2, D and E; e.g., Labrador Sea and Barents Sea; fig. S33). The loss of winter sea ice in an mPWP-like climate scenario facilitates heat transfer from the upper ocean to the lower atmosphere in winter, cooling the upper ocean in winter (enhancing SST seasonality) and warming the lower atmosphere [reducing SAT seasonality; as demonstrated in (*43*)]. This effect explains why higher latitudes are more affected by the sea ice–albedo feedback, which changes due to a reduction in sea ice extent under mPWP-like conditions (*51*, *52*) and in future climate scenarios (*53*). In these regions, Arctic amplification is expected to cause winter SAT to warm faster than other regions (*10*, *54*), while SST actually shows a summer warming effect in both the high latitudes and mid-latitudes.

This hypothesis is supported by climate models showing that Arctic amplification reduces the latitudinal SAT gradient in warmer climates (*10*). This mechanism has been demonstrated to weaken the mid-latitude atmospheric summer circulation in the present-day climate, causing a weakening of storm tracks, with lower cyclone activity over Europe, more persistent weather conditions, and a reduction in summer cloud cover (*11*). This reduction in cloud cover over temperate latitudes of the North Atlantic is also simulated in PlioMIP2 model outcomes (fig. S35) (*13*), thus suggesting that a similar mechanism causes the seasonal temperature structure in the mPWP. Recent observations from various parts of the North Atlantic region suggest that circulation changes are already enhancing summer warming and prolonging heat waves through low cyclone activity, low cloud cover, and more persistent weather conditions (*12*, *55*, *56*). In the winter months, zonal (westerly) wind increases, enhancing cloud cover and precipitation and suppressing winter warming (*13*). Together, these changes in the atmospheric circulation regime result in an increase in extreme weather conditions in Europe in both seasons, with more severe precipitation events in winter and more prolonged droughts in summer (*13*).

Our data-model analysis reveals that an mPWP-scale SST increase in the SNS region causes more pronounced warming in summer (reconstructions: +4.3° ± 1.9°C; PlioMIP2 models: +5.1° ± 0.9°C) than in winter (reconstructions: +2.5° ± 1.5°C; PlioMIP2 models: +2.5° ± 0.5°C; Fig. 3B). Historical climate data (Fig. 3A) and recent climate attribution studies (*57*–*59*) reveal that this trend is already underway in Europe (*60*). Therefore, reconstructions and model simulations of mPWP seasonality reveal the impact of our current climate trajectory: Because of the hypothesized mid-latitude circulation changes, global warming is likely to regionally shift the distribution of extreme weather events on top of the overall increase in frequency and severity of these events due to the thermodynamic effect of greenhouse warming (*14*). The seasonally asymmetric change in mid-latitude zonal circulation, even under a relatively mild mPWP-like warming scenario, renders Europe particularly vulnerable to prolonged heat and drought in summer and intense precipitation events in winter (*13*). Enhanced summer warming in coastal regions poses a serious risk for shallow marine communities since it leads to more frequent and extreme weather events, such as marine heat waves (*29*, *30*, *57*, *59*, *61*). These marine heat waves (*62*) have recently caused mass mortality events (*63*), mass coral bleaching (*64*), and toxic algal blooms (*65*), severely affecting marine biodiversity. They are among the major reasons for concern related to anthropogenic climate change (*66*). Considering the high societal and ecological importance of mid-latitude coastal regions, local data-model comparison studies of short-term variability during past climates relevant for future warming scenarios play a fundamental role in honing the model projections that inform climate adaptation strategies.

## MATERIALS AND METHODS

### Specimen collection

Seven fossil mollusk specimens were collected by one of the authors (S.G.) for analysis from temporary exposures of the Oorderen Member of the Lillo Formation in the Antwerp area (Belgium; see Fig. 1). These were specimens of *A. benedeni benedeni* (specimen ID: SG126 and SG127) from Deurganckdoksluis (51°16′49″N, 4°14′56″E; collected in 2013), *P. complanatus* (SG115) and *Glycymeris radiolyrata* (SG116) from Deurganckdok (51°17′24″N, 4°15′37″E; collected on 2 February 2001), and *Pygocardia rustica* (SG105), *Ostrea edulis* (SG113), and *Arctica islandica* (SG107) from Verrebroekdok (51°16′16″N, 4°12′53″E; collected in 1999–2000). At these localities, the Oorderen Member, from bottom to top, is divided into basal shell bed, *Atrina* level, *Cultellus* level (SG115 and SG116), and *benedeni* level (SG126 and SG127) (*37*). Specimens SG105, SG113, and SG107 were collected ex situ within the Oorderen Member. The estimated age range for the Oorderen Member is 2.72 to 3.3 Ma (see Fig. 1) (*35*, *67*).

### Paleoenvironmental context

Field observations and a detailed assessment of the composition of the invertebrate fauna reveal that the fossil-bearing sediments of the Oorderen Member were deposited between 30 and 50 m water depth during warm, highstand intervals (*35*, *36*). Previous studies have argued that the SNS undergoes summer stratification during the mPWP, which would cause molluscan shell reconstructions to underestimate summer temperatures (*24*, *47*). However, the lack of dysoxic faunas in the Oorderen Member suggests the absence of persistent stratification (*35*). Furthermore, the presence of sedimentary structures indicative of tidal currents and strong vertical mixing during deposition of the Oorderen Member argues against a large surface to seafloor temperature gradient (*39*). Therefore, it is likely that the mollusks recorded temperatures close to the SSTs year round. This assessment is supported by the evidence of warm spring and cool autumn temperatures, suggesting a direct response of the water temperature experienced by the mollusks to the seasonal cycle in SATs (see Results; Fig. 3). However, we cannot fully exclude that spatially and temporally restricted temperature stratification did occur during the lifetime of the studied specimens (*24*, *47*). Note that, even if summer stratification occurred, the summer temperatures recorded by the mollusks would underestimate the true mPWP summer SST and the occurrence of enhanced summer warming during the mPWP would still be supported by the data.

### Specimen preparation

Shells were partially embedded in epoxy resin before polished thick sections were prepared to expose a cross section through the axis of maximum shell growth [following (*68*); see fig. S3]. Preservation of the original shell calcite and aragonite was verified using a combination of scanning electron microscopy (SEM), cathodoluminescence microscopy, electron backscatter diffraction microscopy (EBSD), micro–x-ray fluorescence (μXRF), and x-ray diffraction [XRD; see (*67*) and figs. S4 to S12]. Only shells with excellent preservation as demonstrated by these methods were considered for clumped isotope analysis.

### Geochemical analysis

Carbonate was sampled along transects in the direction of growth in transects on cross sections through the outer shell layers of all shells except *P. complanatus* (SG115) and *A. benedeni benedeni* (SG126 and SG127), whose thin outer shell layers necessitated sampling on the outside of the shell. A combination of handheld drilling (outside) and micromilling (in transects) was used to sample along narrow growth increments to minimize time averaging (Supplementary Materials). The number of replicates per specimen ranges from 61 to 157 (Fig. 2): SG105, *n* = 98; SG107, *n* = 63; SG113, *n* = 90; SG115, *n* = 100; SG116, *n* = 157; SG126, *n* = 79; SG127, *n* = 61. Some samples were replicated more than once, causing multiple replicates to originate from the same location in the shell, and some of these replicate measurements were discarded as outliers during data processing (see below). Measurements from *A. benedeni benedeni* (SG126 and SG127) include data from (*67*) as well as additional analyses on the same specimens carried out for this study. Small (70 to 160 μg) aliquots of each sample were digested in phosphoric acid for 600 s at 70°C in a Kiel IV carbonate preparation device, after which the resultant CO_2_ gas was purified using two cold fingers and a Porapak trap (*69*) before the clumped isotope composition (Δ_47_) was analyzed using two Thermo MAT253 mass spectrometers. A long-integration dual-inlet (LIDI) workflow (*69*, *70*) was used for all except 53 aliquots from *P. rustica* (SG105); the latter aliquots were measured using sample-standard measurement cycles [“click-clack” mode; e.g., (*71*)]. Clumped isotopic ratios were corrected for intensity-based Δ_47_ offsets based on intensity-matched ETH-3 standards (see section S3.1) before being standardized using the ETH standards (*72*), which were run in approximately 1:1 ratio with the samples (*73*) and corrected for ^17^O concentration following (*74*). Long-term analytical precision was monitored using IAEA-C2 and Merck reference materials (typical standard deviation of 0.04‰; see table S2) after outlier removal based on metadata on instrument performance (see criteria in section S3.2).

### Seasonality reconstructions

For each mollusk specimen, samples were internally dated relative to the seasonal cycle using ShellChron (*75*), after which measurements within a specimen were grouped in four 3-monthly bins (“seasons”). Summer was defined as the consecutive 3-month period with the lowest mean Δ_47_ value, calculated separately for each specimen (see section S3.2 and figs. S20 to S32). The fact that these 3-monthly bins do not always line up with the minima and maxima in the stable oxygen isotope profiles through the shells shows that the common assumption that these extreme stable oxygen isotope values represent summer and winter seasons does not always hold true [see (*76*)]. Mean seasonal Δ_47_ values were obtained through weighted averages of seasonal means and uncertainties considering uncertainty within and variability between specimens. Seasonal temperatures during the mPWP were reconstructed from Δ_47_ for each seasonal group using the clumped isotope calibration by (*77*), propagating uncertainty on Δ_47_ values following the procedure described in the supplement of (*78*).

### Climate model output

Seasonal SST from the SNS was extracted from the corresponding local ocean grid cells (51°N to 55°N, 2°E to 4°E) in the PlioMIP2 model ensemble (see Fig. 2). SNS SST records were obtained from the same area within the ERSSTv5 product (*41*) and supplemented with local SST observations from the NIOZ monitoring station (*42*). Future projections matching the SSP2-4.5 scenario (*48*) were produced for CMIP6, and the regional SST outcomes were exported from the Intergovernmental Panel on Climate Change (IPCC) WG1 Interactive Atlas (*79*). Seasons were defined as weighted means of the 3 months containing the largest number of days in the season according to the astronomical definition: winter: January to March; spring: April to June; summer: July to September; autumn: October to December. Data processing was carried out using the open-source computational software R (*80*), and scripts are provided in (*81*).

## Supplementary Materials

### This PDF file includes:

Supplementary Text Figs. S1 to S46 Tables S1 to S5 References
