# Supplementary material for: Amplified seasonality in western Europe in a warmer world
Source: Sci Adv. 2024 May 15;10(20):eadl6717. doi: 10.1126/sciadv.adl6717 (PMC11095466; doi:10.1126/sciadv.adl6717)
Supplement: Supplementary file 1 — Supplementary Text Figs. S1 to S46 Tables S1 to S5 References [file sciadv.adl6717_sm.pdf]

Science Advances
AAAS

Supplementary Materials for

Amplified seasonality in western Europe in a warmer world

Niels J. de Winter *et al.*

Corresponding author: Niels J. de Winter, n.j.de.winter@vu.nl

Sci. Adv. **10**, eadl6717 (2024)
DOI: 10.1126/sciadv.adl6717

This PDF file includes:

Supplementary Text
Figs. S1 to S46
Tables S1 to S5
References

Supplementary Information

1. Samples

Table 1 contains an overview of specimen information.

Specimen ID	Genus and Species	Collection site	Collection latitude	Collection Longitude	Collection year	Stratigraphic level	Size (mm)
SG105	*Pygocardia rustica*	Verrebroekdok, Antwerp (BE)	51°16’16”N	4°12’53”E	1999 - 2000	Ex situ Oorderen Member	69
SG107	*Arctica islandica*	Verrebroekdok, Antwerp (BE)	51°16’16”N	4°12’53”E	1999 - 2000	Ex situ Oorderen Member	67
SG113	*Ostrea edulis*	Verrebroekdok, Antwerp (BE)	51°16’16”N	4°12’53”E	1999 - 2000	Ex situ Oorderen Member	92
SG115	*Pecten complanatus*	Deurganckdok, Antwerp (BE)	51°17'24"N	4°15'37"E	Feb 2nd, 2001	*Cultellus* level, Oorderen Member	111
SG116	*Glycymeris radiolyrata*	Deurganckdok, Antwerp (BE)	51°17'24"N	4°15'37"E	Feb 2nd, 2001	*Cultellus* level, Oorderen Member	56
SG126	*Angulus benedeni benedeni*	Deurganckdoksluis, Antwerp (BE)	51°16’49”N	4°14’56”E	2013	*Benedeni* level, Oorderen Member	31
SG127	*Angulus benedeni benedeni*	Deurganckdoksluis, Antwerp (BE)	51°16’49”N	4°14’56”E	2013	*Benedeni* level, Oorderen Member	37

Table S1: Specimen and sampling information.

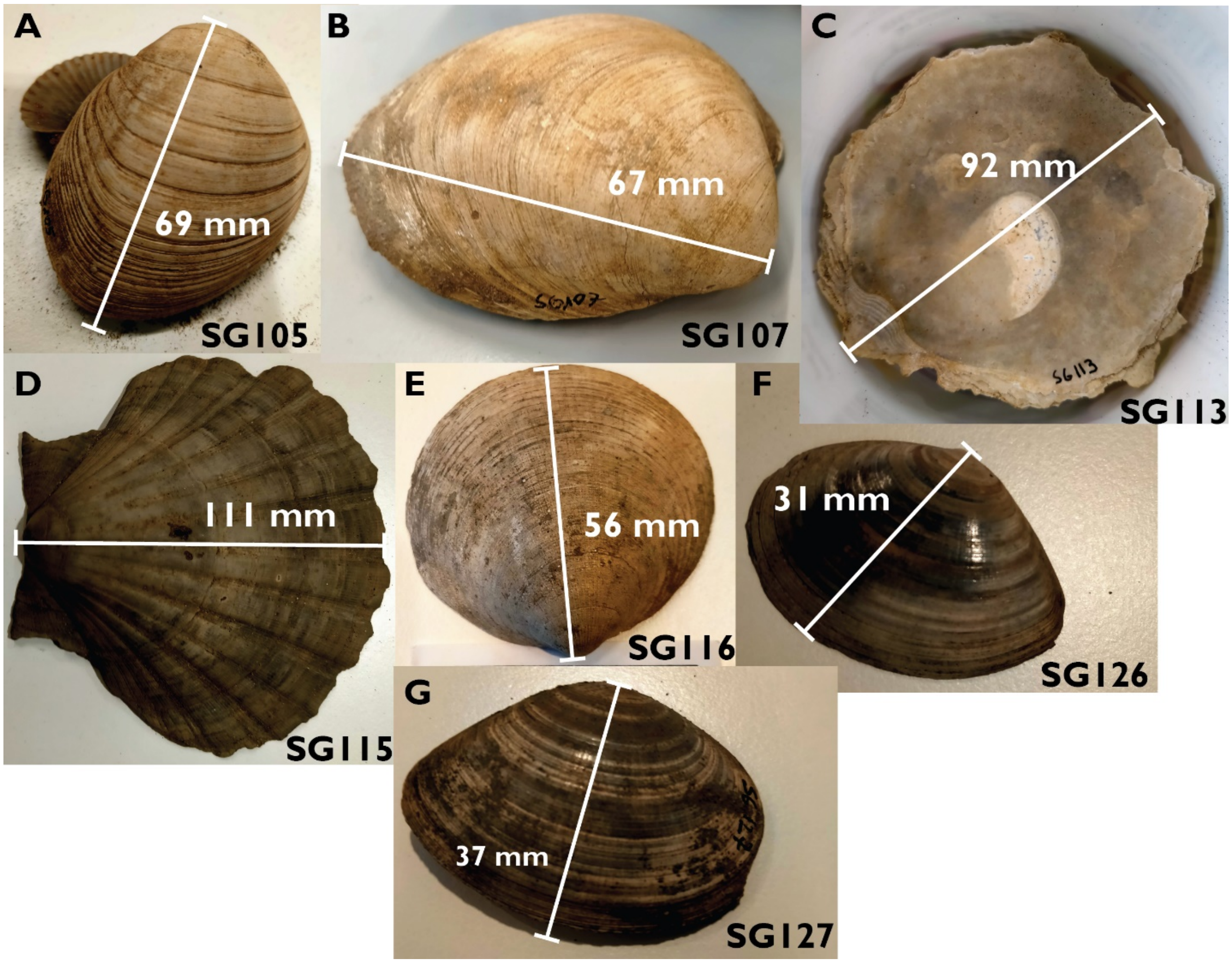

Fig S1: Overview pictures of all specimens prior to sample preparation. A) *Pygocardia rustica* specimen SG105. **B)** *Arctica islandica* specimen SG107. **C)** *Ostrea edulis* specimen SG113 (inside view). **D)** *Pecten complanatus* specimen SG115. **E)** *Glycymeris radiolyrata* specimen SG116. **F)** *Angulus benedeni benedeni* specimen SG126. **G)** *Angulus benedeni benedeni* specimen SG127. Shell heights of all specimens are indicated with white bars and are measured along the axis of maximum growth from the umbo to the ventral margin.

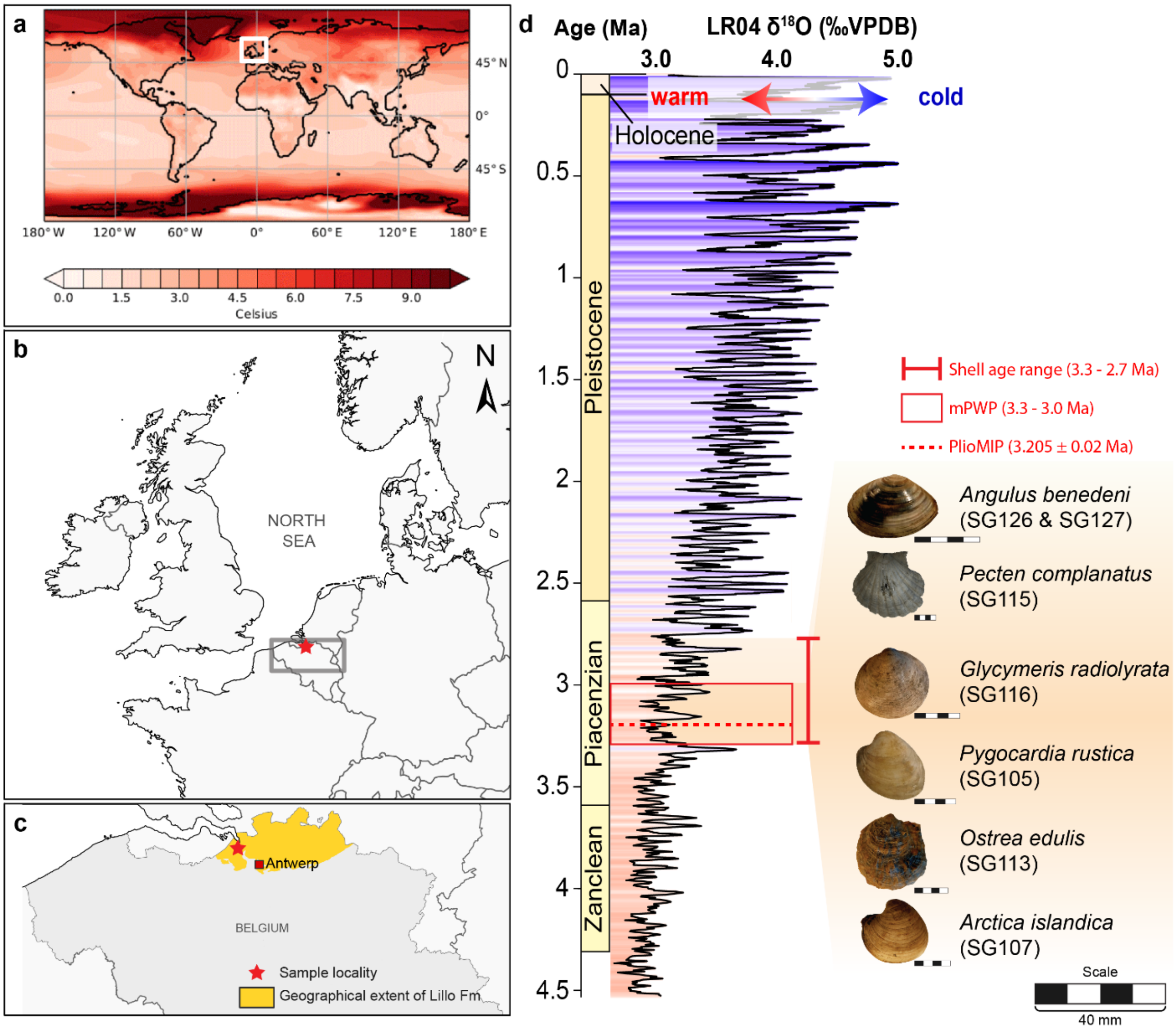

Fig S2: Location of the Oorderen Member within the Lillo Formation and global climate context. (**a**) PlioMIP2 multi-model mean surface air temperature anomaly of mPWP relative to PI control model experiments(*9*). The white box indicates the location of the map in (**b**). Maps of northwestern Europe (**b**) and northern Belgium (**c**) indicating the locality of fossil shells and the spatial extent within Belgium of the Lillo Formation (after(*35*, *37*, *67*)). **(d)** Age bracket of the Oorderen Member (red box) in the Antwerp area containing the mollusc specimens relative to the global benthic foraminifera oxygen isotope stack(*83*) with relative temperature change indicated on the right.

1.1 Sample preparation

The Oorderen Member is loosely consolidated; fossil shells thus only required minimal cleaning with a soft brush to remove remaining parts of the sediment. Prior to further treatment, small fragments were carefully broken off from a selection of aragonitic specimens (*Arctica islandica*, *Pygocardia rustica* and *Angulus benedeni benedeni*) to check for preservation of the aragonite (see below).

All specimens were then (partially) embedded in epoxy resin (Araldite 2020, Huntsman Corp., The Woodlands, TX, USA). In a first step, the concave inner side of the shell valve was filled with resin and left to harden overnight. In a second step, the specimen was placed onto a flat block of hardened epoxy and glued to it by submerging the specimen in more resin, while leaving the upper outer surface of the shell exposed (see **Fig. S3**). This procedure helped support the shells during sawing while leaving the outer shell surface exposed, allowing sampling on the outer surface when needed. Outside sampling was done for the two *A. benedeni benedeni* specimens (SG126 and SG127; see (*67*)) and for the *Pecten complanatus* shell (SG115). After these embedding steps, all shells were cut longitudinally through their axis of maximum growth (following (*68*)) to expose a cross section through all growth layers that allows precise sampling and counting of growth increments.

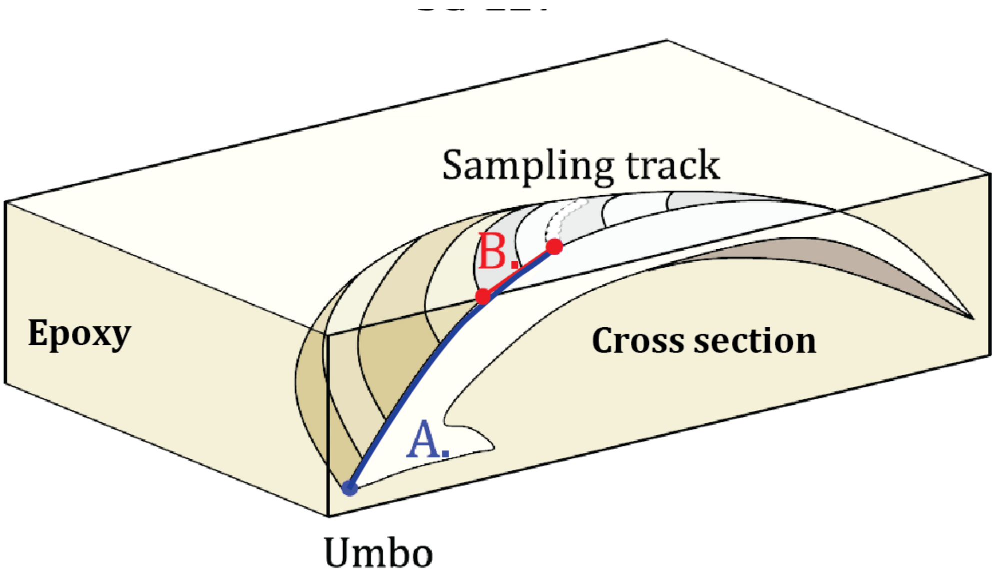

Fig. S3: Schematic overview of the result of the embedding procedure for specimen SG127 (*Angulus benedeni benedeni*). This workflow resulted in partial embedding of the shells in epoxy (translucent yellow), allowing the production of cross sections through the axis of maximum growth which can be sampled along track A (blue; within the outer shell layer) while leaving room on the outside of the shell for sampling along the growth increments visible on the outer shell layer (transect B, in red). Modified after (*67*).

2. Preservation screening

A combination of Reflected light microscopy, Scanning Electron Microscopy (SEM), cathodoluminescence microscopy (CL), Electron Backscatter Diffraction microscopy (EBSD), micro-X-ray fluorescence (µXRF) and X-ray Diffraction (XRD) were used to perform diagenetic screening. In the following, a selection of screening results is presented for each technique to demonstrate the high-quality state of preservation of the material from the Oorderen Member overall.

2.1 Reflected light microscopy

Entire cross sections through all shells were imaged in detail using a Keyence VHX digital microscope model VH-Z250R (Keyence Corp, Osaka, Japan) with magnification factors of 100-250x. Microscope images were stitched together using a combination of image-processing routines for automatic image stitching (ImageJ Grid Stitching(*81*) and Adobe Photoshop Photomerge algorithms; Adobe, San Jose, CA, USA) and hand-stitching of images for which algorithms did not perform well using Inkscape (GNU General Public License) and Adobe Illustrator (Adobe, San Jose, CA, USA). The aim was to create high-resolution cross-section images through the shells on which growth increments could be identified, serving as a guideline for sampling on the cross sections (see examples in **Fig. S4**). Some shells contained cracks (**Fig. S4A**) while others showed clear signs of bioerosion (dwelling burrows in the shell; see **Fig. S4B**). Areas affected by these disturbances were avoided during sampling.

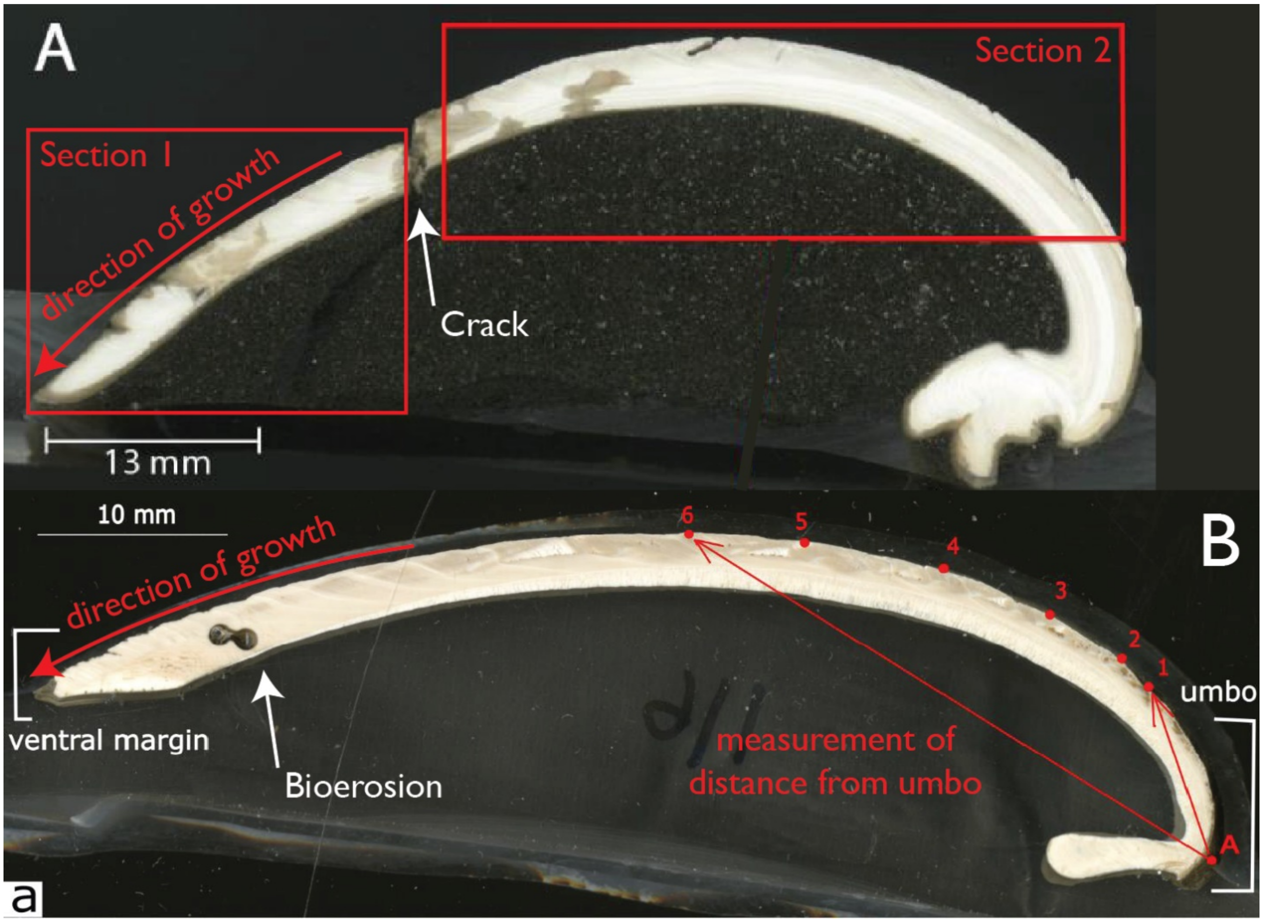

Fig. S4: High-resolution scans of cross sections through the shells of *Arctica islandica* (specimen SG107; A) and *Glycymeris radiolyrata* (specimen SG116; B). Red boxes in A highlight the two different sections of the shell of *A.islandica* that were sampled separately to avoid the crack in the middle of the specimen (white arrow). In *G. radiolyrata* (B), bioerosion is visible close to the ventral margin. Straight red arrows in B indicate how samples were referenced relative to their distance from the umbo, and the curved arrow in both panels highlights the direction of growth.

2.2 Scanning electron microscopy

Before SEM analysis, fragments of the ventral margin of fossil shells were prepared on round metal stubs and coated with a few-nanometer-thick carbon coating to prevent charging of the sample (following (*84*)). Micrographs at magnifications ranging from 70x to 650x were made using SEM of cross sections through the shells to study the texture and microstructure of the aragonite and check for signs of dissolution or reprecipitation of carbonate phases. The polished side of samples was studied along the growth direction, to assess the preservation of the internal shell structure. Fresh fracture surfaces were targeted to image the mineral structure in the shells. SEM was carried out on a JEOL JCM-6000 Versatile Benchtop SEM system (JEOL, Tokyo, Japan) operated under low-vacuum conditions at the Utrecht University GeoLab (following (*85*)).

An example of the SEM results of *Glycymeris radiolyrata* (SG116) is shown in **Fig. S5**. A location near the ventral margin of *G. radiolyrata* is examined more closely in three different areas: near the outer surface

(**Fig. S5a,d,g**), within the outer part of the outer shell layer (**Fig. S5b,e,h**) and within the inner part of the outer shell layer of the shell (**S5c,f,i**). The outer part of the outer shell layer is noticeably more massive than the inner outer shell layer. Noteworthy is the porosity of the inner growth lines in **Fig. S5i**. Porous spots are visible in the area just below the outer part of the outer shell layer (**Fig. S5b**), however not in a specific growth-line pattern.

SEM micrographs reveal that the inner outer shell layer of *G. radiolyrata* most likely contains a well-preserved crossed lamellar microstructure with original porosity and clear growth lamination (**Fig. S5c,f,i**). The outer outer shell layer is more massive, but equally well-preserved with shell microstructures closely resembling those observed in modern *Glycymeris* specimens (e.g. (*86*); see **Fig. S5d,e,g,h**). In **Fig. S5f**, the crystals seem to have a perpendicular orientation relative to each other, indicative of crossed-lamellar microstructure, as in modern *Glycymeris* species (e.g. (*87*)). No signs of recrystallization, evident from disruption of the regular shell microstructure by euhedral secondary calcite crystals (a.k.a. ”blocky calcite”; see e.g. (*88*)), were encountered in any of the studied specimens, attesting to their good preservation.

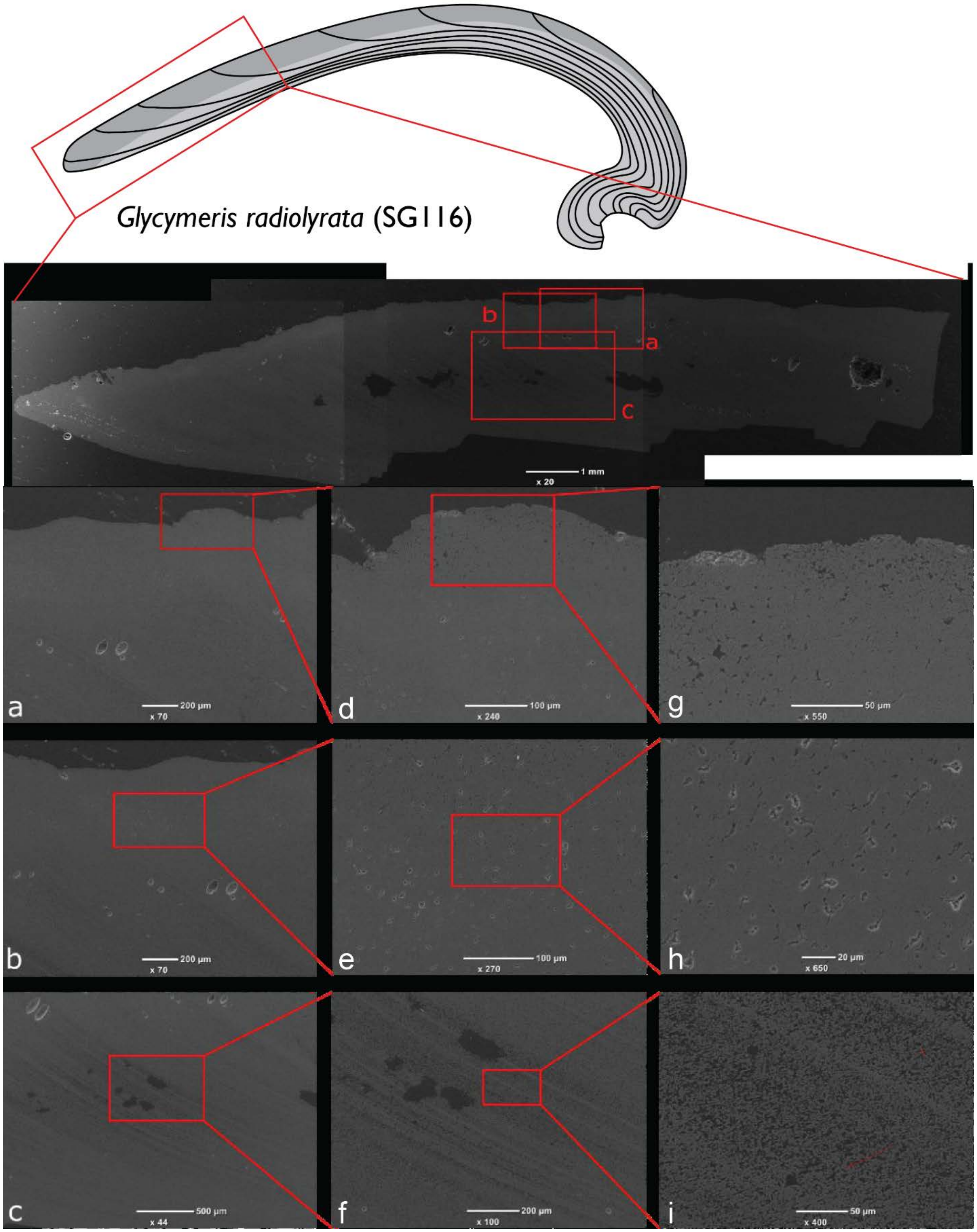

Figure S5: Overview of SEM images collected from specimen SG115 (*Glycymeris radiolyrata*) showing preservation of growth lines (**a-c**), original shell porosity (**d-i**) and variations in microstructure between inner and outer parts of the outer shell layer (**g-i**).

0.160 Cathodoluminescence microscopy

Fossil shell fragments were imaged using a Technosyn CL 8200 MK II cold-cathodoluminescence device (Cambridge Image Technology Ltd, Hatfield, UK) coupled to a Nikon D810 microscope and camera (Nikon Corp., Tokyo, Japan). Samples were excited using a cathode ray tube operated at 150-230 µA and 12-15 kV. In the process, areas on the sample with elevated concentrations of Mn^{2+} in the carbonate lattice exhibit luminescence, while higher concentrations of Fe^{2+} in the carbonate inhibit luminescence(*89*, *90*). This method therefore detects areas with higher concentrations of these trace metals, which are indicative of carbonates deposited under burial conditions and should be avoided in sampling for paleoclimate reconstructions(*91*, *92*).

Fig. S6a shows a composite of microscopy images in the hinge plate of *Arctica islandica* specimen SG107 under normal light circumstances, while **Fig. S6b** shows a CL-microscopy composite of the same sample area, revealing an absence of luminescence in the shell, material generally attesting to good preservation conditions. To make the different colored bands in **Fig. S6b** more noticeable, **Fig. S6c** was created using Adobe Photoshop (Adobe Inc., CA, USA) by enhancing the brightness by 80% and decreasing the contrast by 20%.

The clear green color in CL-microscopy images of *A. islandica* corresponds with the CL color signature of preserved aragonite(*90*). The subtle variability in CL-luminescence thus visualized follows the growth banding in the shell and is therefore interpreted to reflect small differences in the original trace element composition of the preserved aragonite of *A. islandica*. This variability was also observed in other studies of well-preserved aragonitic biomineral samples(*93*, *94*) and explained as oscillating zonation caused by differences between more porous and more compact zones(*94*). Notice that some detrital grains in the sediment surrounding the *A. islandica* specimen do show distinct luminescence, illustrating the CL signature of luminescent material contrasting with the overall poorly luminescent shell material (**Fig. S6b,c**).

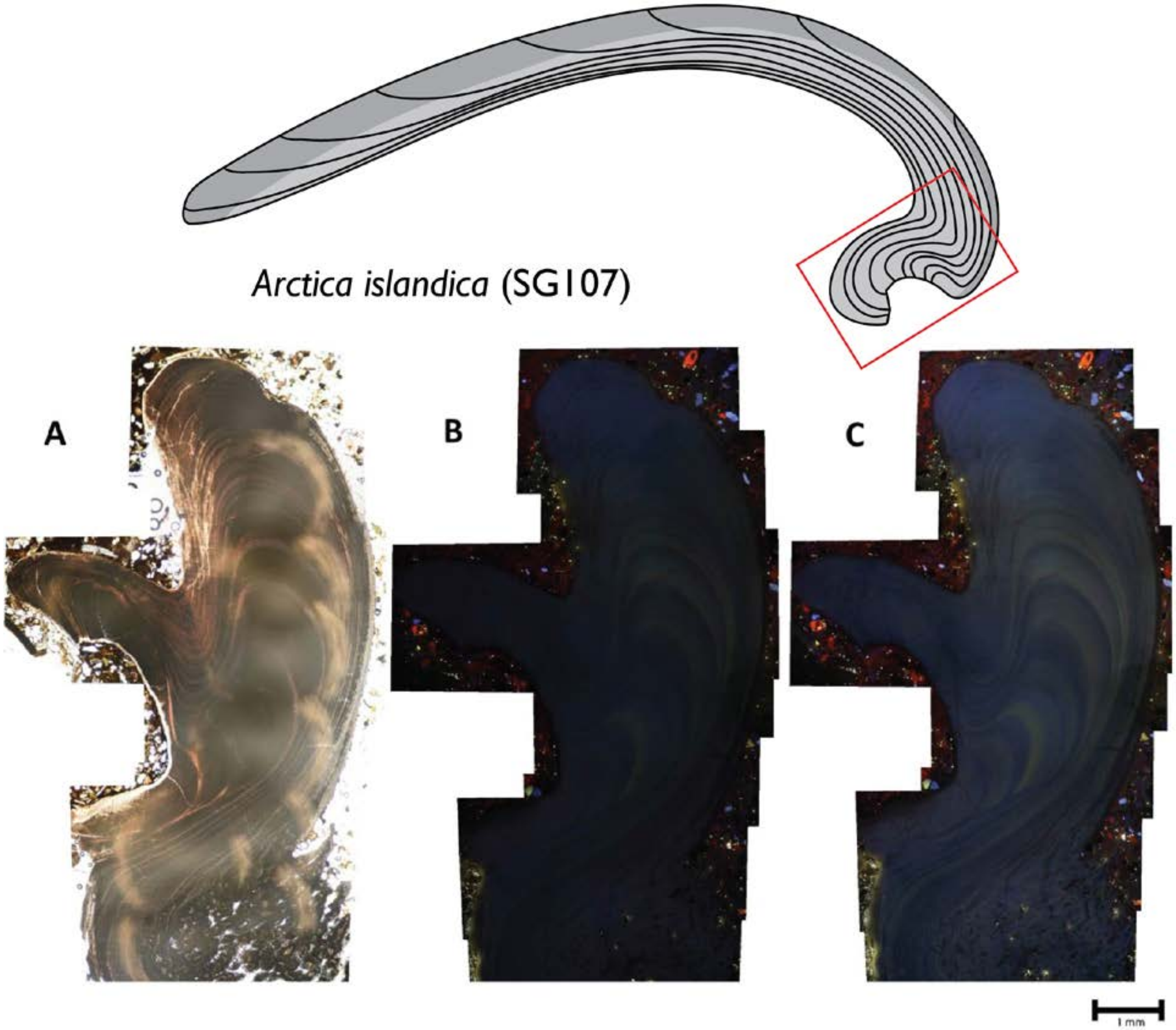

Fig. S6: Micrograph composite of a cross section through the hinge of *Arctica islandica* (specimen SG107). (**A**) A composite of the hinge plate with micrographs taken under normal light circumstances and the camera at 0.5 s shutter speed. (**B**) The untreated CL-microscopy composite of images created with the camera at 30s shutter speed. (**C**) The Photoshop-adjusted CL-microscopy view with brightness of +80% and contrast of -20%. A beam energy of 150 to 230 µA current and 12 to 15 kV was used to produce images **B** and **C**.

2.5 Electron Backscatter Diffraction microscopy

EBSD was used to map the mineral orientation in cross sections through fossil specimens and detect disturbances which highlight diagenetic alteration of the shell material. EBSD was carried out using an Oxford Instruments Symmetry EBSD detector (Oxford Instruments plc, Abingdon, UK) coupled to a Zeiss Gemini 450 SEM (Carl Zeiss AG, Jena, Germany) at the Utrecht University GeoLab (following methodology and instrument setup in (*67*)). Data handling was carried out in the Oxford Instruments AZtecCrystal software following (*67*) and maps of mineral orientation in selected shell sections are provided in **Figure S7-S11**.

In the EBSD maps, 0 to 0.2% of the area in *Angulus benedeni benedeni* specimens (SG126 & SG127) was classified as calcite. Upon inspection, these 'calcite' grains are actually wild spikes that were subsequently removed in the data clean-up (**Fig. S7**). There was no local secondary mineral growth visible in any map, which would have presented itself as larger crystals ('blocky calcite') that do not follow the surrounding structures(*95*, *96*). These results confirm the absence of diagenetic alteration of aragonite to calcite in *A. benedeni benedeni*. In some maps, there are shadows of specks of dust present, which locally prevent classification (**Fig. S7**). These 'dust shadows' are caused by dust that was present on the sample surface during analysis. Due to the high-angle orientation (70°) of the sample relative to the SEM electron beam, the specks cast relatively large shadows. In these areas, no diffraction can take place and thus no information is obtained there.

The aragonite in *A. benedeni benedeni* is very fine grained, with most crystals having an area of a few μm^2 or smaller (**Fig S8**; see also (*67*)). The crystals in *A. benedeni benedeni* exhibit a rotation around one axis. In SG-127, this is a rotation of the 001 and 010 axes around the 100 axis (**Fig. S8**). This results in two main crystal orientations parallel to two directions (X0 and Z0), and less variation parallel to the remaining direction (Y0).

The absence of 'blocky calcite' alone does not preclude diagenetic alteration, as calcite is not formed in the earliest stages of diagenesis(*84*, *97*, *98*) and there is not necessarily a difference in grain size (due to the presence of larger, secondary grains) between pristine and altered aragonite(*96*). However, none of the EBSD maps showed any evidence for diagenetic alteration. Microstructures that are similar to those found in other Tellinidae were observed in the EBSD maps of *Angulus benedeni benedeni*(*99*), suggesting that these are in fact original structures. Similarly, EBSD results from calcitic shells of *Ostrea edulis* (SG113; **Fig. S9**) and *Pecten complanatus* (SG110; **Fig. S10**) from the same strata reveal highly ordered crystal orientations resembling those observed in closely related modern relatives of these species (e.g. (*100*, *101*)).

In altered aragonitic bivalves, the structures as observed from EBSD maps are more homogeneous and have a less uniform density (as measured by the MUD score in EBSD; **Fig. S7-10**) compared to pristine material(*96*).The maps of altered shell material in (*96*) are much more homogeneous and 'randomized' than those in this study, which all exhibit a pattern. Based on the EBSD analysis, it is therefore assumed that no diagenetic alteration took place, and that the isotopic signal presented above is original and represents the formation temperature of the biogenic aragonite.

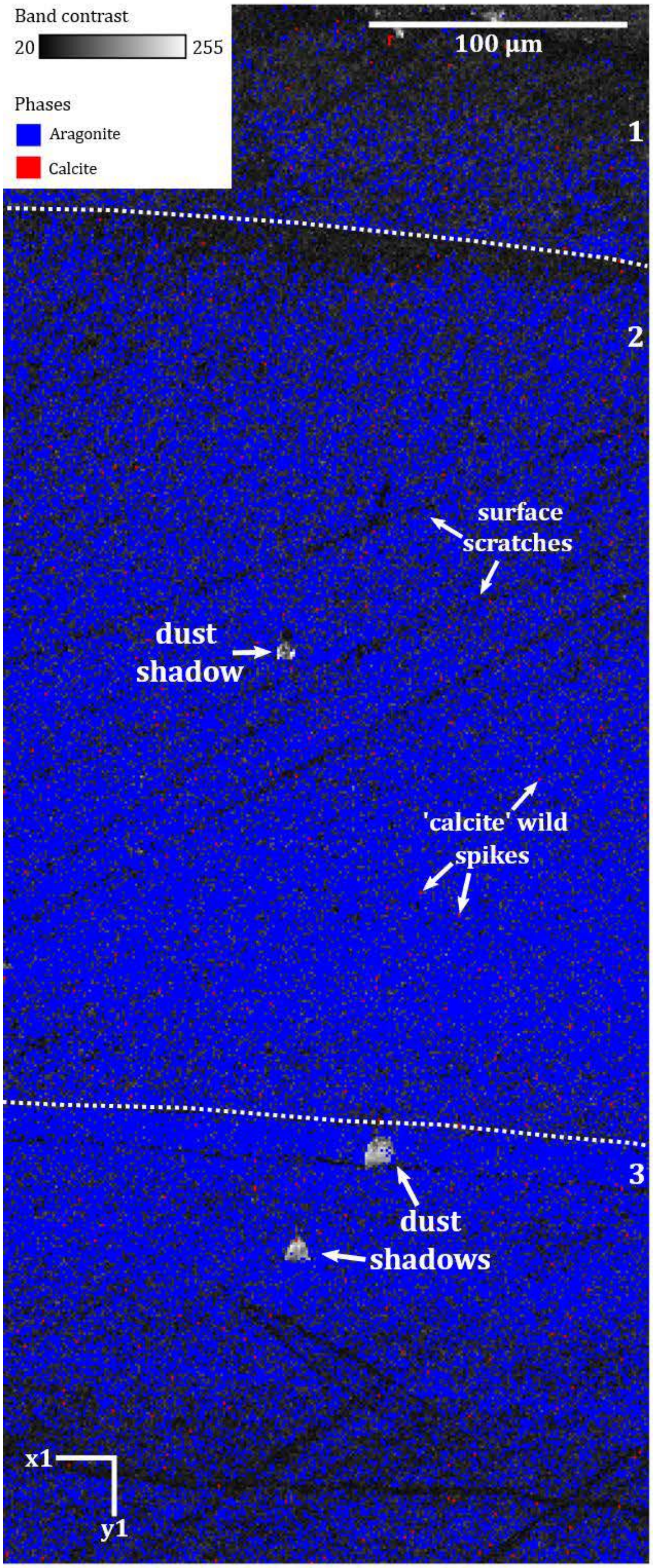

Fig. S7: EBSD map of *Angulus benedeni benedeni* showing the band contrast overlain by the classified pixels, which are either aragonite (blue) or calcite (red). The numbers 1, 2, and 3 correspond to the outer-outer shell layer, inner-outer shell layer and inner shell layer, respectively (see (*67*)). No data clean-up was done on this map. The pixels identified as calcite do not show any of the characteristics of actual secondary calcite (large crystals of "blocky calcite" compared to the very fine-grained aragonite, breaking up the aragonitic structure). They are always single pixels, and are removed through AztecCrystal's wild spike removal algorithm. Several dust shadows are also seen in this image; these areas are unclassified. There are also several scratches on the surface.

Map acquisition specifics: 10kV voltage, 1 µm step size. Measured at low vacuum due to excessive charging.

Classification: 54.6% aragonite, 45.2% unclassified, 0.2% calcite. Modified after (*67*).

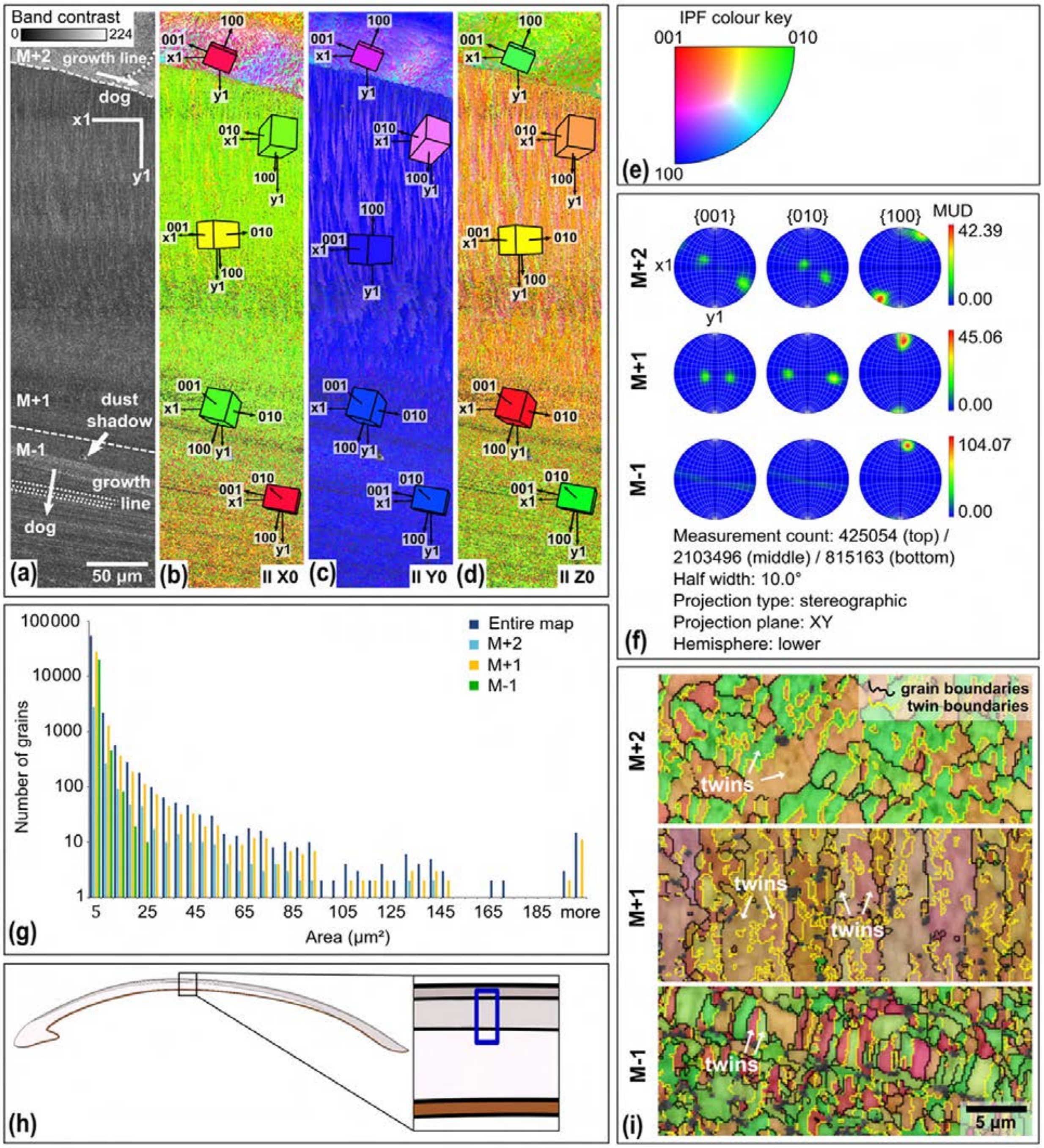

Fig. S8: EBSD map of specimen SG-127. Map acquisition specifics: 20 kV, high vacuum, 0.2 µm step size. (**a**) Band contrast map with scale, orientation, growth lines, direction of growth (dog), and outer-outer, inner-outer and inner shell layers separated by dashed lines. (**b**) EBSD inverse pole figure (IPF) X map, showing grain orientations parallel to the X0 axis. Unit cells of aragonite with the most common orientations are shown, the colour of which corresponds to the colour coding of the map. (**c**) EBSD IPF Y map showing grain orientations parallel to the Y 0 axis, with unit cells. (**d**) EBSD IPF Z map showing grain orientations parallel to the Z0 axis, with unit cells. I Inverse pole figure (IPF) colour key for the EBSD orientation maps. (**f**) Contoured pole figures for the three main crystallographic directions and the MUD,

for the different shell layers. (**g**) Grain size distribution chart for the entire map and the different shell layers. Note the logarithmic y axis. (**h**) Schematic figure indicating the approximate location of the map on the shell. (**i**) Shell layers from map IPF Z zoomed in and with added grain (black) and twinning (yellow) boundaries. Modified after (*67*).

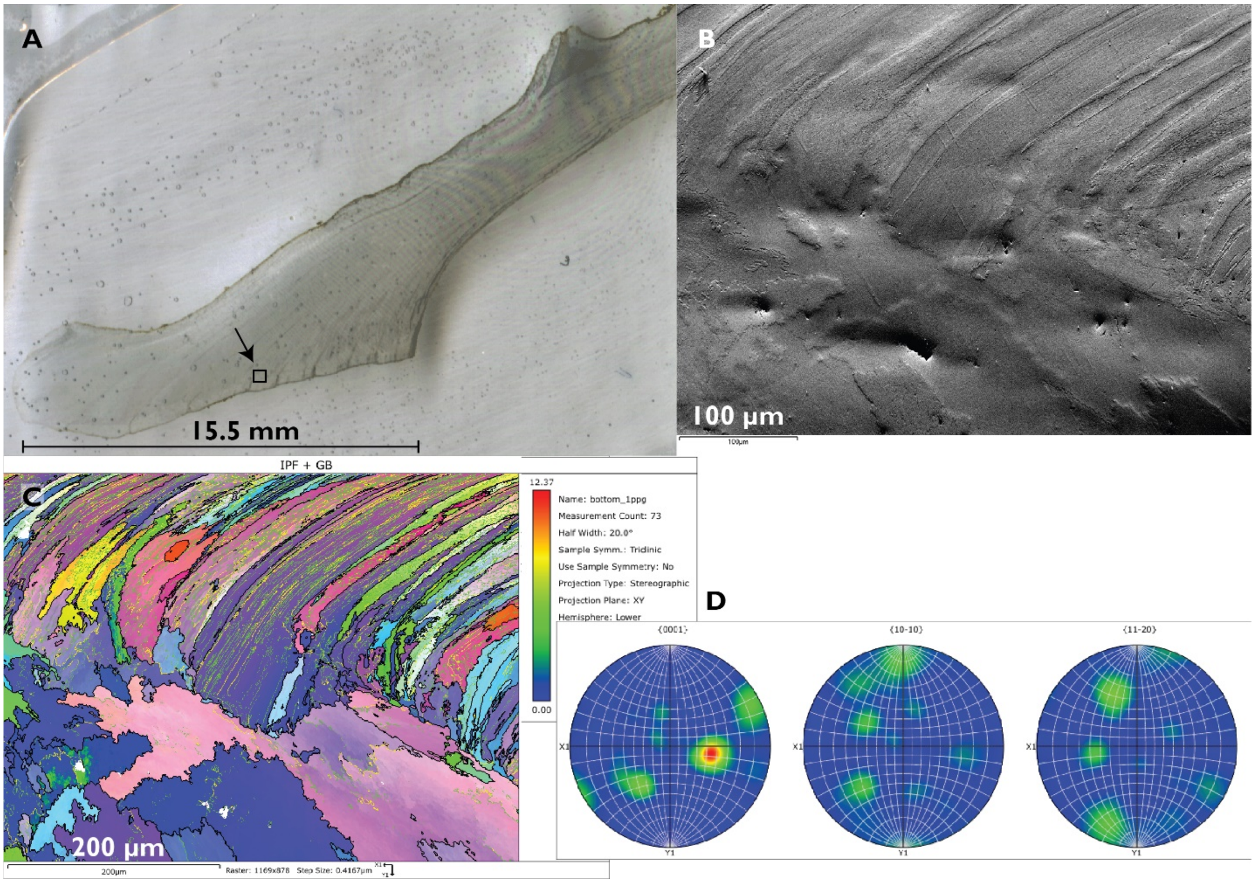

Fig. S9: Results of EBSD analysis on the hinge of *Ostrea edulis* specimen SG113. A) Scan of the thin section through the hinge prepared for EBSD analysis, with the black box showing the location of the EBSD map on the interface between a regular foliated calcite growth layer and the more disorganized structures near the hinge ligament. **B)** Forescatter image of the target area, highlighting sub-micrometer scale differences in shell density and relief (pixel size = 0.41 µm). **C)** EBSD map with structural boundaries identified by black lines. Different colors represent variability in the crystal orientation of the calcite within the target area. Notice the transition of regular, foliated calcite shell laminae (top of the image) to the unlaminated calcite in the ligament area close to the inner edge of the shell hinge. **D**) Density pole figures showing the orientation of major groups of crystals in the target area. Notice the regular clustering of crystallites indicating the regular orientation of originally preserved calcite crystals within the shell.

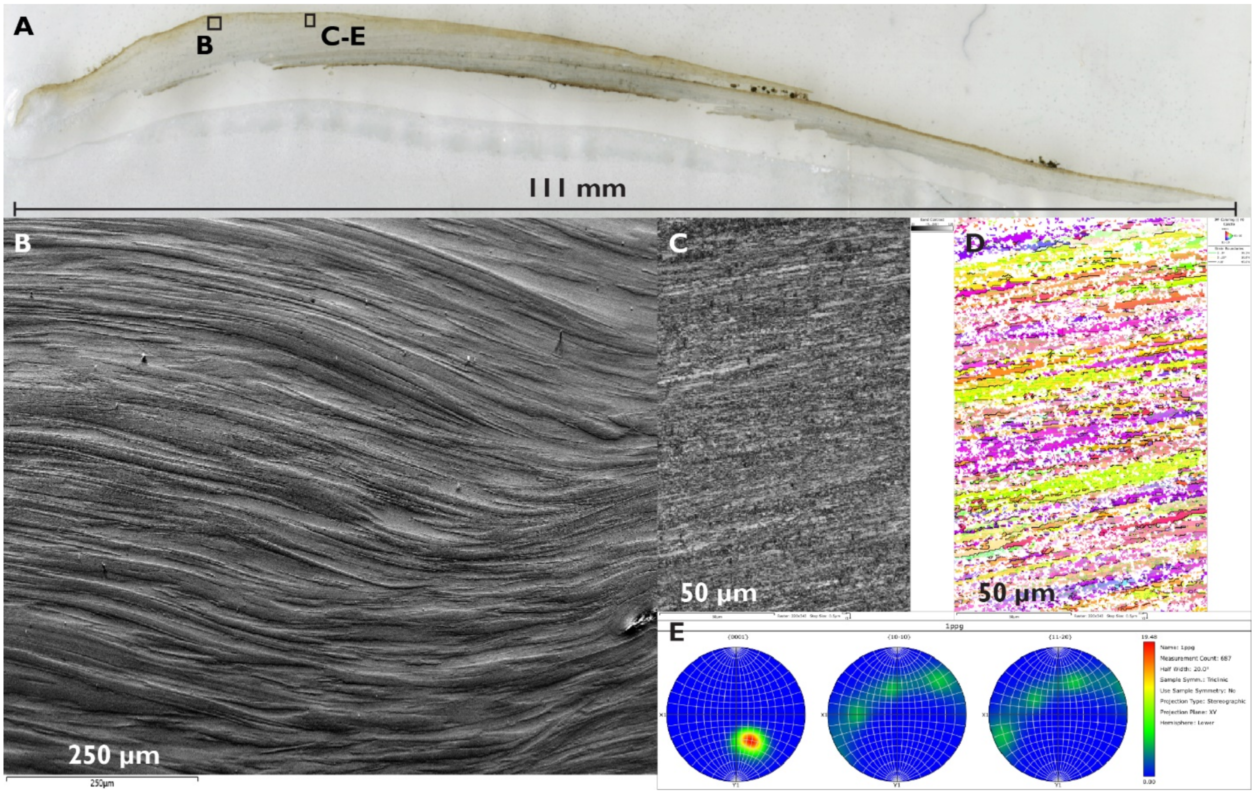

Fig. S10: Results of EBSD analysis of *Pecten maximus* from the Oorderen Member. A) Scan of the thin section through the shell prepared for EBSD analysis, with the black boxes showing the locations of the EBSD maps. **B)** Forescatter image of the left area, highlighting sub-micrometer scale differences in shell density and relief (pixel size = 0.5 µm). **C**) Forescatter image of the right area, showing regular banding of the calcite crystals in the outer shell layer. **D)** EBSD crystal orientations reflecting the regular banding of calcite crystals, hinting at well-preserved original shell material. Note that the crystallites are much smaller in this specimen than in **Fig. S7-9**, resulting in less complete identification of the pixels in the map and explaining the white spots in the EBSD figure. **E)** Density pole figures of crystal orientation in **D** confirm the regular orientation of crystals in three dimensions.

2.6 Micro-X-ray fluorescence trace element analysis

The trace element composition of a selection of fossil shells used in this study was determined using a Bruker M4 Tornado µXRF scanner (Bruker nano GmbH, Berlin, Germany) at the AMGC XRF platform of the Vrije Universiteit Brussel on polished shell cross sections. The M4 X-ray tube was operated at 50 kV and 600 µA under vacuum conditions following (*92*). Semi-quantitative XRF maps and quantitative XRF point measurements and line scans were used to locate areas in the shells with lower Sr concentrations and elevated Mn and Fe concentrations, which are indicative of diagenetic alteration. Quantitative measurements were carried out using longer X-ray beam integration times following recommendations in (*102*) and calibrated using matrix-matched carbonate standards following (*103*). The result of semi-quantitative XRF mapping of two specimens of *Glycymeris radiolyrata* (of which only one, SG116, is used in this study) and three specimens of *Angulus benedeni benedeni* (two of which were used for seasonality reconstructions) is presented in **Fig. S11**, while **Fig. S12b** gives an overview of the concentrations of Fe,

Mn and Sr measured in quantitative line scans through *A. benedeni benedeni* (specimens SG126 and SG127).

Quantitative micro-X-ray fluorescence (µXRF) point analysis supports the interpretation that no diagenetic recrystallization occurred in the specimens considered in this study. The sampled aragonite is high in Sr while low in Fe and Mn (**Fig. S12b**). Higher concentrations of Fe and Mn and lower concentrations of Sr in fossil carbonates are generally associated with pore water interaction during diagenetic alteration (e.g. partial recrystallisation) of the carbonate(*91*, *104*, *105*). The absence of high (>100 µg/g) Fe and Mn concentrations and presence of Sr concentrations similar to those observed in modern mollusk shell aragonite (e.g. (*105*, *106*)) suggest that this process did not take place in the specimens from the Oorderen Member.

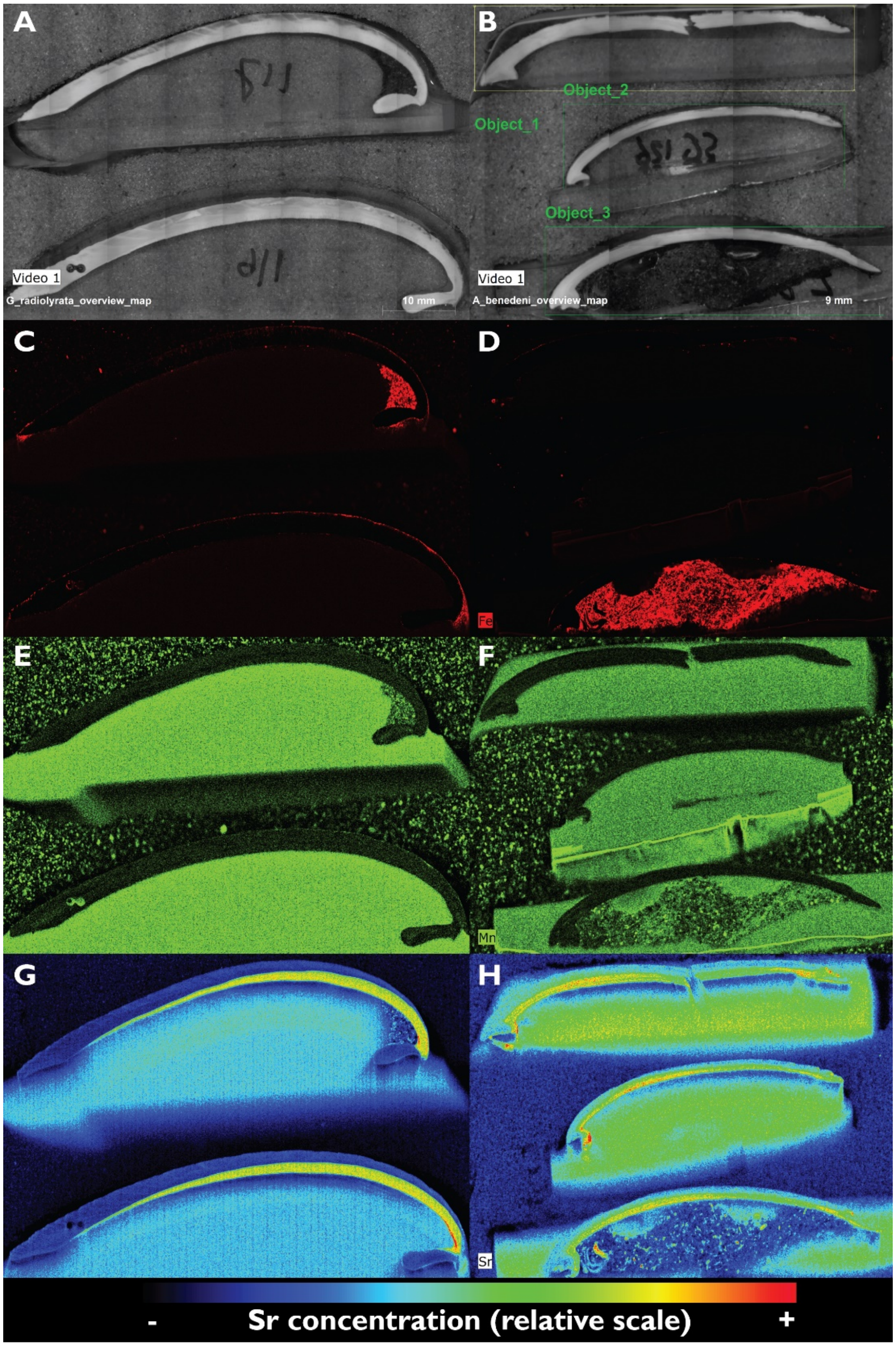

Fig. S11: XRF maps of *Glycymeris radiolyrata* (from top to bottom: specimens SG116 and SG117; **A**, **C**, **E** and **G**) and *Angulus benedeni benedeni* (from top to bottom: specimen SG125, SG126 and SG127; **B**, **D**, **F** and **H**). **A** and **B** show black and white images of cross sections through the specimens. **C**-**H** show semi-quantitative maps of Fe (**C** & **D**), Mn (**E** & **F**) and Sr (**G** & **H**) abundance through the shells. Color intensity in maps **C**-**F** shows the relative abundance of Fe (red) and Mn (green), while Sr concentration is indicated

in rainbow scale (see scale below). Note the absence of Fe and Mn and the high abundance of Sr in the outer shell layers as an indication of good preservation.

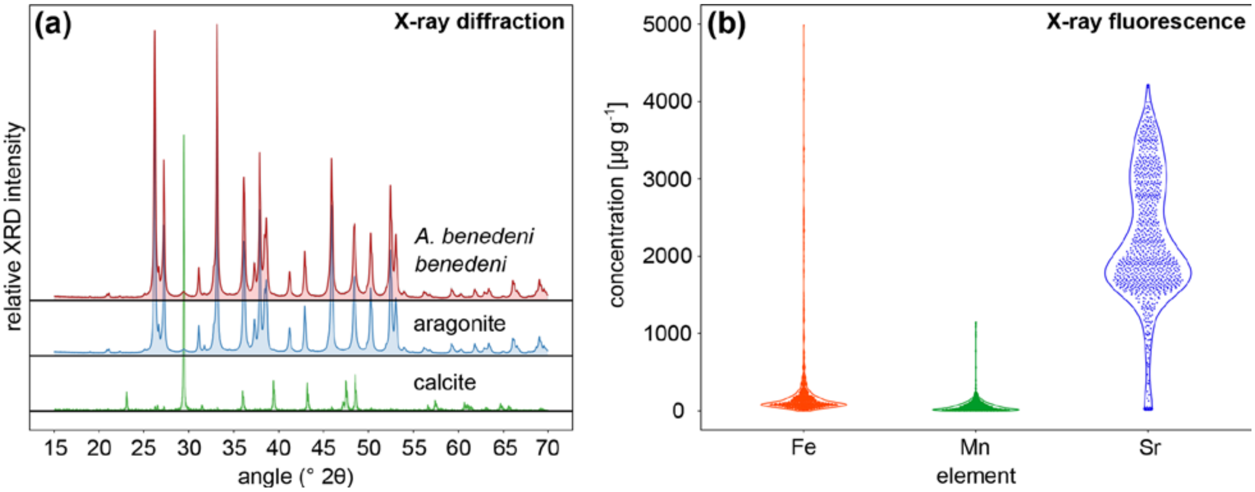

Fig. S12: X-ray diffraction (a) and X-ray fluorescence (b) analysis results from shell material of *Angulus benedeni benedeni*. XRD spectra are compared with patterns of pure aragonite and calcite, indicating an aragonitic composition of the shell (**a**). Violin plots in (**b**) show the results of the X-ray fluorescence analysis on *A. benedeni benedeni* for the elements Fe, Mn, and Sr. The "violin" shape depicts the kernel density function for the elemental concentration, plotted vertically and mirrored. The specimen is high in Sr and shows a broad distribution within these higher values. While there are some data points with high Fe and Mn values, the distribution is strongly skewed towards low values, showing overall low Fe and Mn values.

2.7 X-ray diffraction crystallography analysis

To check whether any aragonite in the fossil shells was converted to calcite during diagenesis, the mineralogy of bulk samples from aragonitic shells was analyzed using XRD. XRD analyses were carried out using a Bruker D8 Advance diffractometer at the Utrecht University GeoLab which was calibrated with a corundum crystal. The samples were ground to < 10µm in a Retsch McCrone mill with zirconium oxide grinding elements prior to front-loading it into a PMMA sample holder (diameter: 25 mm; depth: 1 mm). After removing the Cu Kα2 radiation component, results were compared to reference spectra for both calcite and aragonite from the RRUFF database(*107*). Instrument setup and data processing was done following (*67*). XRD analysis revealed that the shell of *Angulus benedeni benedeni* consists of 100% original aragonite, as its spectrum is identical to that of pure aragonite (**Fig. S12b**). This is strong evidence for the lack of diagenetic alteration of the specimens in this work, since aragonite is metastable under diagenetic conditions and transforms into calcite when these conditions affect the mineral composition of the shell(*105*).

3. Isotope analysis

3.1 Oxygen, carbon and clumped isotope analysis

In total, 663 aliquots of carbonate microsampled from fossil bivalves were analyzed for clumped isotope composition on two isotope ratio-mass spectrometers (Thermo MAT253 and MAT253 Plus, Thermo Fisher Scientific Inc., Waltham, MA, USA) coupled to Kiel IV carbonate preparation devices between June 2021 and December 2022. As a function of the varying stability of the instruments over the analytical period, between 70 µg and 160 µg of carbonate powder was required per analysis. Both instruments were operated in LIDI workflow(*69*, *70*) and samples were run in an approximately one-to-one ratio with ETH-1, ETH-2 and ETH-3 standards during analytical sessions(*73*). A small subset of 53 aliquots from *Pygocardia rustica* (SG105) was measured using sample-standard measurement cycles ("click-clack" mode; e.g. (*71*)) on the Thermo MAT253 to improve measurement stability during a short period of fluctuating lab environmental conditions (November-December 2022).

Carbonates were digested with 105% phosphoric acid ($H_3PO_4 + H_4P_2O_7$) at 70°C to produce CO_2, which was purified through two cryogenic (-170°C) traps and a Porapak trap(*69*) kept at -40°C to -50°C before being led into the mass spectrometer to analyze abundances of masses 44-49 against an in-house reference gas ($\delta^{18}O$ = -4.67 ‰VPDB; $\delta^{13}C$ = -2.82 ‰VPDB). Mass spectrometry intensity ratios were corrected for pressure baseline non-linearity using background scans before each analytical session(*72*), and the calculated δ values were corrected for ^{17}O concentration following (*74*). Clumped isotope (Δ_{47}) values were calculated as the abundance ratios of CO_2 masses 47 and 44 relative to the theoretical stochastic abundance given the isotopic composition of the sample following the formula:

$$\Delta_{47} = \left(\frac{R^{47}{}_{sample}}{R^{47}{}_{stoc}} - 1\right) * 1000‰ \text{(108, 109)}$$

In which R^{47} represents the ratio between the intensities of masses 47 and 44 in either the sample or the stochastic distribution. The ETH standards measured in the same analytical session as the samples as well as those measured during periods of roughly 2 weeks before and after the analysis were used to bring clumped isotope data into the I-CDES reference frame(*72*), accumulating a total of 6082 analyses (samples + standards) for the project. In some analytical sessions, a correlation was found between the intensity of the sample gas (measured on mass 44) and the Δ_{47} value. Therefore, samples were corrected with standards whose intensities where within 1 V from the intensity of the sample to prevent the corrected Δ_{47} value to be biased in case standards in the analytical window had on average lower or higher intensities than the sample.

Within each analytical session, IAEA-C2 (International Atomic Energy Agency, Vienna, Austria(*110*)) and Merck (Merck & CO., Rahway, NJ, USA; Catalog No. 1.02059.0050; lot no. B1164559 515(*111*, *112*)) reference materials were treated as samples to assess long-term measurement uncertainty. The $\delta^{18}O_c$ values for each sample were reported relative to the VPDB scale and calibrated using standards in the same analytical session to eliminate long-term trends in isotopic composition caused by instrument drift. Values from the InterCarb multi-lab comparison study were assumed as true values for all standards used in the (clumped) isotope calibration(*112*).

Clumped isotope analyses from specimens SG107, SG116, SG126 and SG127 were supplemented by a total of 237 previously measured $\delta^{18}O$ and $\delta^{13}C$ values from these specimens. These isotope analyses were measured using a Thermo Scientific GasBench II gas preparation system coupled to a Thermo Scientific

Delta V isotope ratio mass spectrometer (see description in (*67*)). The measurements have an external reproducibility (1 standard deviation) of 0.05 ‰ for $\delta^{13}C$ and 0.07 ‰ for $\delta^{18}O$ based on the in-house Naxos standard. Since this mass spectrometry setup did not allow us to analyze Δ_{47} values, these analyses only yielded $\delta^{18}O$ and $\delta^{13}C$ values. In cases where sufficient sample material was left Δ_{47}, $\delta^{18}O$ and $\delta^{13}C$ values were analyzed using the Kiel carbonate preparation device coupled to the Thermo MAT253 setup described above. In all other cases, $\delta^{18}O$ and $\delta^{13}C$ values which were not paired with Δ_{47} values from the same measurement were only used to refine the ShellChron age model (see below), and did not contribute to the reconstructions described in this work.

3.2 Data processing

Outliers were removed from the dataset if they met one of the following criteria:

1. The intensity of the sample gas in the mass spectrometer was outside the 10 – 25 V range.
2. The intensity on mass 49 (which is rare in natural CO_2 gases), measured as the 49/44 mass ratio relative to the working gas, exceeded 0.01‰, indicating contamination in the sample.
3. The standard deviation between Δ_{47} values calculated from different CO_2 pulses from the same sample (internal standard deviation) exceeded 0.15‰.
4. The Δ_{47}, $\delta^{18}O_c$ or $\delta^{13}C$ values measured in standard reference materials deviated significantly (>3 standard deviations) from their accepted values.

Standards and samples meeting these criteria were not considered for further analysis. Datasets from different instruments (MAT253 vs MAT253 Plus) and using different methods (LIDI vs "click-clack") were separately corrected for long-term drift by plotting the offset of standard measurements from their accepted values over time and applying a loess filter to identify trends (see step 1 in supplement). After correction, the external standard deviation on Δ_{47}, $\delta^{18}O_c$ or $\delta^{13}C$ values was calculated separately per instrument dataset from the independent IAEA-C2 and Merck standards (see **Table S2**) before merging the datasets from both instruments (see step 2 in online supplement).

Reference material	Instrument	Method	N	Δ_{47}		$\delta^{18}O_c$		$\delta^{13}C$	
				μ	σ	μ	σ	μ	σ
ETH-1	MAT2“3	"click-cl”ck"	71	0.206	0.033	-2.24	0.14	2.01	0.05
ETH-2	MAT2“3	"click-cl”ck"	72	0.227	0.042	-18.64	0.07	-10.11	0.04
ETH-3	MAT2“3	"click-cl”ck"	226	0.607	0.038	-1.78	0.15	1.69	0.07
IAEA-C2	MAT2“3	"click-cl”ck"	22	0.650	0.041	-8.95	0.06	-8.12	0.03
Merck	MAT2“3	"click-cl”ck"	21	0.543	0.041	-15.57	0.06	-41.66	0.22
ETH-1	MAT253	LIDI	265	0.230	0.048	-2.24	0.10	2.03	0.06
ETH-2	MAT253	LIDI	302	0.217	0.047	-18.78	0.09	-10.18	0.05
ETH-3	MAT253	LIDI	1001	0.604	0.046	-1.74	0.08	1.71	0.06
IAEA-C2	MAT253	LIDI	84	0.641	0.050	-9.03	0.09	-8.17	0.14
Merck	MAT253	LIDI	70	0.525	0.069	-15.72	0.14	-42.00	0.13
ETH-1	MAT253 Plus	LIDI	337	0.221	0.042	-2.28	0.16	2.02	0.06
ETH-2	MAT253 Plus	LIDI	367	0.226	0.044	-18.83	0.17	-10.17	0.06
ETH-3	MAT253 Plus	LIDI	1998	0.607	0.040	-1.78	0.13	1.71	0.06
IAEA-C2	MAT253 Plus	LIDI	123	0.627	0.036	-9.05	0.17	-8.15	0.09
Merck	MAT253 Plus	LIDI	96	0.516	0.044	-15.72	0.19	-41.95	0.16

Table S2: **Mean values and external standard deviations of oxygen, carbon and clumped isotope measurements on standards ETH-1, ETH-2, ETH-3, IAEA-C2 and Merck calculated per instrument and per method.**

The resulting corrected $\delta^{18}O_c$ or $\delta^{13}C$ values were plotted against shell height to inspect seasonal variability in shell composition (see step 3 in online supplement). Based on periodic variability in the $\delta^{18}O_c$ values and the presence of visual growth breaks in the shells, annual cycles in shell growth were identified and marked to form a starting point for isotope-based age modelling. The resulting plots are provided in **Figures S13-S19**.

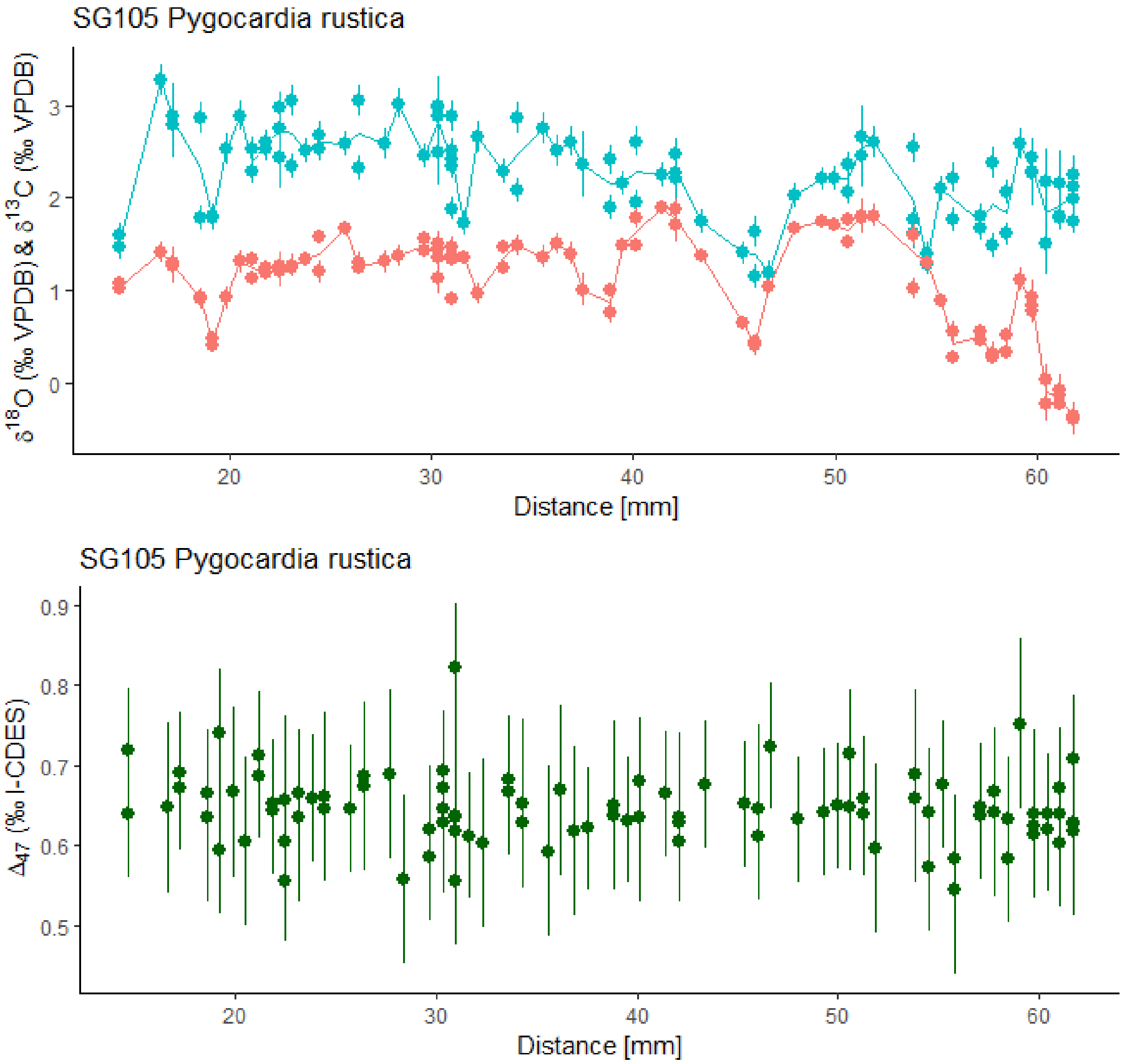

Fig. S13: **Stable and clumped isotope results for *Pygocardia rustica* specimen SG105** Stable carbon isotope results ($\delta^{13}C$ values; in red), oxygen isotope results ($\delta^{18}O$ values; in blue) and clumped isotope values (Δ_{47}; in green) for *Pygocardia rustica* specimen SG105 plotted against shell height (Distance) along the axis of maximum growth in the direction of ontogeny (samples with low shell heights deposited early in the life of the animal). Lines in the same color show 3-point moving averages through the $\delta^{18}O$ and $\delta^{13}C$ records. Vertical lines through the datapoints represent measurement uncertainties (+/- 2 standard deviations) on individual aliquots based on the check standard IAEA-C2. Note that the external standard deviation on individual Δ_{47} values is too high to prevent interpreting these individual aliquots in terms of

temperature. This issue is overcome by statistically pooling all aliquots within a given season and propagating the measurement uncertainties into confidence intervals (see explanation below).

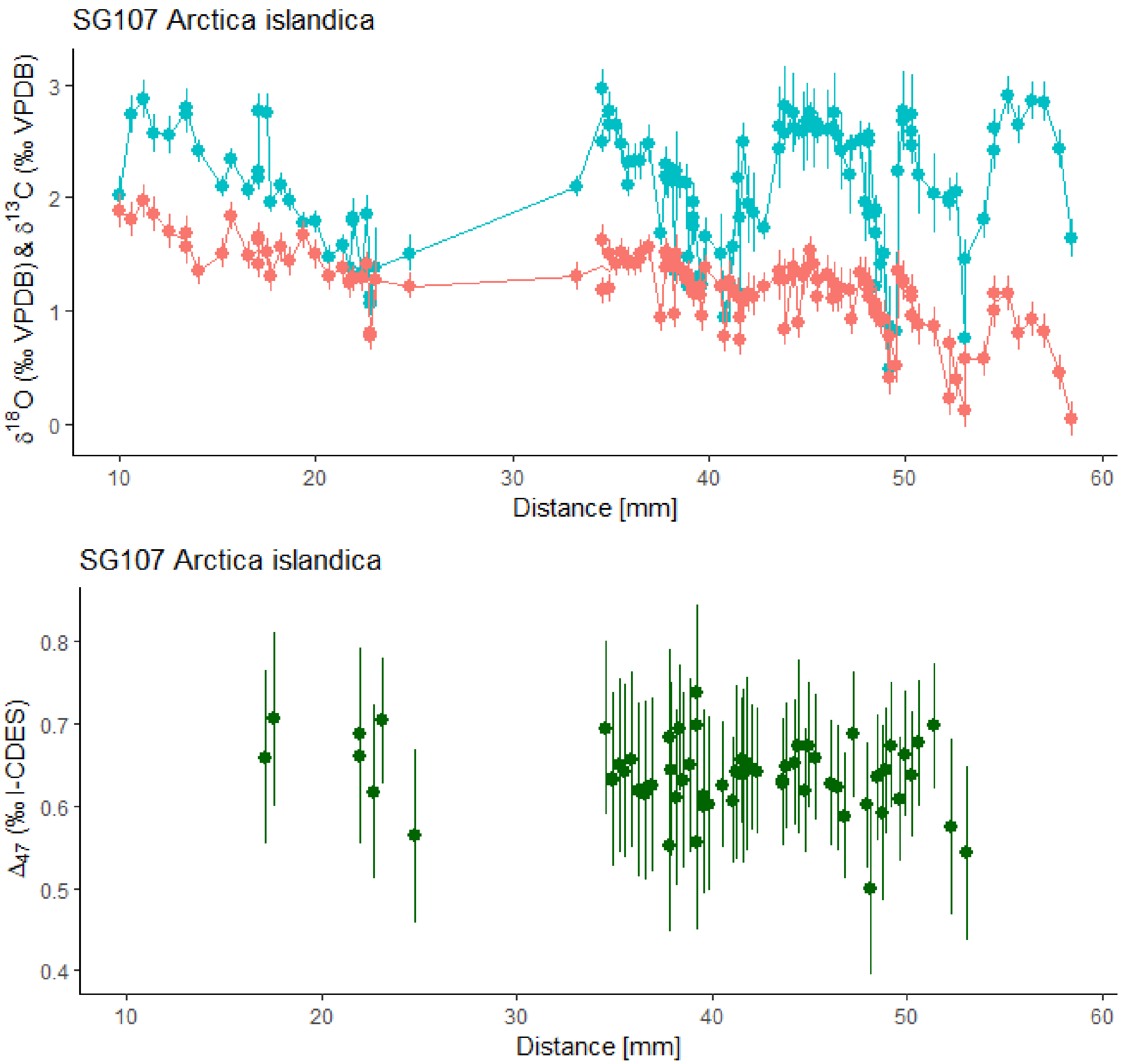

Fig. S14: **Stable and clumped isotope results for *Actica islandica* specimen SG107.** Stable carbon isotope results (δ^{13}C values; in red), oxygen isotope results (δ^{18}O values; in blue) and clumped isotope values (Δ_{47}; in green) for *Arctica islandica* specimen SG107 plotted against she height (Distance) along the axis of maximum growth. Lines in the same color show 3-point moving averages through the isotope measurements. Note that the hiatus around 25-30 mm was caused by a crack in the shell (see **Fig. S4**) around which sampling was avoided to prevent the risk of including diagenetically altered material in the analyses. Sample locations with δ^{18}O and δ^{13}C values without Δ_{47} values represent samples analyzed using the GasBench routine described above (see section 3.1) and were only used for age modelling. Vertical lines through the datapoints represent measurement uncertainties (+/- 2 standard deviations) on individual aliquots based on the check standard IAEA-C2. Note that the external standard deviation on individual Δ_{47} values is too high to prevent interpreting these individual aliquots in terms of temperature.

This issue is overcome by statistically pooling all aliquots within a given season and propagating the measurement uncertainties into confidence intervals (see explanation below).

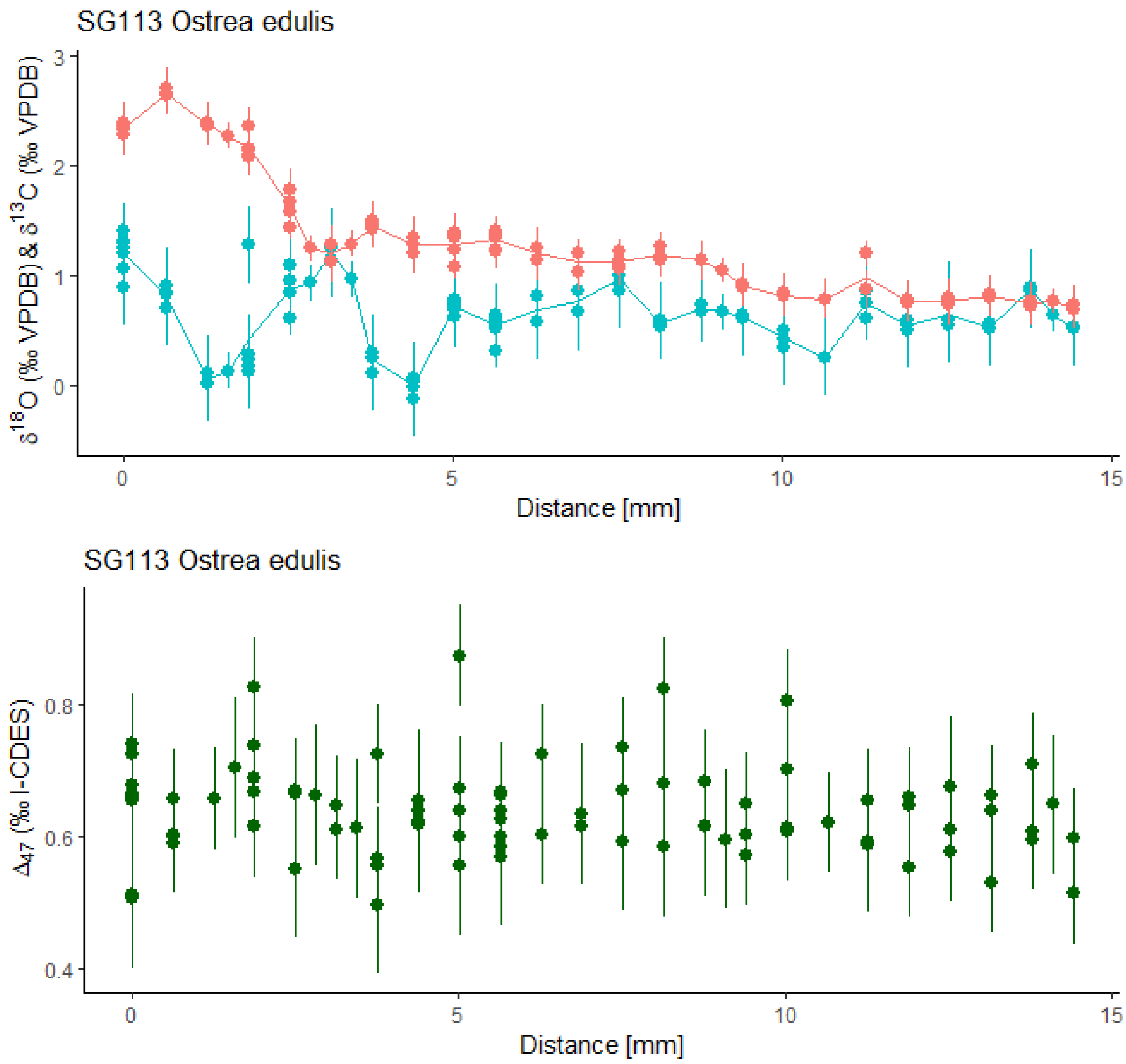

Fig. S15: **Stable and clumped isotope results for *Ostrea edulis* specimen SG113.** Stable carbon isotope results ($\delta^{13}C$ values; in red), oxygen isotope results ($\delta^{18}O$ values; in blue) and clumped isotope values (Δ_{47}; in green for *Ostrea edulis* specimen SG113 plotted against shell height (Distance) along the axis of maximum growth through the shell hinge plate. Lines in the same color show 3-point moving averages through the isotope measurements. Vertical lines through the datapoints represent measurement uncertainties (+/- 2 standard deviations) on individual aliquots based on the check standard IAEA-C2. Note that the external standard deviation on individual Δ_{47} values is too high to prevent interpreting these individual aliquots in terms of temperature. This issue is overcome by statistically pooling all aliquots within a given season and propagating the measurement uncertainties into confidence intervals (see explanation below).

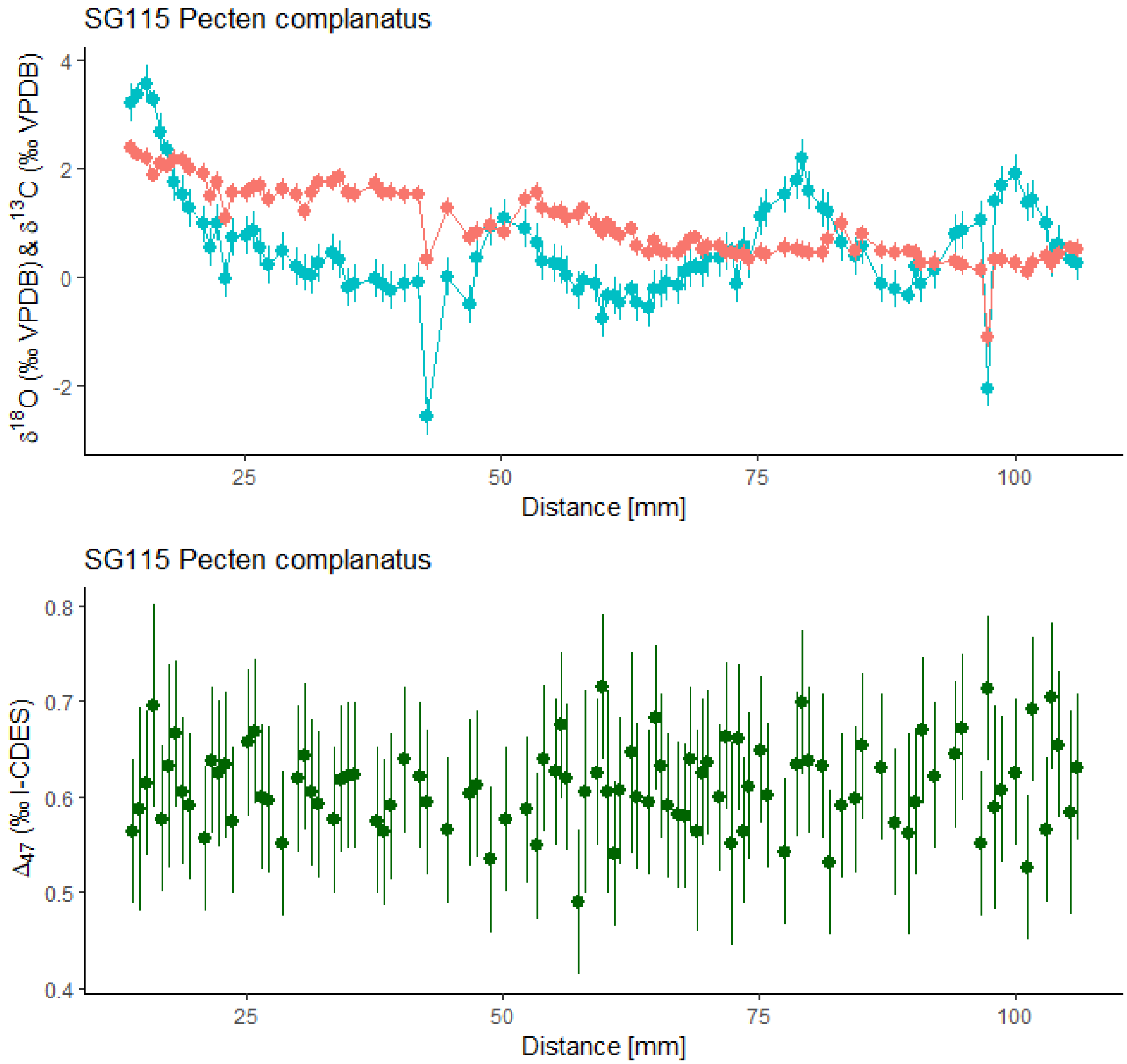

Fig. S16: **Stable and clumped isotope results for *Pecten complanatus* specimen SG115.** Stable carbon isotope results ($\delta^{13}C$ values; in red), oxygen isotope results ($\delta^{18}O$ values; in blue) and clumped isotope values (Δ_{47}; in green) for *Pecten complanatus* specimen SG115 plotted against shell height (Distance) along the axis of maximum growth in the shell. Lines in the same color show 3-point moving averages through the isotope measurements. Note the two negative outliers at ~40 mm and ~95 mm, which were removed for further analysis. Vertical lines through the datapoints represent measurement uncertainties (+/- 2 standard deviations) on individual aliquots based on the check standard IAEA-C2. Note that the external standard deviation on individual Δ_{47} values is too high to prevent interpreting these individual aliquots in terms of temperature. This issue is overcome by statistically pooling all aliquots within a given season and propagating the measurement uncertainties into confidence intervals (see explanation below).

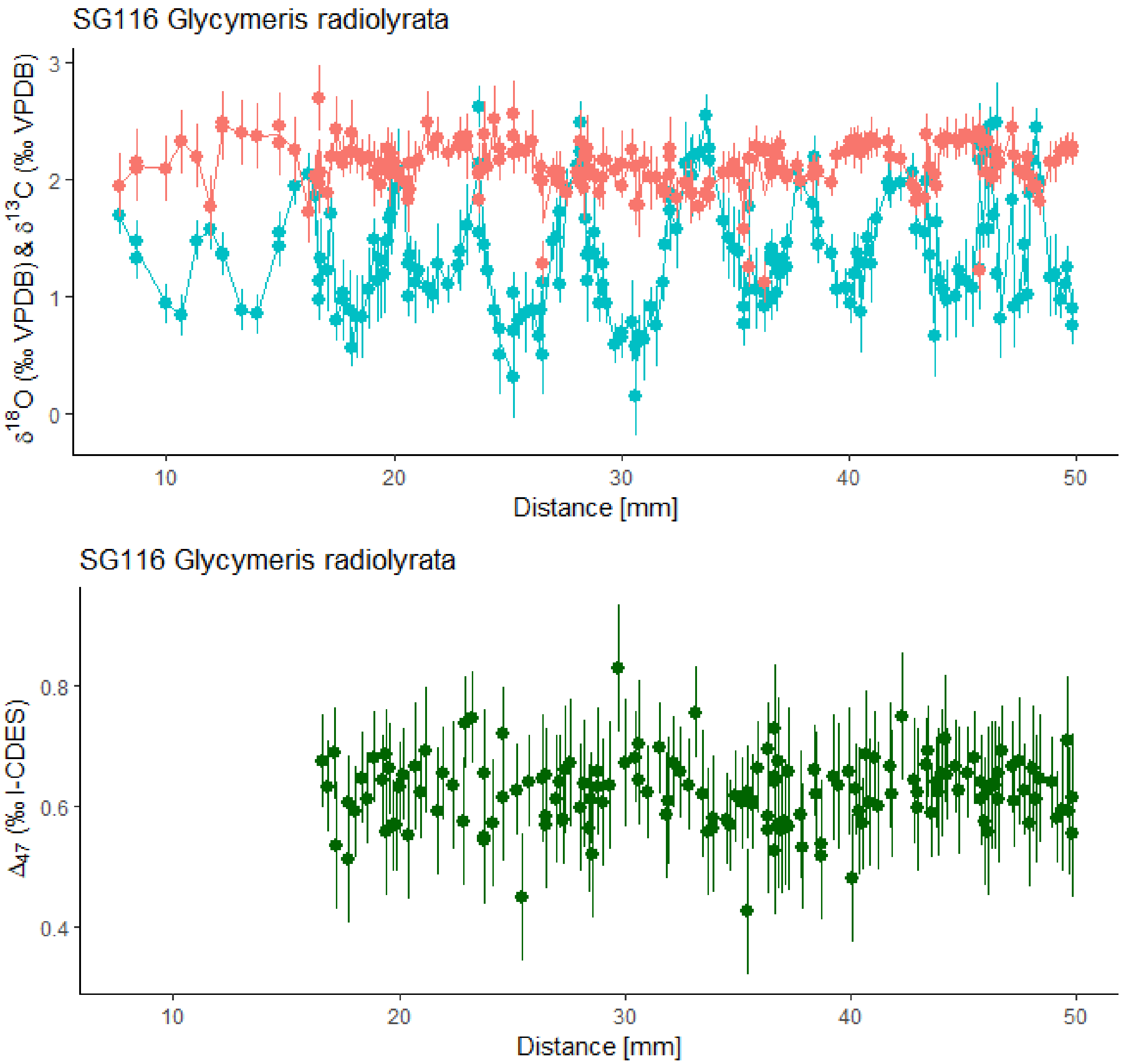

Fig. S17: **Stable and clumped isotope results for *Glycymeris radiolyrata* specimen SG116.** Stable carbon isotope results ($\delta^{13}C$ values; in red), oxygen isotope results ($\delta^{18}O$ values; in blue) and clumped isotope values (Δ_{47}; in green) for *Glycymeric radiolyrata* specimen SG116 plotted against shell height (Distance) along the axis of maximum growth in the shell. Lines in the same color show 3-point moving averages through the isotope measurements. Sample locations with $\delta^{18}O$ and $\delta^{13}C$ values without Δ_{47} values represent samples analyzed using the GasBench routine described above and were only used for age modelling. Vertical lines through the datapoints represent measurement uncertainties (+/- 2 standard deviations) on individual aliquots based on the check standard IAEA-C2. Note that the external standard deviation on individual Δ_{47} values is too high to prevent interpreting these individual aliquots in terms of temperature. This issue is overcome by statistically pooling all aliquots within a given season and propagating the measurement uncertainties into confidence intervals (see explanation below).

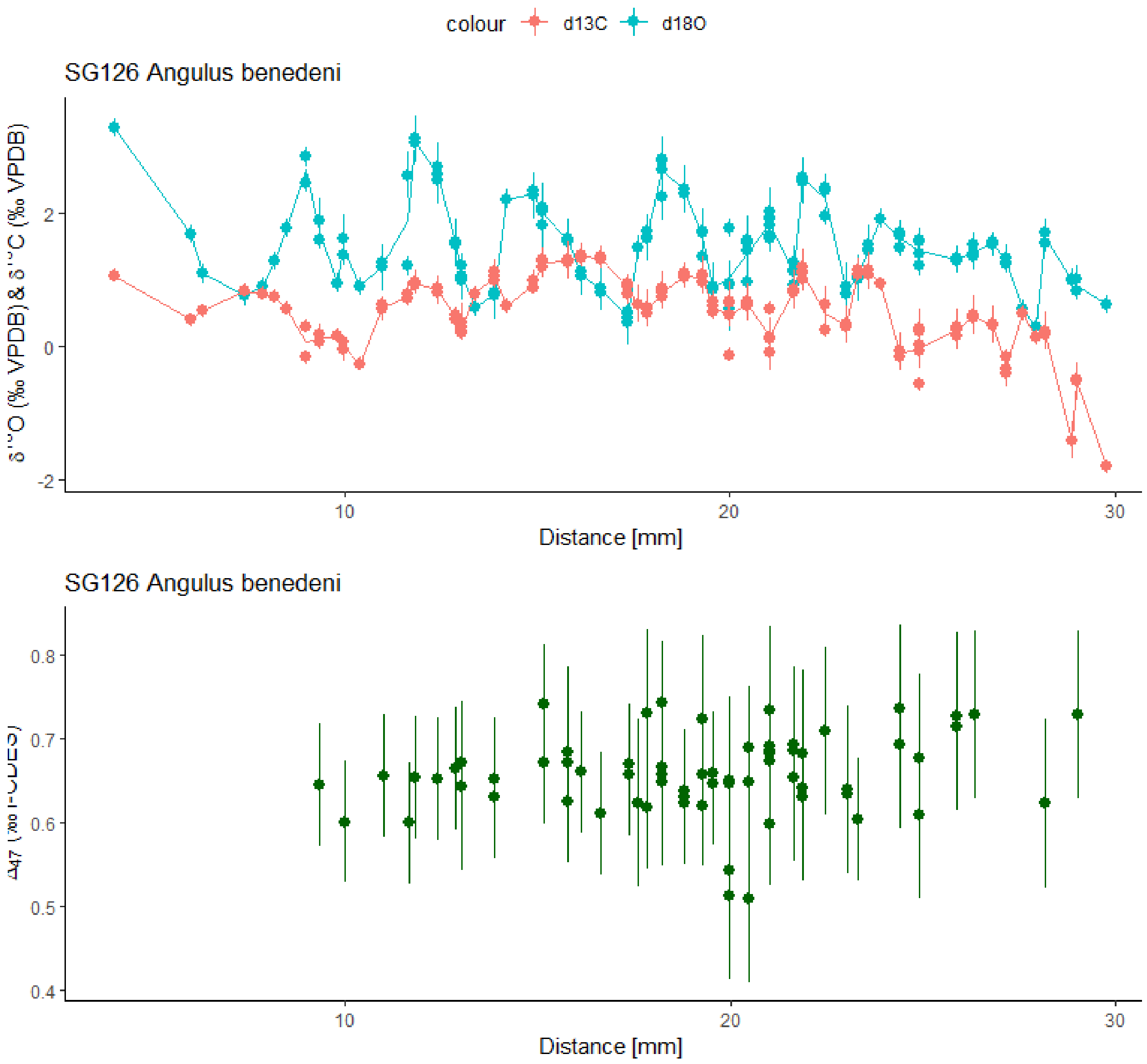

Fig. S18: **Stable and clumped isotope results for *Angulus benedeni benedeni* specimen SG126.** Stable carbon isotope results ($\delta^{13}C$ values; in red), oxygen isotope results ($\delta^{18}O$ values; in blue) and clumped isotope values (Δ_{47}; in green) for *Angulus benedeni benedeni* specimen SG126 plotted against shell height (Distance) along the axis of maximum growth in the shell. Lines in the same color show 3-point moving averages through the isotope measurements. Sample locations with $\delta^{18}O$ and $\delta^{13}C$ values without Δ_{47} values represent samples analyzed using the GasBench routine described above and were only used for age modelling. Vertical lines through the datapoints represent measurement uncertainties (+/- 2 standard deviations) on individual aliquots based on the check standard IAEA-C2. Note that the external standard deviation on individual Δ_{47} values is too high to prevent interpreting these individual aliquots in terms of temperature. This issue is overcome by statistically pooling all aliquots within a given season and propagating the measurement uncertainties into confidence intervals (see explanation below).

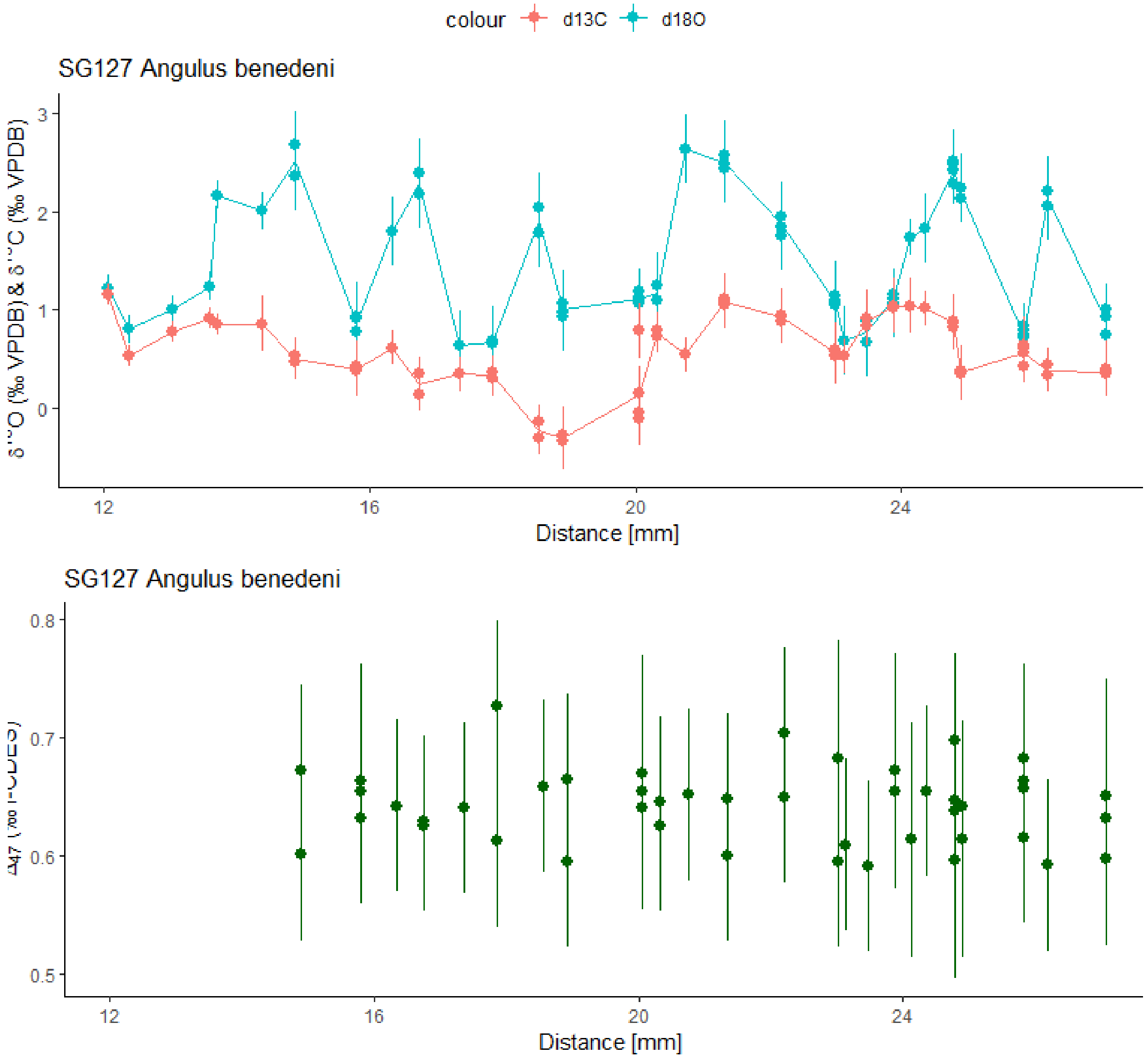

Fig. S19: **Stable and clumped isotope results for *Angulus benedeni benedeni* specimen SG127.** Stable carbon isotope results ($\delta^{13}C$ values; in red), oxygen isotope results ($\delta^{18}O$ values; in blue) and clumped isotope values (Δ_{47}; in green) for *Angulus benedeni benedeni* specimen SG127 plotted against shell height (Distance) along the axis of maximum growth in the shell. Lines in the same color show 3-point moving averages through the isotope measurements. Sample locations with $\delta^{18}O$ and $\delta^{13}C$ values without Δ_{47} values represent samples analyzed using the GasBench routine described above and were only used for age modelling. Vertical lines through the datapoints represent measurement uncertainties (+/- 2 standard deviations) on individual aliquots based on the check standard IAEA-C2. Note that the external standard deviation on individual Δ_{47} values is too high to prevent interpreting these individual aliquots in terms of temperature. This issue is overcome by statistically pooling all aliquots within a given season and propagating the measurement uncertainties into confidence intervals (see explanation below).

3.3 Internal chronologies

Samples were dated relative to the seasonal cycle by applying the ShellChron algorithm(*75*) on $\delta^{18}O_c$ profiles through the shells (see step 4 in online supplement). The results of age modelling are plotted in **Figures S20-S26** and provided in the online supplement.

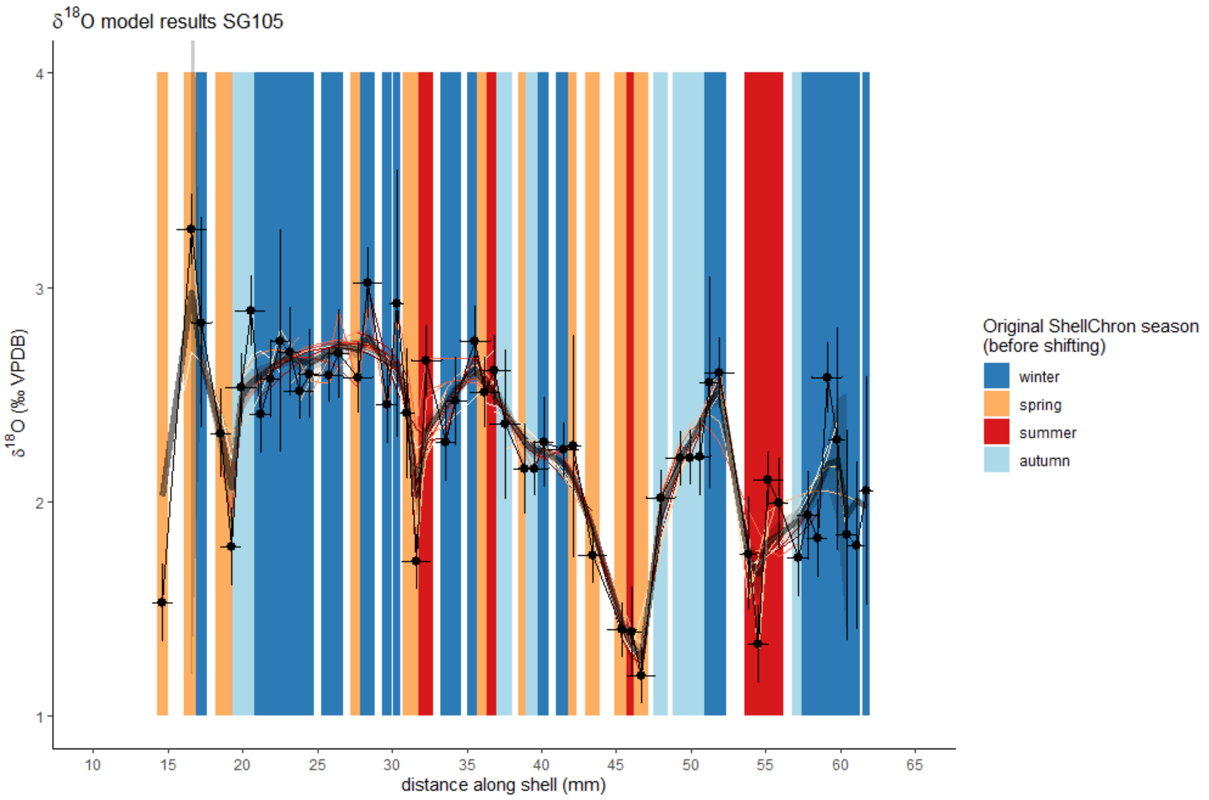

Fig. S20: ShellChron oxygen isotope-based age modelling result for *Pygocardia rustica* specimen SG105. Original oxygen isotope data are shown as black dots with vertical and horizontal lines denoting two standard deviations of uncertainty. Data is averaged per sample where multiple aliquots were measured for the same sampling distance. Modelled $\delta^{18}O_c$ profile is plotted as a thick grey line with lighter grey 95% CL error envelope. Thinner lines in shaded red colors highlight the results of overlapping modelling windows fitting simulated $\delta^{18}O_c$ data to the measurements (see (*75*)). Background colors highlight the season assigned to each datapoint based on the original ShellChron result, assuming days 46 – 136 (1/8 – 3/8 of the year) represent spring, days 137 – 228 (3/8 – 5/8 of the year) represent summer, days 229 – 319 (5/8 – 7/8 of the year) represent autumn and days 1 – 45 and 320 – 365 (0 – 1/8 and 7/8 – end of the year) represent winter. Note that this is the original age assignment before shifting the seasonal assignment according to the clumped isotope results (see section 3.3).

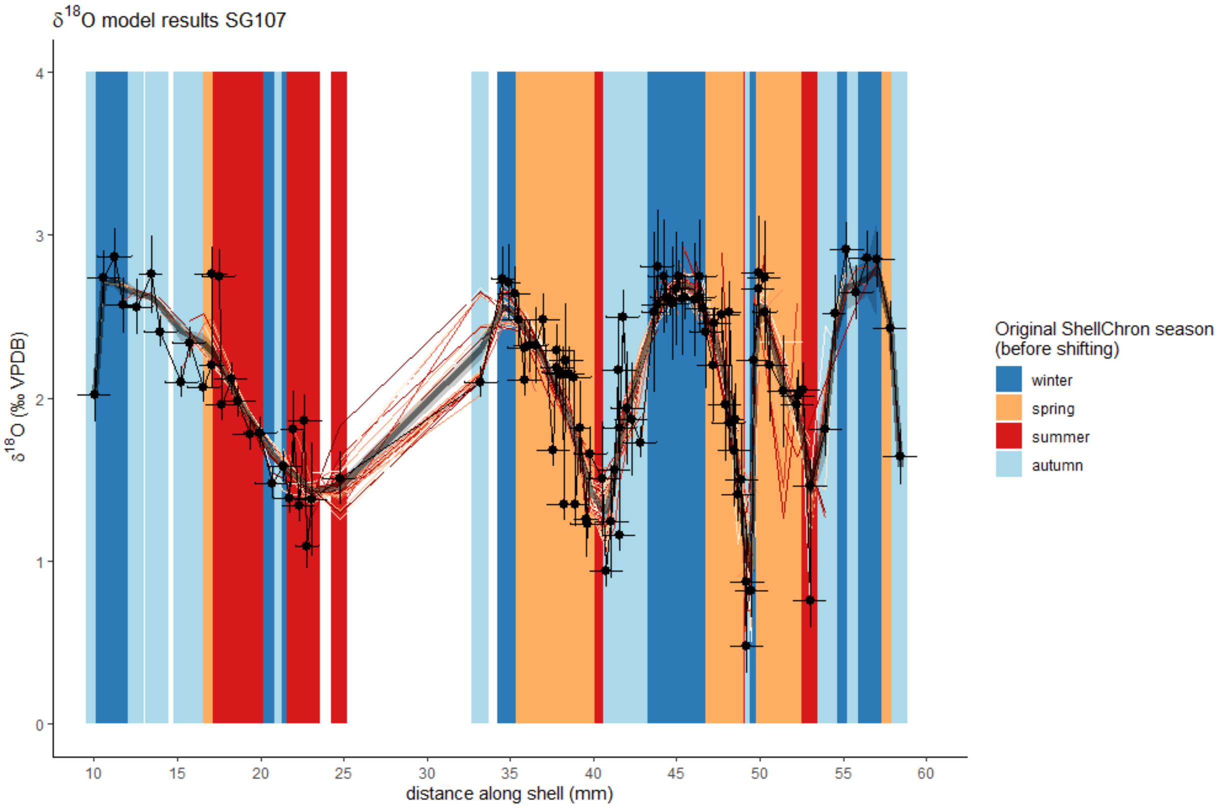

Fig. S21: **ShellChron oxygen isotope-based age modelling result for *Arctica islandica* specimen SG107.** Colors and symbols as in **Fig. S20**. Note the gap in the data due to the crack in the shell (see caption of **Fig. S14**).

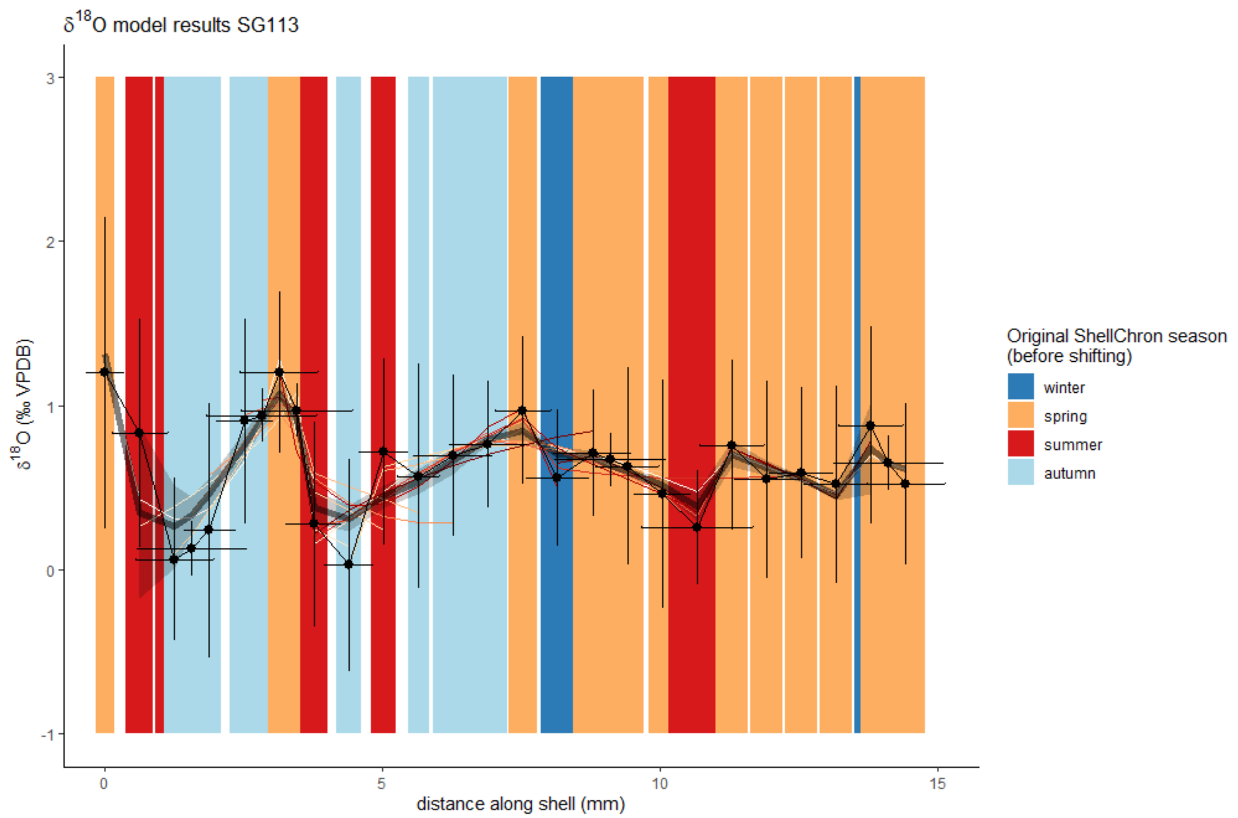

Fig. S22: **ShellChron oxygen isotope-based age modelling result for *Ostrea edulis* specimen SG113.** Colors and symbols as in **Fig. S20**.

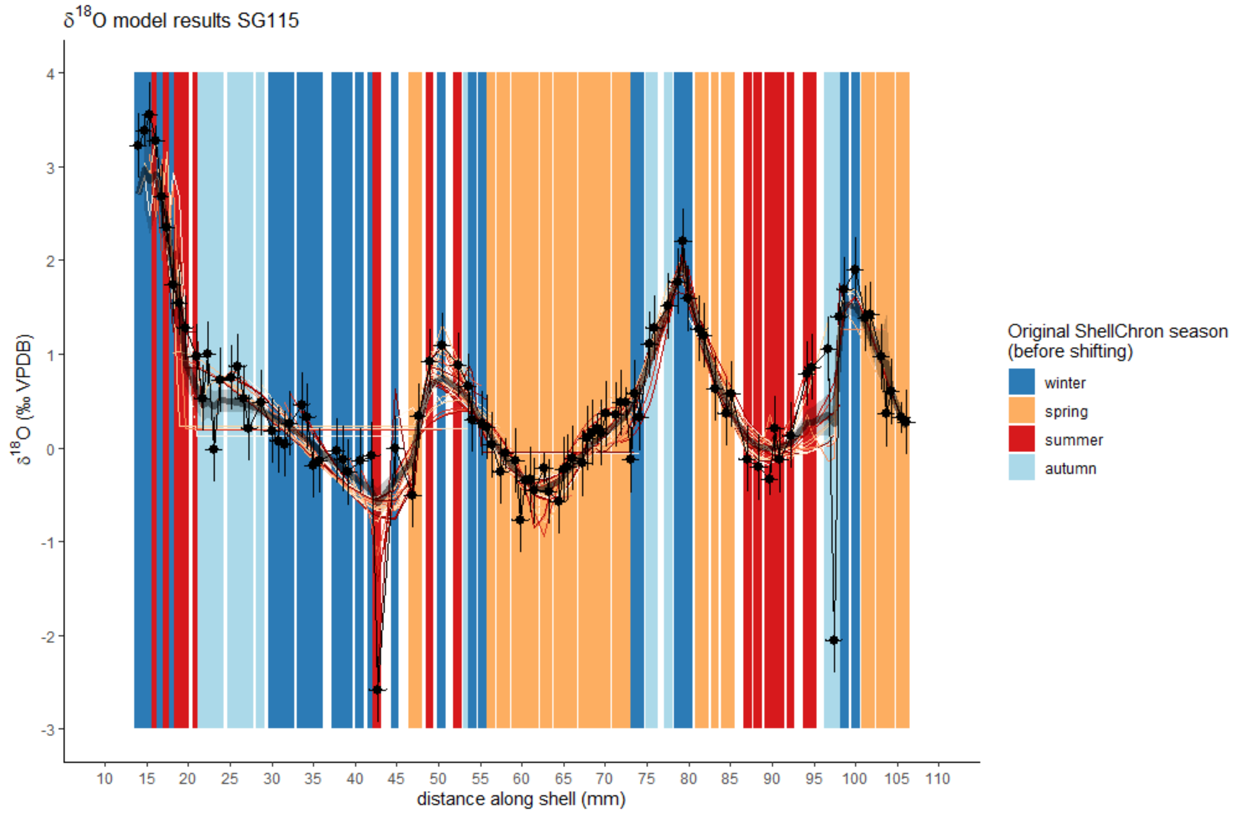

Fig. S23: **ShellChron oxygen isotope-based age modelling result for *Pecten complanatus* specimen SG115.** Colors and symbols as in **Fig. S20**. Note that the outliers at ~43 mm and ~97 mm did not affect the age assignment.

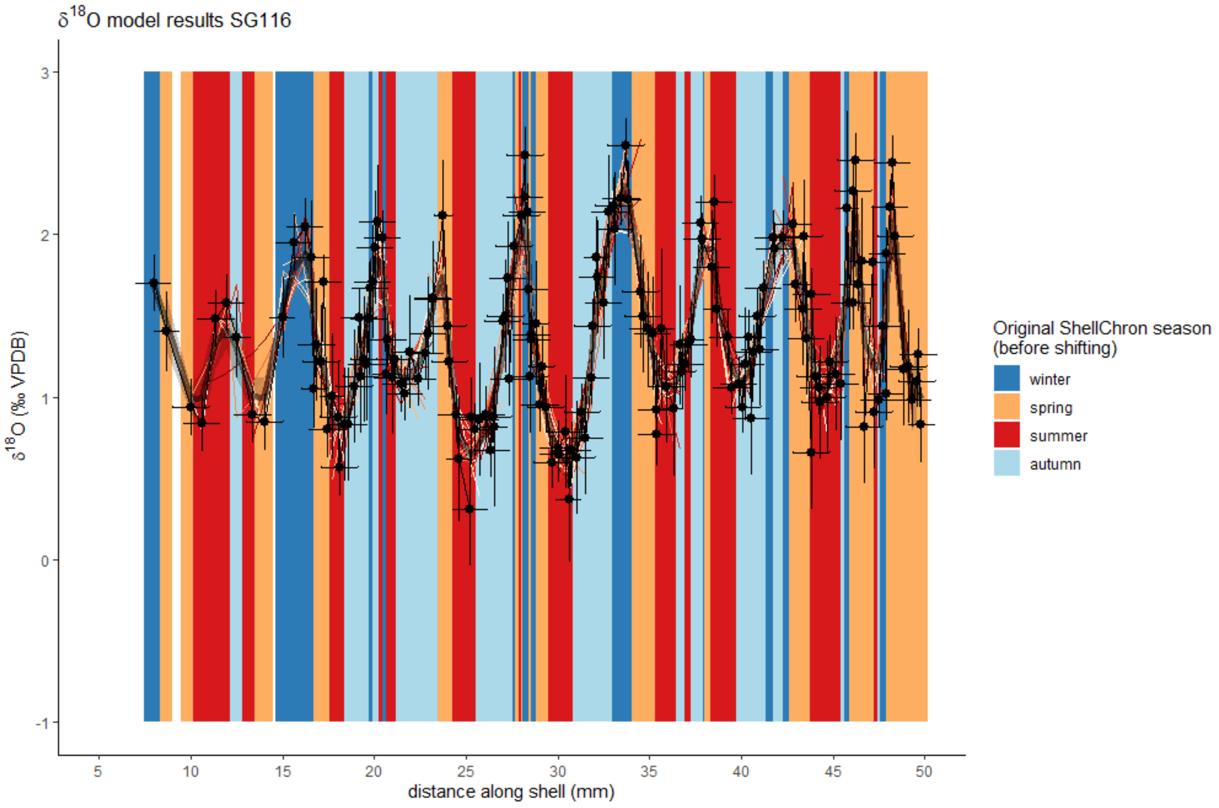

Fig. S24: **ShellChron oxygen isotope-based age modelling result for *Glycymeris radiolyrata* specimen SG116.** Colors and symbols as in **Fig. S20**.

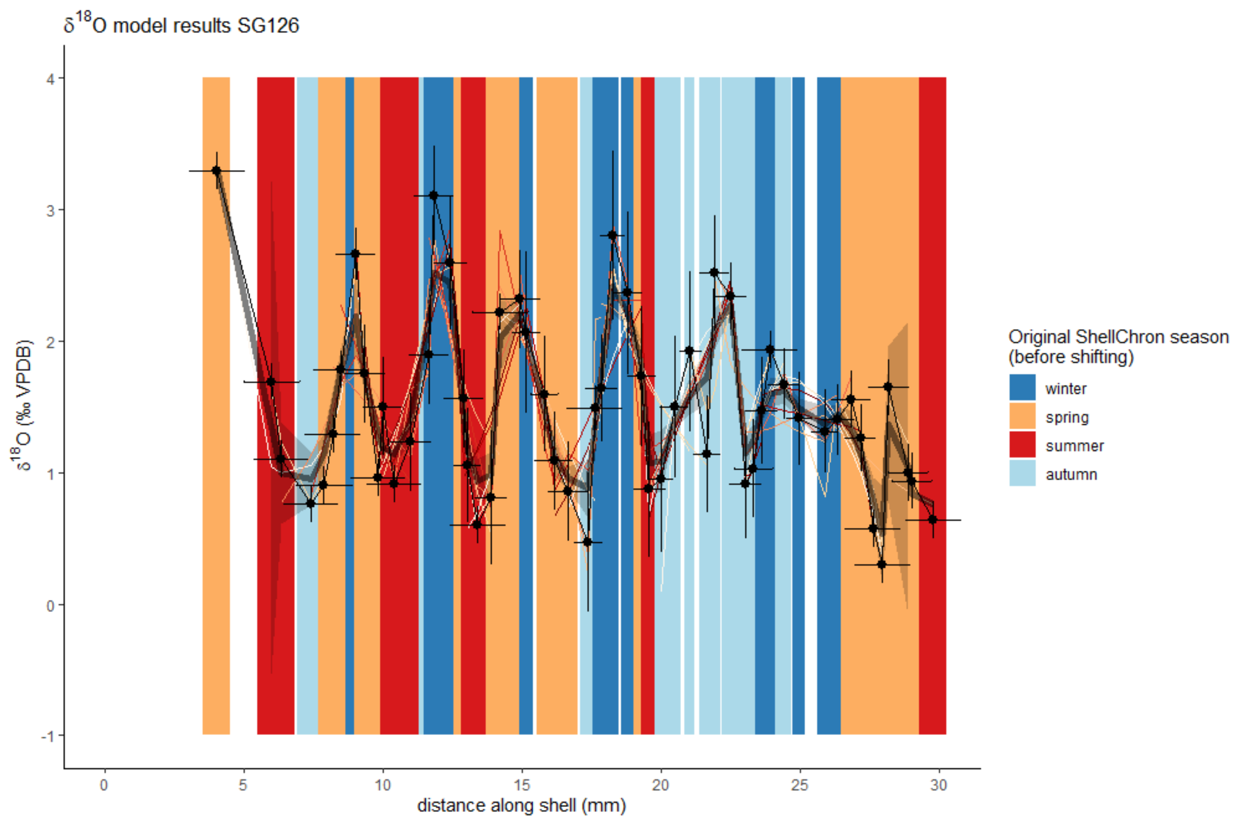

Fig. S25: **ShellChron oxygen isotope-based age modelling result for *Angulus benedeni benedeni* specimen SG126.** Colors and symbols as in **Fig. S20**.

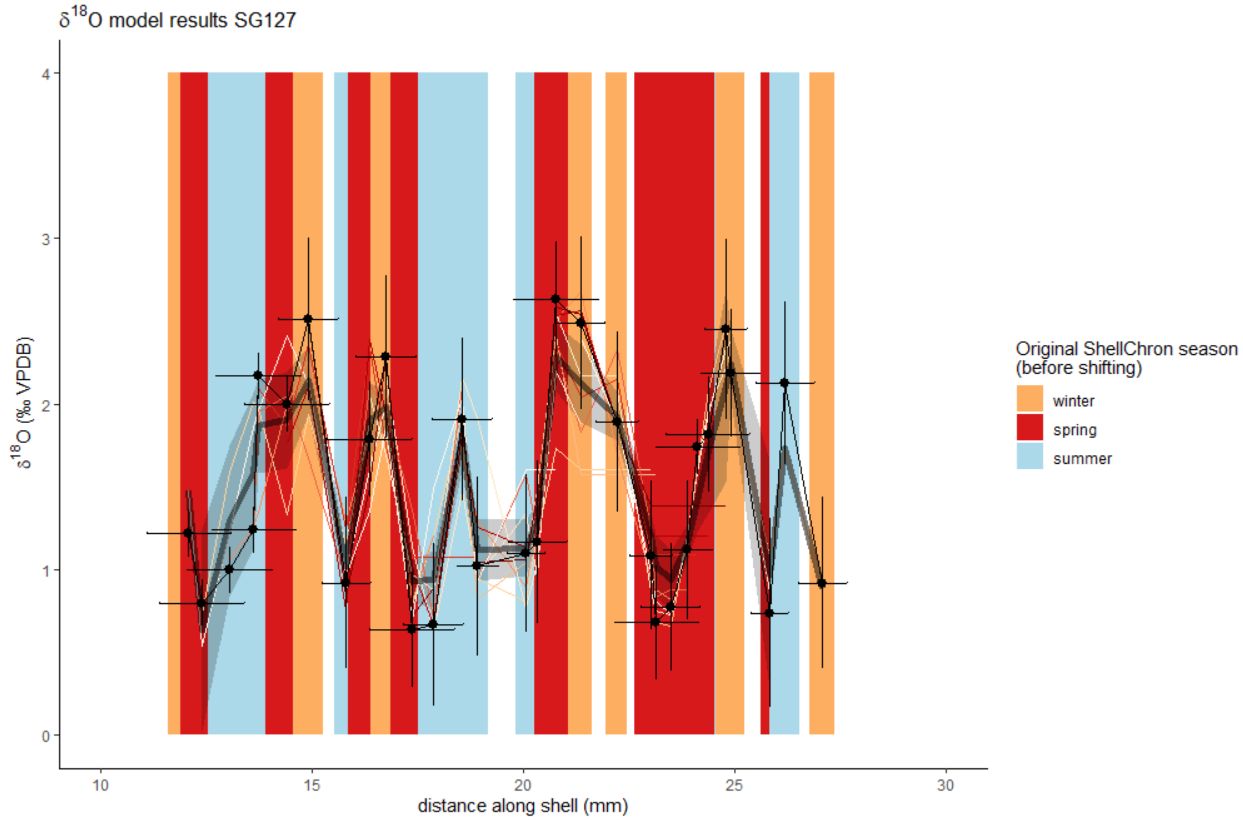

Fig. S26: **ShellChron oxygen isotope-based age modelling result for *Angulus benedeni benedeni* specimen SG127.** Colors and symbols as in **Fig. S20**.

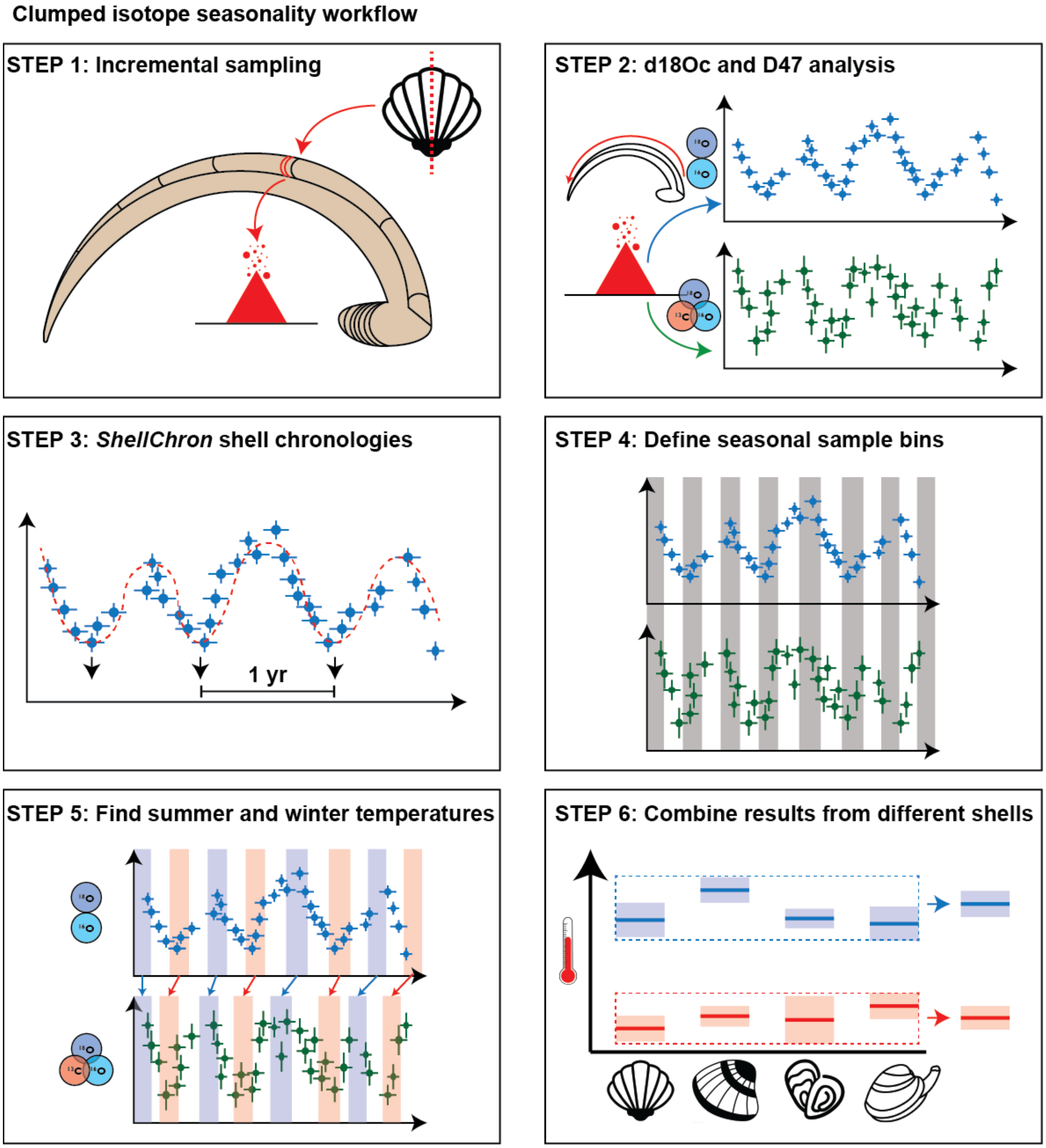

Fig. S27: **Schematic representation of the steps taken to group data relative to the four seasons, with summer and winter season defined as the coldest and warmest three-month periods.** Step 1: Mollusk shells were sampled incrementally to produce sub-seasonal scale carbonate powder samples (60-160 µg). Step 2: Carbonate powders were analyzed to produce records of carbon, oxygen and clumped isotopic composition. Step 3: The ShellChron(*75*) algorithm was used to create age models for each specimen based on seasonal patterns in oxygen isotope composition. Step 4: Samples were assigned to three-monthly sample bins. Step 5: The position of these three-monthly bins was moved along the newly defined time axis until the optimal warmest clumped isotope-based summer and winter temperatures were

found, establishing the lag between temperature seasonality and oxygen isotope seasonality (see section 3.4). Step 6: Means and confidence levels of three-monthly sample bins were calculated from the variability within groups of measurements, and weighted averages of summer and winter temperatures were aggregated. In this procedure, samples were weighted using the inverse of the variance associated with the uncertainty in the sample bin (see below).

3.4 Binning seasonal data and calculating seasonal temperature averages and uncertainties

Based on the age models, results were grouped in three-monthly bins per specimen, one bin for each of the four seasons (“winter”, “spring”, “summer” and “autumn”). Note that, through this procedure, results from the same season but deposited during different growth years are grouped in the same time bin (see step 5 in the online supplement).

The disadvantage of this grouping procedure is that it assumes that the phase of the seasonal $\delta^{18}O_c$ cycle is equal to that of the temperature seasonality. This assumption does not hold in each case (see discussion in (*113*)). To consider the effect of a potential out-of-phase seasonal variability in the $\delta^{18}O$ composition of the seawater ($\delta^{18}O_w$) on the seasonal $\delta^{18}O_c$ cycle, this assignment of samples to three-monthly bins based on shell chronologies was repeated 90 times. During each iteration, the boundaries between the four seasonal time bins were shifted by one day, thus shifting the seasons around the annual cycle (see step 7 in the online supplement). Note that, during this exercise of shifting the seasons, the number of days within each season, according to the age model determined by ShellChron, remains constant, but the number of measurements per season does not. This can happen because, due to a shift in the boundaries between the seasonal bins by one day, one or more datapoints can shift from one season to the next, changing the division of measurements over the four seasons while the total number of measurements in a given specimen remains the same.

For each iteration mean temperatures and their uncertainties (expressed as 95% confidence levels) were reconstructed for all seasons. Uncertainty on the clumped isotope temperature calibration was propagated into the uncertainty of paleotemperature reconstructions by applying a Monte Carlo simulation on paired slope and intercept values sampled from the uncertainty distribution around the calibration parameters reported by (*77*), taking into account the covariance between slope and intercept. A Student’s t-test was applied to find the seasonal configuration that resulted in the most significant temperature difference between the warmest and coldest non-adjacent seasons. In this configuration, the warmest temperature bin was defined as the summer season and the coldest non-adjactent temperature bin as the winter. The bins of samples in between the summer and winter were defined as spring and autumn.

Note that this binning approach results in large variations in sample size between seasonal bins within and between specimens. As a consequence, some seasonal estimates (e.g. the summer temperature of *Angulus benedeni benedeni* specimen SG127; see **Table S3**) are considerably more uncertain than estimates for other specimens and other seasons. In addition, variability in the number of measurements within seasonal bins during this approach may result in the situation where the optimal binning solution (according to the Student’s T-test) is not the solution in which the difference between mean summer and winter clumped isotope value is highest. This can occur because the uncertainty on the summer and winter average may be higher in this maximum-difference scenario because the sample size within the summer and winter bins is smaller. This causes the result of the T-test to be less significant (higher p-

value) than that of a scenario where summer and winter means are more similar, but with a higher sample size per seasonal bin.

After experimenting with the method for binning the seasons in each specimen, we concluded that low sample sizes could not be avoided in all cases while keeping the binning strategy uniform between specimens (which is necessary for the seasonal estimates to be comparable). The weighting of seasonal means while averaging between specimens (see below) ensured that these more uncertain bins do not bias the average seasonal temperatures. Some seasonal bins, such as the spring and autumn of *Angulus benedni benedeni* specimen SG127 and the autumn bin of *Ostrea edulis* contained too few datapoints (< 5) to calculate a reliable mean and uncertainty range. Hence, these seasons were excluded from further analysis.

Finally, the weighted mean temperature and uncertainty for each of the four seasons was calculated by combining the means and standard deviations of temperature reconstructions of the same season from the individual shells. To calculate mean summer and winter sea surface temperature (SST) based on all specimens, an average was calculated from the three-monthly summer and winter averages of the specimens weighted by the inverse of the variance of uncertainty on the reconstruction. Uncertainties on this pooled average summer and winter reconstruction were determined by propagating the uncertainty on the summer and winter data per specimen through a weighted standard deviation, following the statistical routine worked out in (*34*). This procedure considers both the variability within the seasonal temperature bins and the variability between the temperature bins from the same season but from different specimens (see step 8 in the online supplement). A schematic overview of the full sampling and data processing workflow is provided in **Fig. S27**. An overview of median seasonal temperature results for all specimens and their uncertainties is provided in **Table S3** and plots of all clumped data arranged by age in the specimen and colored by their final seasonal bin are provided in **S28-S34**.

Specimen	Species	Season	N	Temperature (°C)	Minimum temperature (°C; 95% CL)	Maximum temperature (°C; 95% CL)
SG105	*Pygocardia rustica*	autumn	43	10.0	8.4	11.3
SG105	*Pygocardia rustica*	spring	37	13.1	10.8	14.6
SG105	*Pygocardia rustica*	winter	9	6.3	2.8	10.4
SG105	*Pygocardia rustica*	summer	6	21.4	19.2	26.9
SG107	*Arctica islandica*	spring	8	13.9	12.5	15.8
SG107	*Arctica islandica*	autumn	28	11.0	9.7	13.6
SG107	*Arctica islandica*	summer	10	22.0	19.2	23.8
SG107	*Arctica islandica*	winter	7	7.5	6.2	8.8
SG113	*Ostrea edulis*	winter	27	9.5	7.1	21.9
SG113	*Ostrea edulis*	summer	13	21.6	18.5	24.5
SG113	*Ostrea edulis*	autumn	29	11.6	8.0	14.0
SG115	*Pecten complanatus*	summer	30	19.3	18.7	22.4
SG115	*Pecten complanatus*	autumn	30	16.2	14.0	22.4
SG115	*Pecten complanatus*	spring	16	17.8	15.3	23.7
SG115	*Pecten complanatus*	winter	8	12.3	6.9	15.6
SG116	*Glycymeris radiolyrata*	spring	24	16.8	11.0	19.5
SG116	*Glycymeris radiolyrata*	summer	29	17.9	14.9	20.8
SG116	*Glycymeris radiolyrata*	winter	37	10.4	7.6	13.3
SG116	*Glycymeris radiolyrata*	autumn	41	15.1	12.9	19.4
SG126	*Angulus benedeni benedeni*	autumn	18	5.8	2.6	8.6
SG126	*Angulus benedeni benedeni*	spring	18	7.3	5.7	9.4
SG126	*Angulus benedeni benedeni*	summer	9	10.6	9.8	15.4
SG126	*Angulus benedeni benedeni*	winter	22	1.9	-0.2	9.7
SG127	*Angulus benedeni benedeni*	summer	13	15.5	12.5	21.0
SG127	*Angulus benedeni benedeni*	winter	28	8.8	7.9	10.8

Table S3: **Median and 95% confidence level boundaries of seasonal temperature reconstructions from each specimen.**

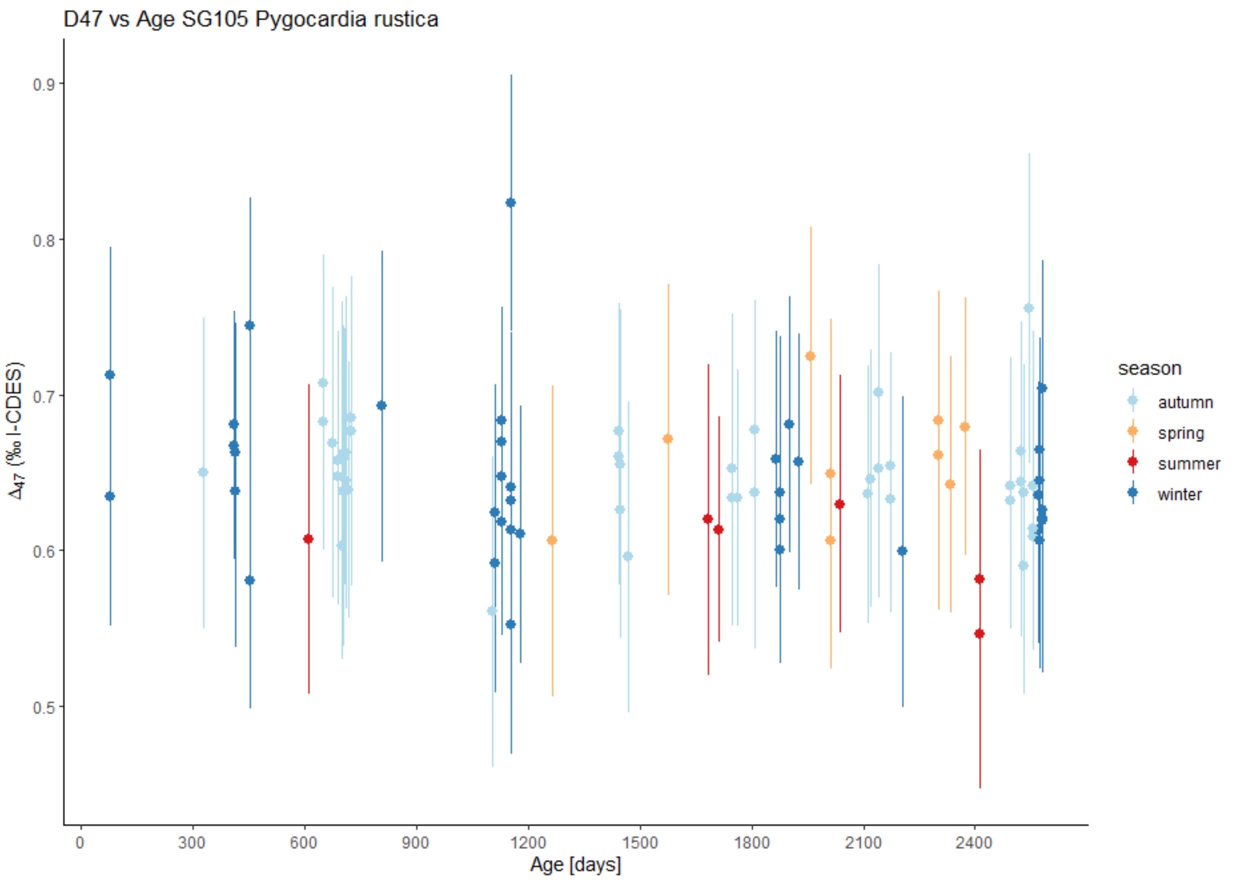

Figure S28: **Overview of Δ_{47} values of individual measurements in specimen SG105 plotted against age according to the ShellChron age model.** The colors represent seasonal bins to which the measurements were assigned after shifting the seasonal binning (see explanation above).

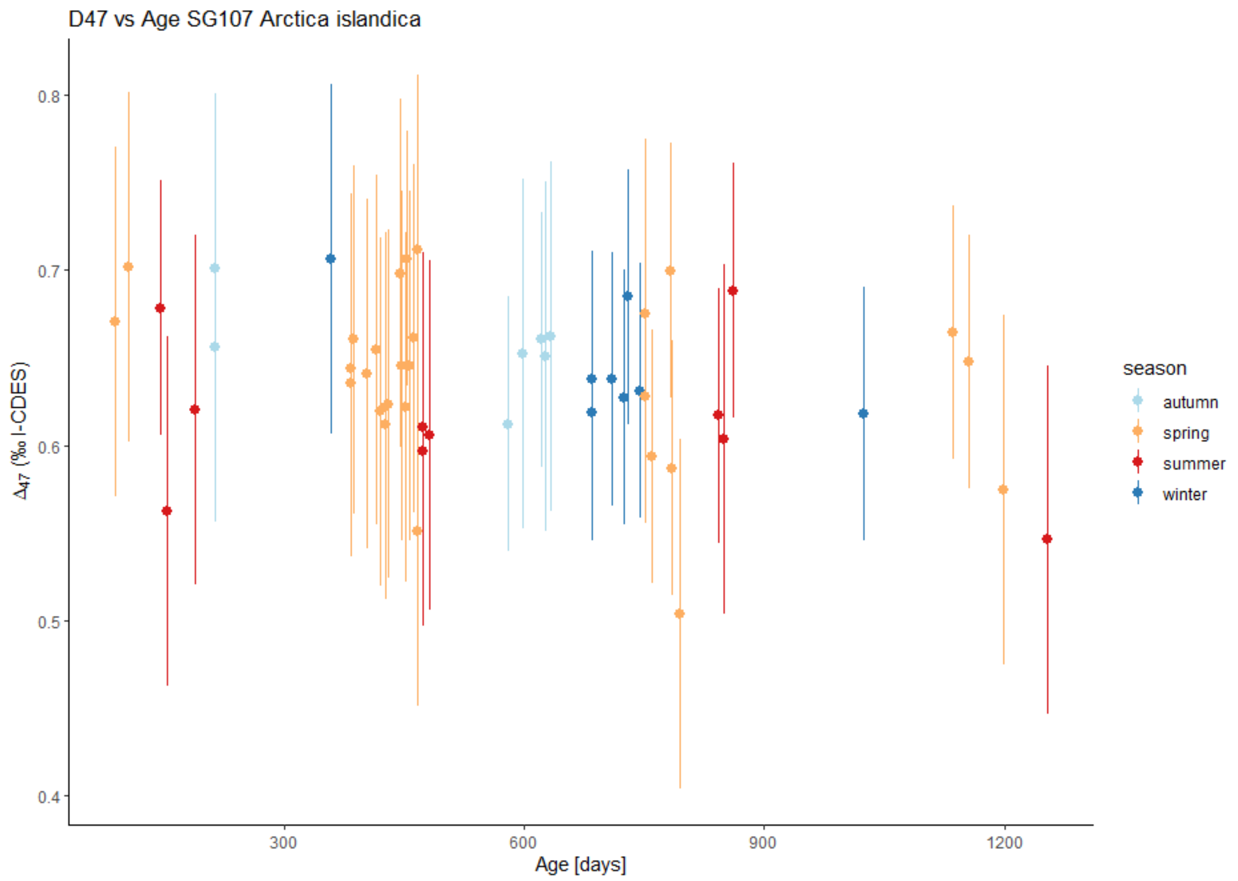

Figure S29: **Overview of Δ_{47} values of individual measurements in specimen SG107 plotted against age according to the ShellChron age model.** The colors represent seasonal bins to which the measurements were assigned after shifting the seasonal binning (see explanation above).

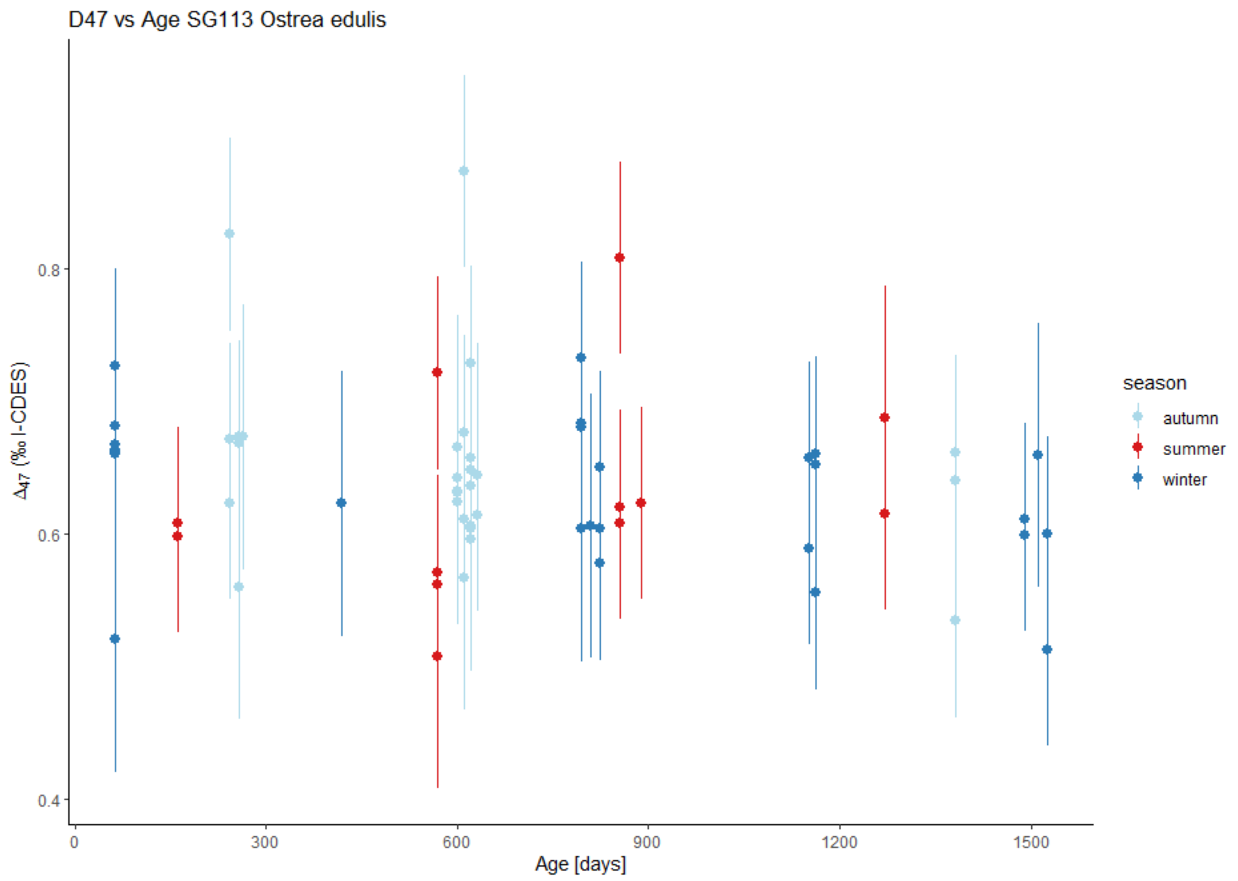

Figure S30: **Overview of Δ_{47} values of individual measurements in specimen SG113 plotted against age according to the ShellChron age model.** The colors represent seasonal bins to which the measurements were assigned after shifting the seasonal binning (see explanation above). Note that values assigned to the spring bin are omitted from the analyses.

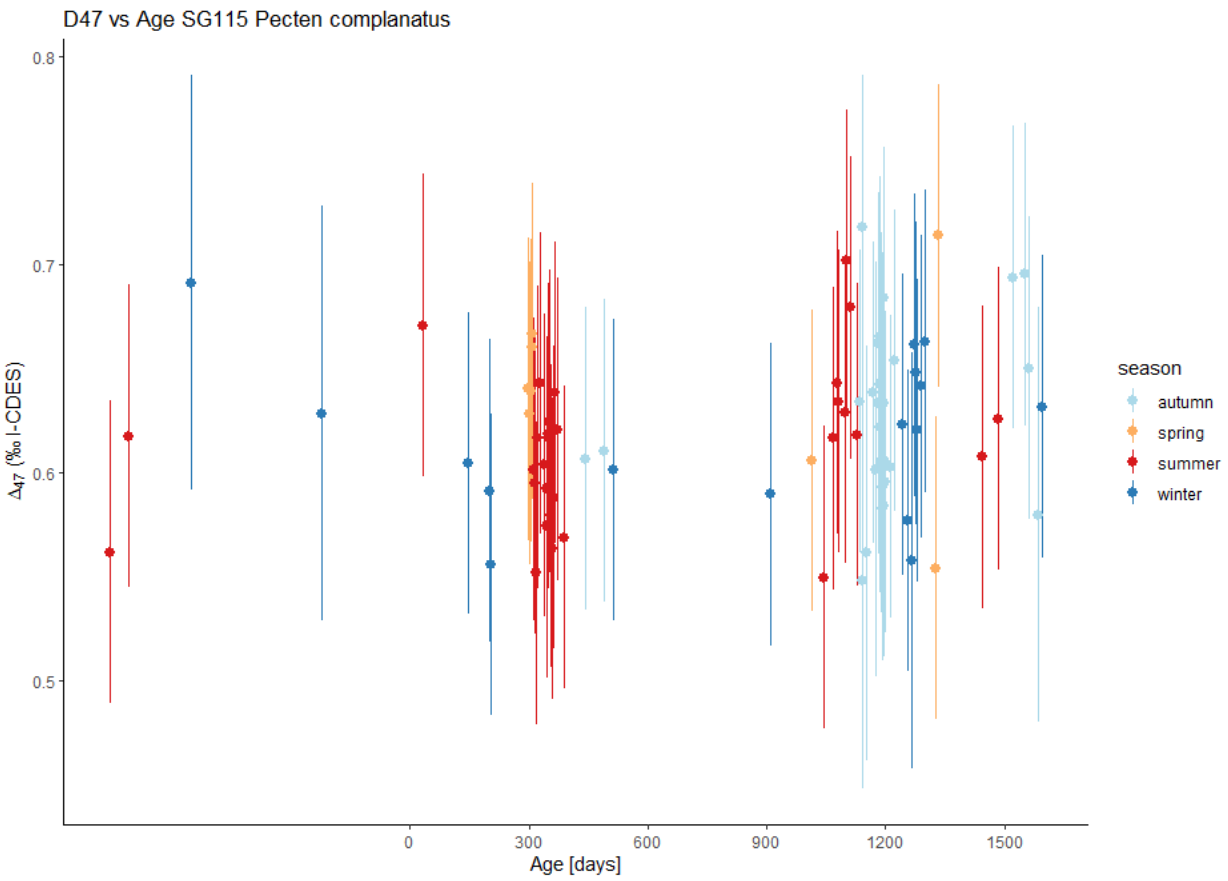

Figure S31: **Overview of Δ_{47} values of individual measurements in specimen SG115 plotted against age according to the ShellChron age model.** The colors represent seasonal bins to which the measurements were assigned after shifting the seasonal binning (see explanation above).

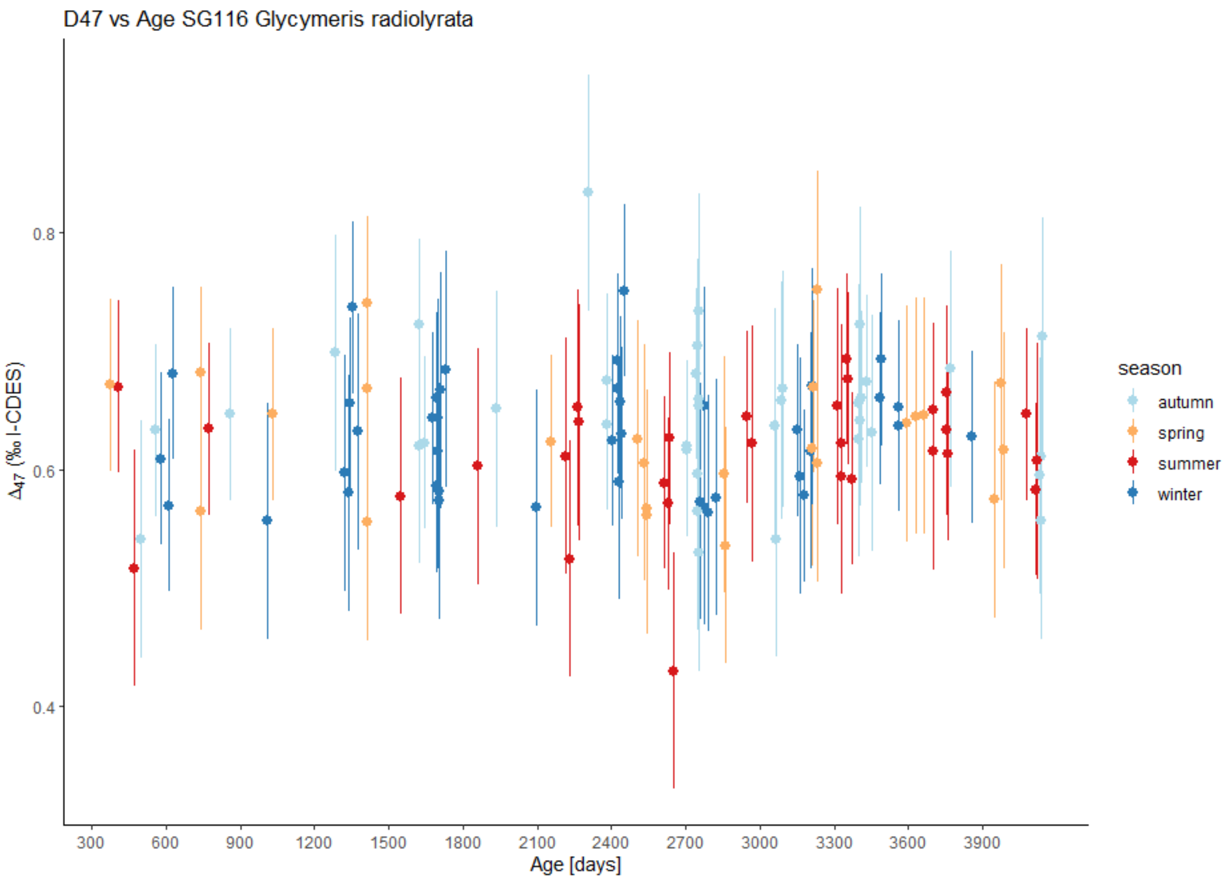

Figure S32: **Overview of Δ_{47} values of individual measurements in specimen SG116 plotted against age according to the ShellChron age model.** The colors represent seasonal bins to which the measurements were assigned after shifting the seasonal binning (see explanation above).

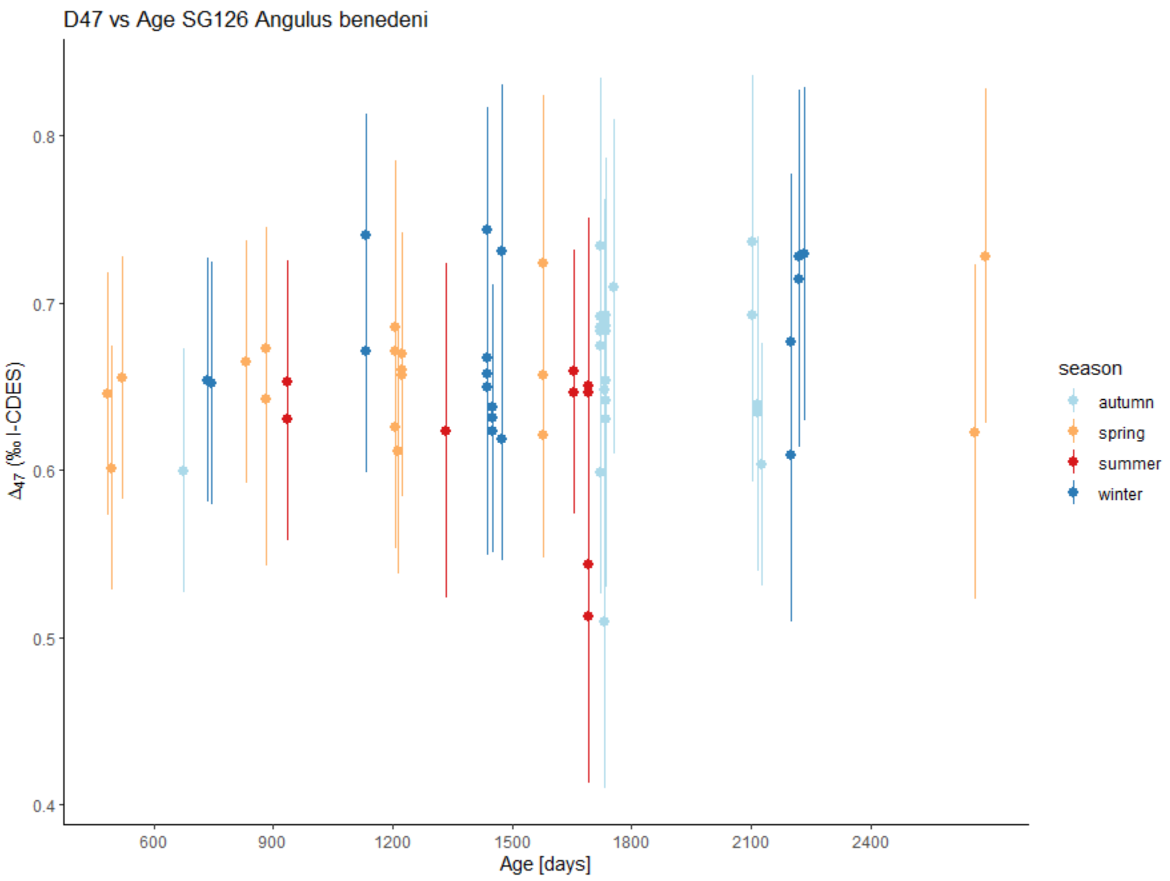

Figure S33: **Overview of Δ_{47} values of individual measurements in specimen SG126 plotted against age according to the ShellChron age model.** The colors represent seasonal bins to which the measurements were assigned after shifting the seasonal binning (see explanation above).

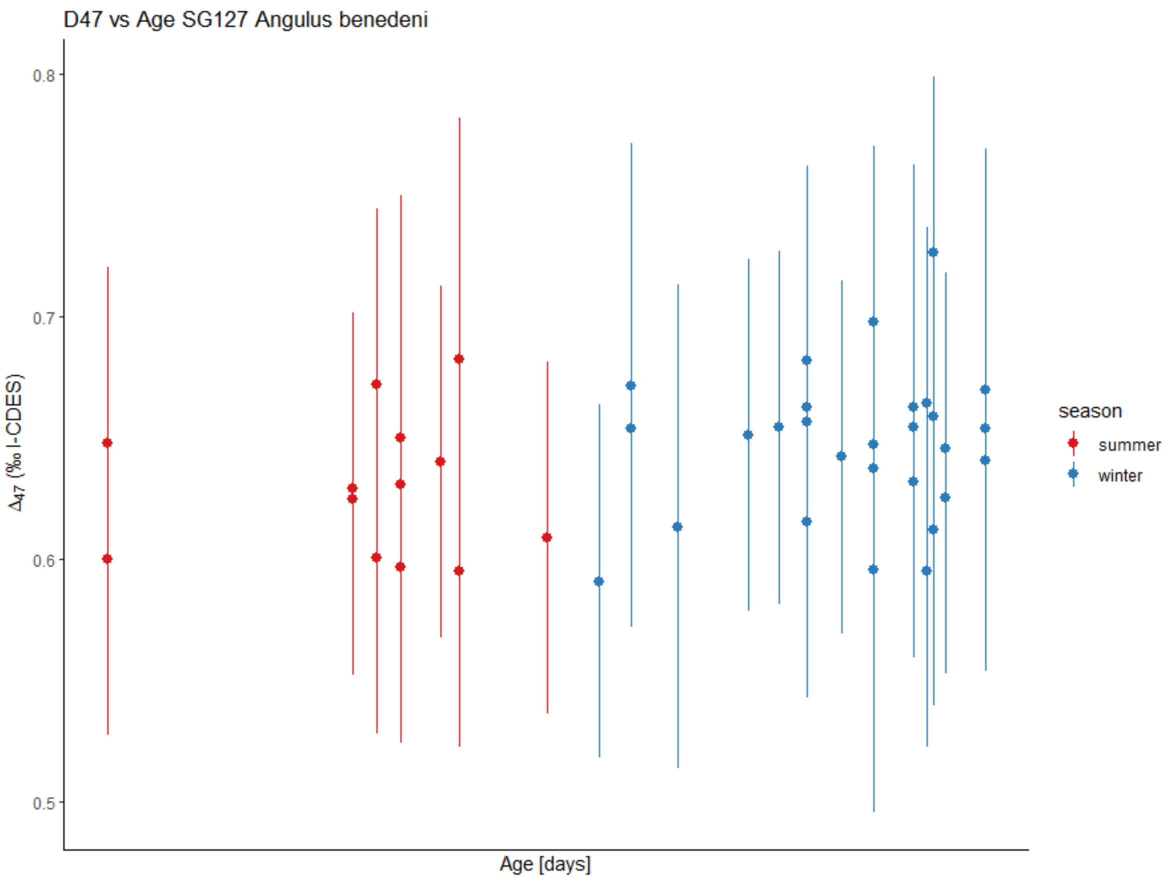

Figure S34: **Overview of Δ_{47} values of individual measurements in specimen SG127 plotted against age according to the ShellChron age model.** The colors represent seasonal bins to which the measurements were assigned after shifting the seasonal binning (see explanation above). Note that values belonging to the spring and autumn bin are omitted.

4. PlioMIP2 model output

4.1 The Pliocene Model Intercomparison Project

In order to compare our reconstructions with model results from the Pliocene Model Intercomparison Project 2(*40*), maps of SST outcomes of nine General Circulation Models (GCMs) were obtained from the PlioMIP2 consortium (see (*40*) and references within, and contributions from individual model results in the PlioMIP2 special issue in Climate of the Past: https://cp.copernicus.org/articles/special_issue642.html). For the temperature comparison in this study, outcomes of the models CCSM4-UoT (CESM 1.0.5; University of Toronto, CA(*114*)), CCSM4-Utr (CESM 1.0.5; Institute for Marine and Atmospheric Research Utrecht, NL(*115*)), COSMOS (COSMOS-landveg r2413; Alfred Wegener Institute, DE(*116*)), EC-Earth3.3 (Stockholm University, SW(*117*)), GISS2.1G (Goddard Institute for Space Studies, USA(*118*)), HadCM3 (University of Leeds, UK(*119*)), IPSLCM6A (Laboratoire des Sciences du Climat et de l'Environment, FR(*120*)), MIROC4m (University of Tokyo, National Institute for Environmental Studies, Frontier Research Center for Global Change, JAMSTEC, JP(*121*)) and NorESM1-F (NORCE Norwegian Research Centre, Bjerknes Centre for Climate Research, Bergen, NO(*122*)) were used. In **Figures S43-S44** and in the main text, these nine models were used exclusively to produce the multi-model mean, while **Figures S35-S40** show the multi-model mean for the full PlioMIP model ensemble. For all models, results for two types of simulations were used: $Plio_{Core}$ runs with Pliocene conditions (atmospheric CO_2 concentrations set at 400 ppmV) and PI_{Ctrl} runs with pre-industrial conditions (atmospheric CO_2 concentrations set at 280 ppmV). For Pliocene runs, boundary conditions (e.g. land-sea mask and bathymetry of the oceans) were applied based on the PRISM4 mPWP reconstructions(*82*). PlioMIP2 models used in this study were run for between 500 and 4000 years (see details in supplementary information to (*40*)), and SST values for the resulting state of the modelled climate were used for the data-model comparison. Full details on the experimental design of PlioMIP2 are provided in (*40*) and in the papers describing the contributions of individual models cited above. NetCDF files containing SST values for all sea grid cells in all models are provided in the online supplement ("E280_SST" and "EOI_400_SST" in "PlioMIP_export").

4.2 Processing PlioMIP2 results to enable comparison with reconstructions

After identifying the latitude and longitude values and parameter names in the netCDF files, we extracted regional SST data from each model outcome for the southern North Sea Basin (51-55°N, 2-4°E) and summarized this data per month. For each month, a regional SST map was plotted per model outcome. These plots can be found in the online supplement ("PI_SST_monthplots" and "PWP_SST_monthplots" in "9_PWP_process_NetCDF"). Regional (southern North Sea Basin) monthly SST data for each model run (PI and PWP) are provided in the online supplement ("North_Sea_PI_SST_modeldata" and "North_Sea_PWP_SST_modeldata" in "9_PWP_process_NetCDF") and were used to plot PLIOMIP modelled mPWP seasonality in **Fig. 2b** of the main text. The script detailing the processing of the PLIOMIP data is provided in the online supplement (step 9). The monthly modelling results of North Sea SST were plotted together with the reconstruction data (see **section 3**) to produce **Fig. 1a & b** in the main text (step 10 in online supplement). To compare seasonal averages, the monthly data from the PLIOMIP models was aggregated into three-monthly SST averages, propagating uncertainties estimated from variability between the model results into a 95% confidence interval to allow direct comparison with the reconstruction data. In this procedure, winter is defined by the months January to March, spring as April to June, summer as July to September and autumn as October to December.

In addition to summarizing the multi-model mean sea surface temperatures at the sampling location, we also mapped seasonal contrast in SAT (**Figs. S35**-**S36**), SST (**Figs. S37-S39**), sea ice cover (**Fig. 40**), fraction of cloud cover (**Figs. S41-S42**) and prevailing wind patterns (**Figs. S43-S44**) for pre-industrial and mPWP model runs across the North Atlantic region. Note that cloud fraction data was only available for models HadCM3, IPSLCM6A and MIROC4m. These maps helped to visualize the structure of seasonality and warming under the mPWP scenario and formed the basis for the discussion of the effect of Polar Amplification on seasonal temperature variability during the mPWP (see **Discussion** in the main manuscript text).

In short, the figures below highlight a strong increase in seasonal SAT contrast over sea ice regions (**Fig. S35**), while SST seasonality is reduced in the high latitudes (**Fig. S37-S39**). This seasonal effect of mPWP-scale warming is related to a loss in winter sea ice over in several high-latitude regions such as the Labrador and Barentz Sea (**Fig. S40**). In the mid-latitudes, westerly wind speed is reduced in summer (**Fig. S43**) resulting in a reduction in summer cloud cover over Europe (**Fig. S41-S42**), which drives enhanced summer warming in this region under mPWP conditions. Finally, **Fig. S44** shows how the mollusc-based reconstructions compare with SNS SAT outcomes in the PlioMIP ensemble.

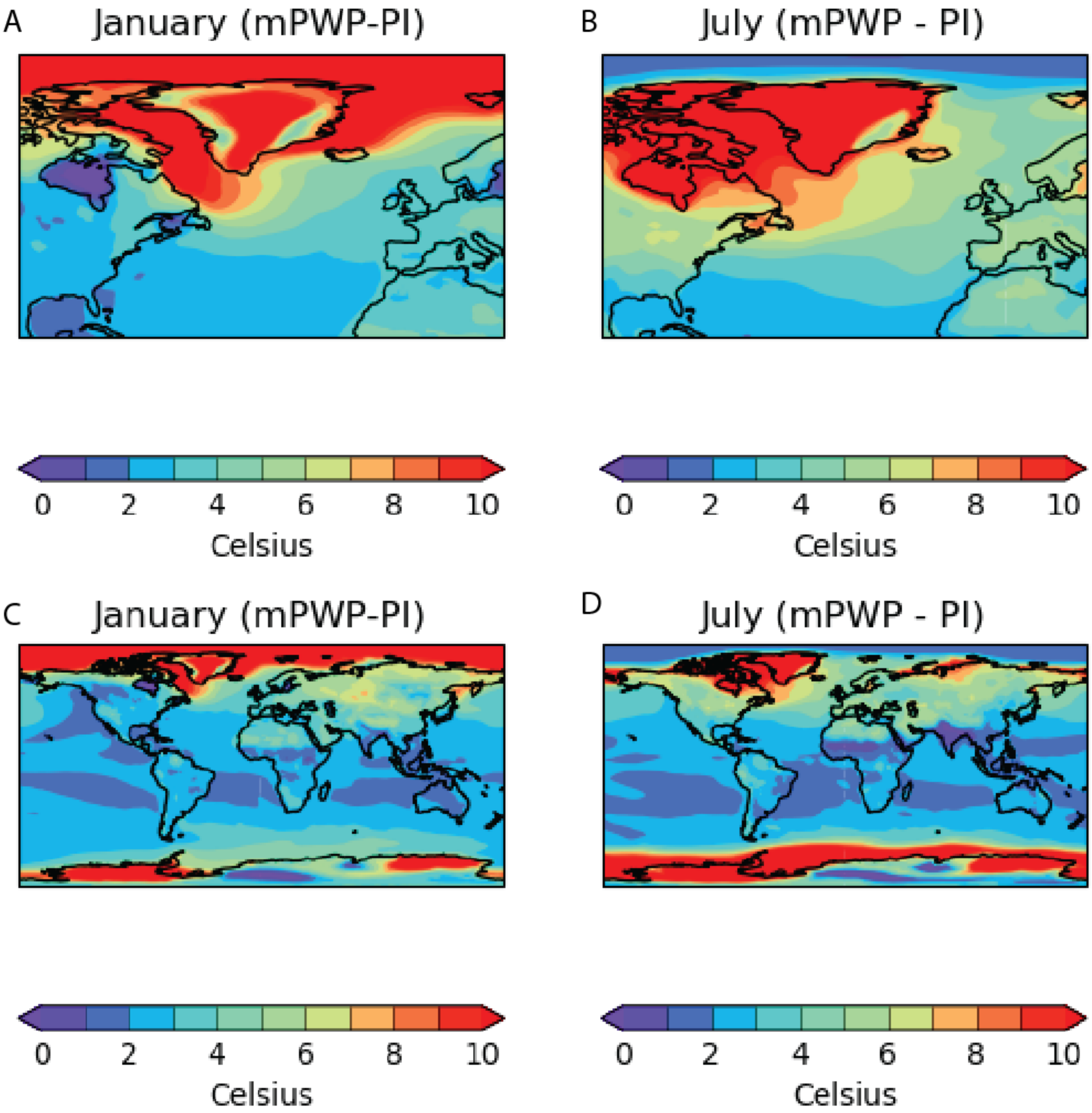

Figure S35: **Multi-model mean surface air temperature anomaly between pre-industrial and mPWP PlioMIP2 model simulations for January (A & C) and July (B & D).** Note that absolute winter and summer temperatures are always higher in mPWP runs than in in pre-industrial runs.

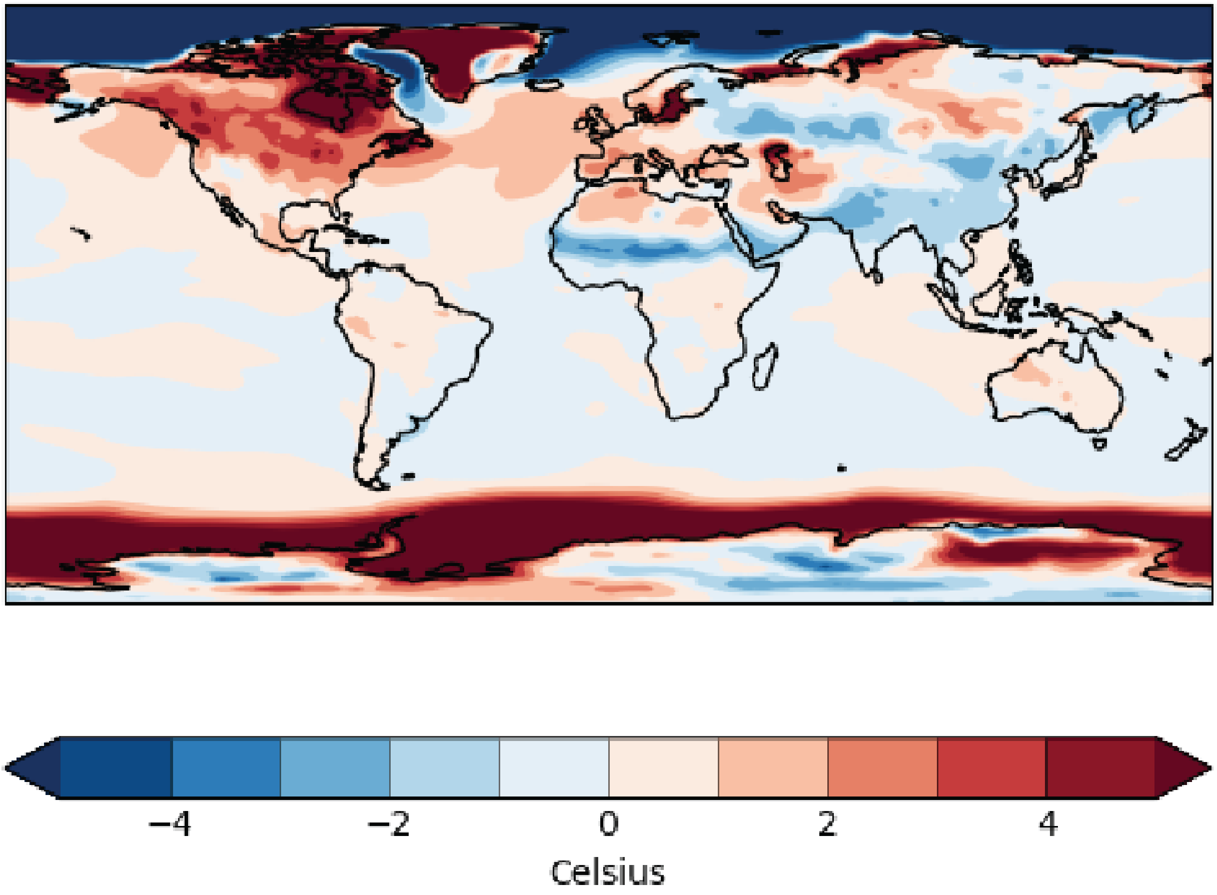

Figure S36: **Multi-model mean seasonal contrast in surface air temperature anomaly between pre-industrial and mPWP PlioMIP2 model simulations.** Positive values (red colors) indicate higher seasonal contrast in SAT in mPWP simulations than in pre-industrial simulations, while negative values (blue) indicate reduced seasonal SAT contrast in mPWP runs. Note that absolute winter and summer temperatures are always higher in mPWP runs than in in pre-industrial runs (**Fig. S28**), meaning that enhanced seasonal contrast indicates summer warming.

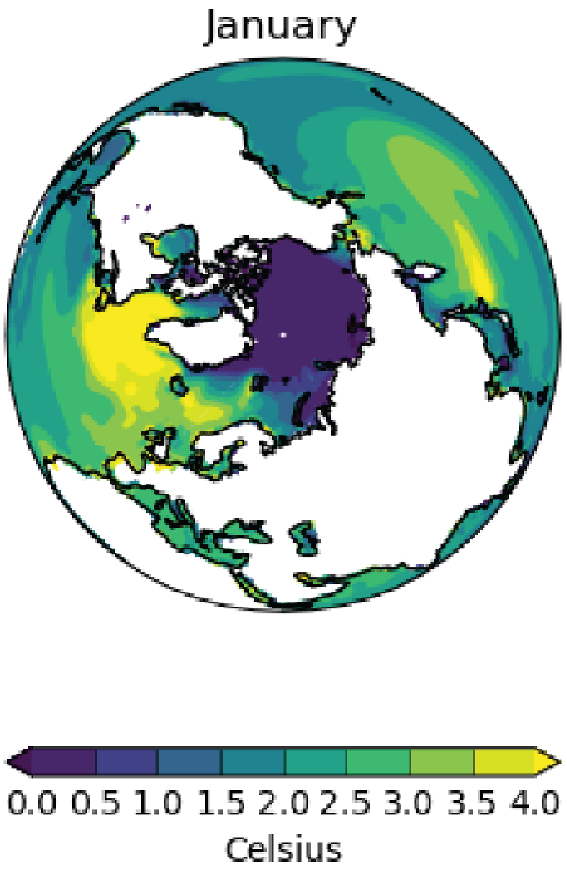

Figure S37: **Multi-model mean sea surface temperature anomaly in the northern hemisphere oceans between mPWP and PI simulations of PlioMIP2 models for January and July.**

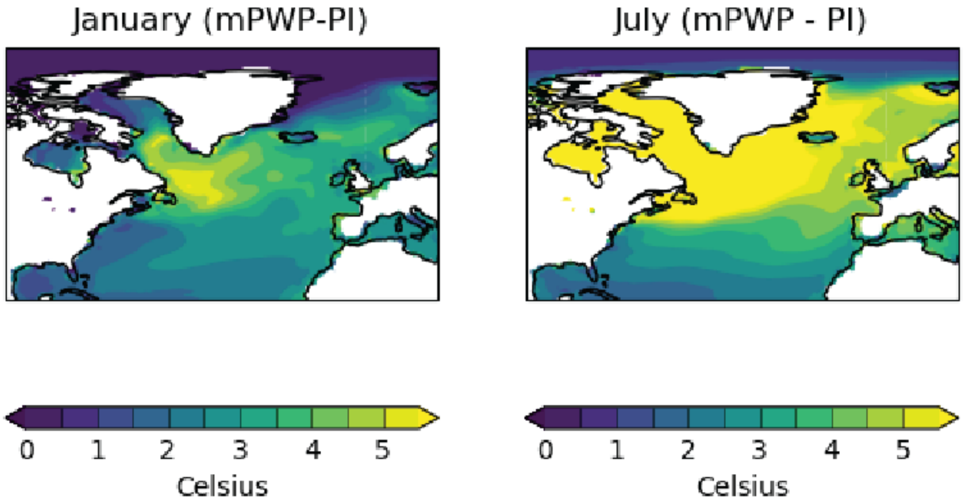

Figure S38: **Multi-model mean sea surface temperature anomaly in the North Atlantic between mPWP and PI simulations of PlioMIP2 models for January and July.**

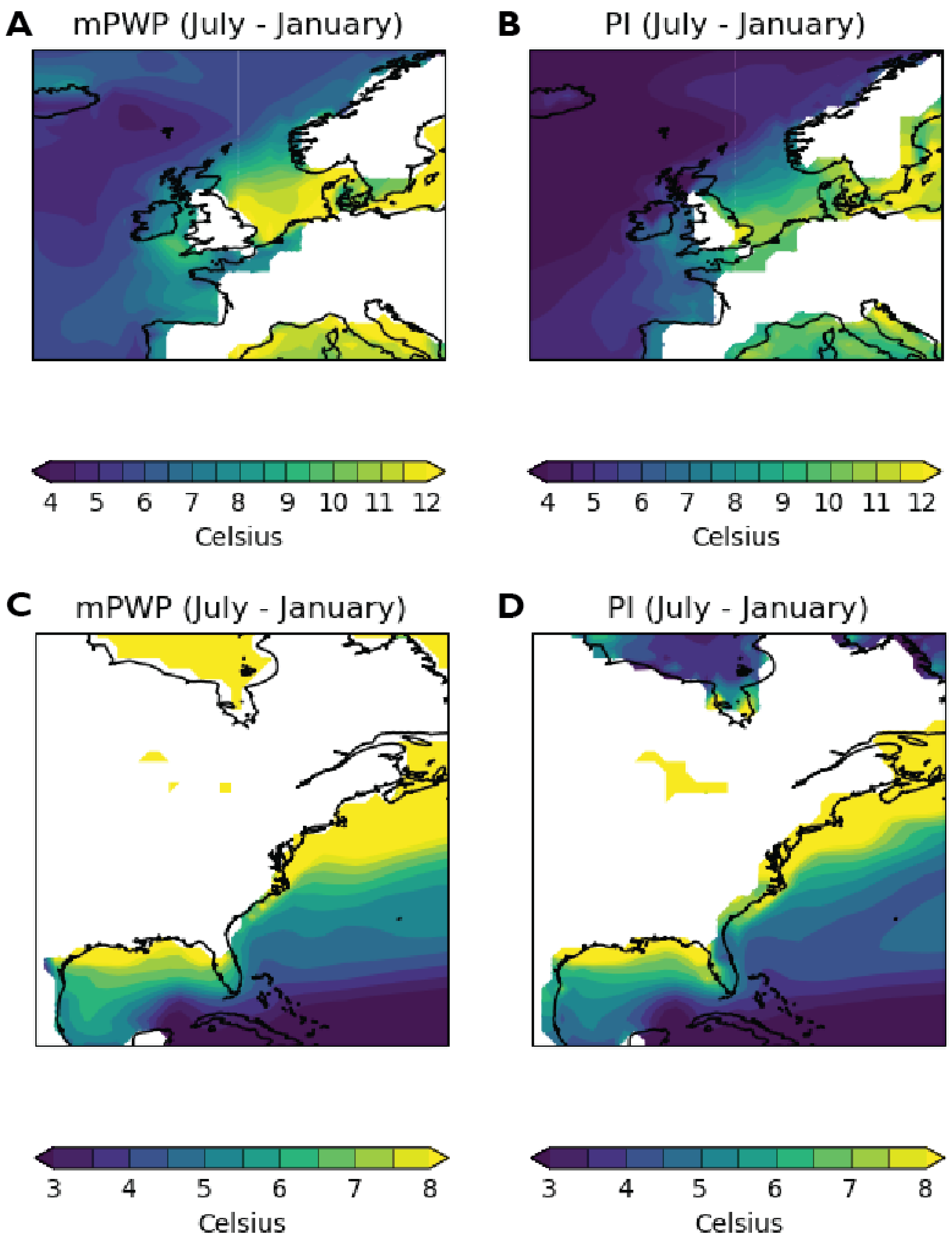

Figure S39: **Multi-model mean seasonal sea surface temperature contrast for mPWP (A & C) and pre-industrial (B & D) PlioMIP2 model simulations**, showing enhanced seasonal SST contrast in shelf seas in the mid-latitudes.

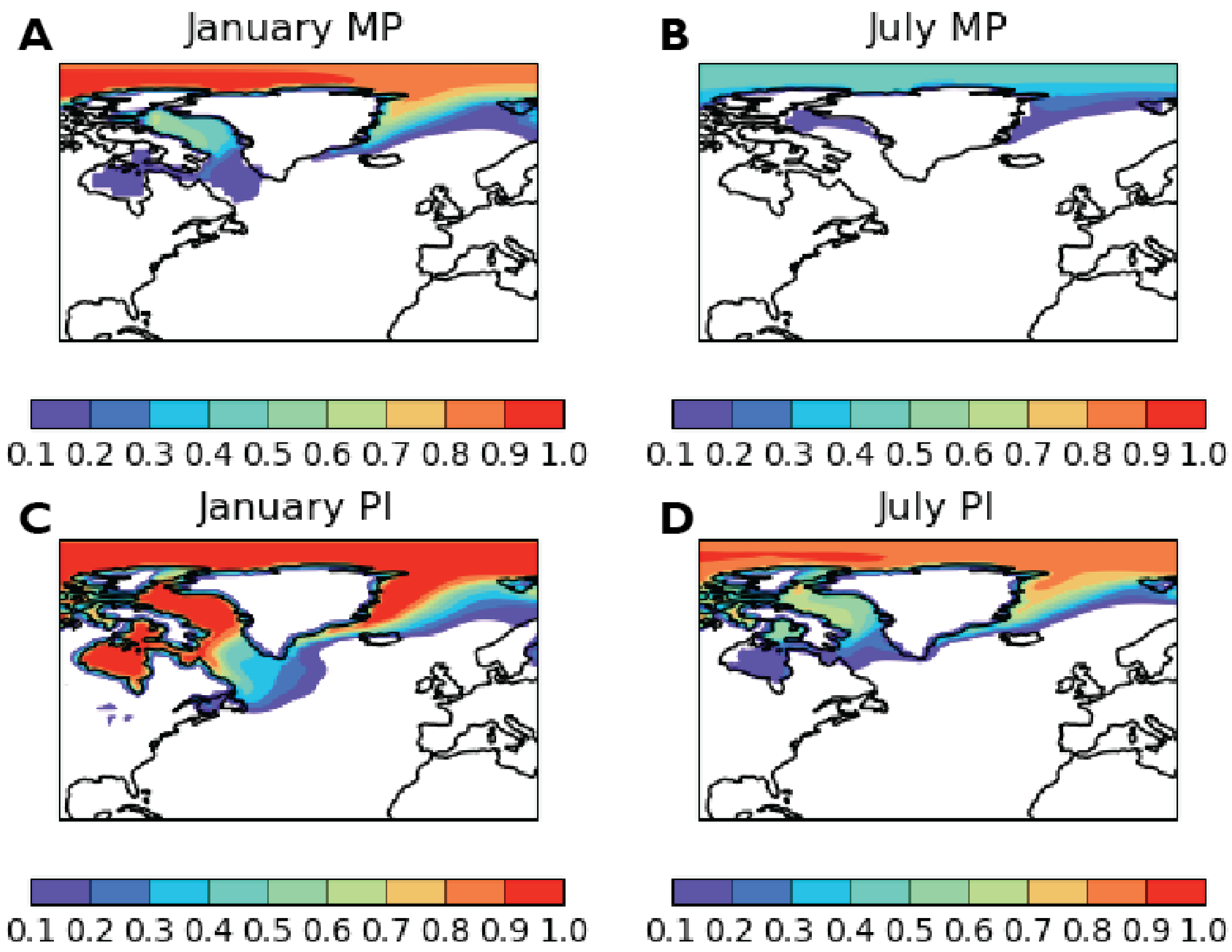

Figure S40: **Multi-model mean sea ice cover in the North Atlantic in mPWP PlioMIP2 simulations ("MP"; A January, B July) and pre-industrial PlioMIP2 simulations ("PI"; C January, D July).** Color scale indicates the mean fraction of the grid cell covered by sea ice: A value of 1.0 means all models simulate full sea ice cover in that location during that month, while a value of 0.0 indicates no sea ice cover is simulated in that location during the month.

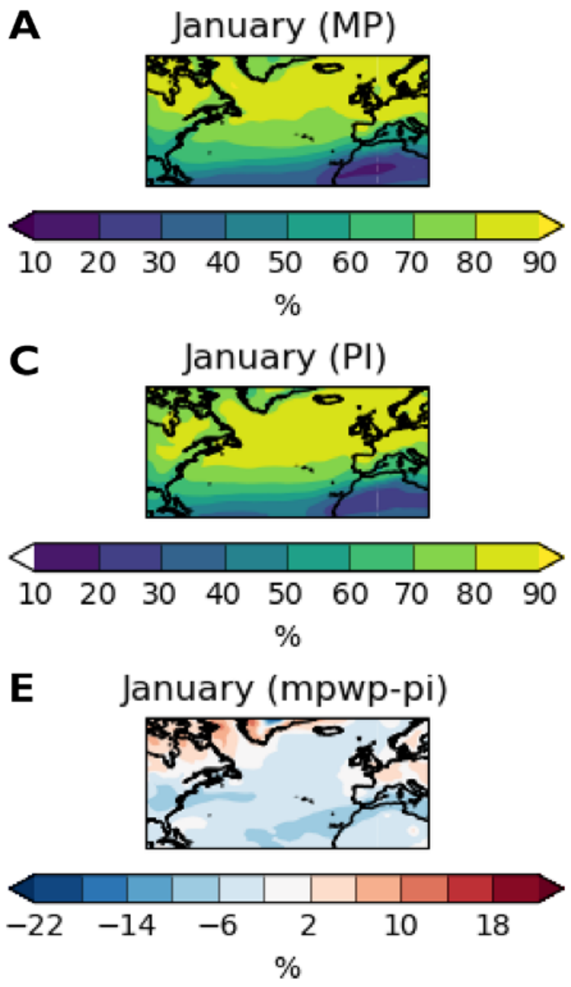

Figure S41: **Mean cloud cover fraction (in percentage) from models HadCM3, IPSLCM6A and MIROC4m over the North Atlantic** for mPWP PlioMIP2 simulations ("MP"; **A** & **B**), pre-industrial simulations ("PI"; **C** & **D**) and anomalies between mPWP and PI simulations (**E** & **F**). Model outcomes are compared for the coldest month (January; **A**, **C** & **E**) and the warmest month (July; **B**, **D** & **F**).

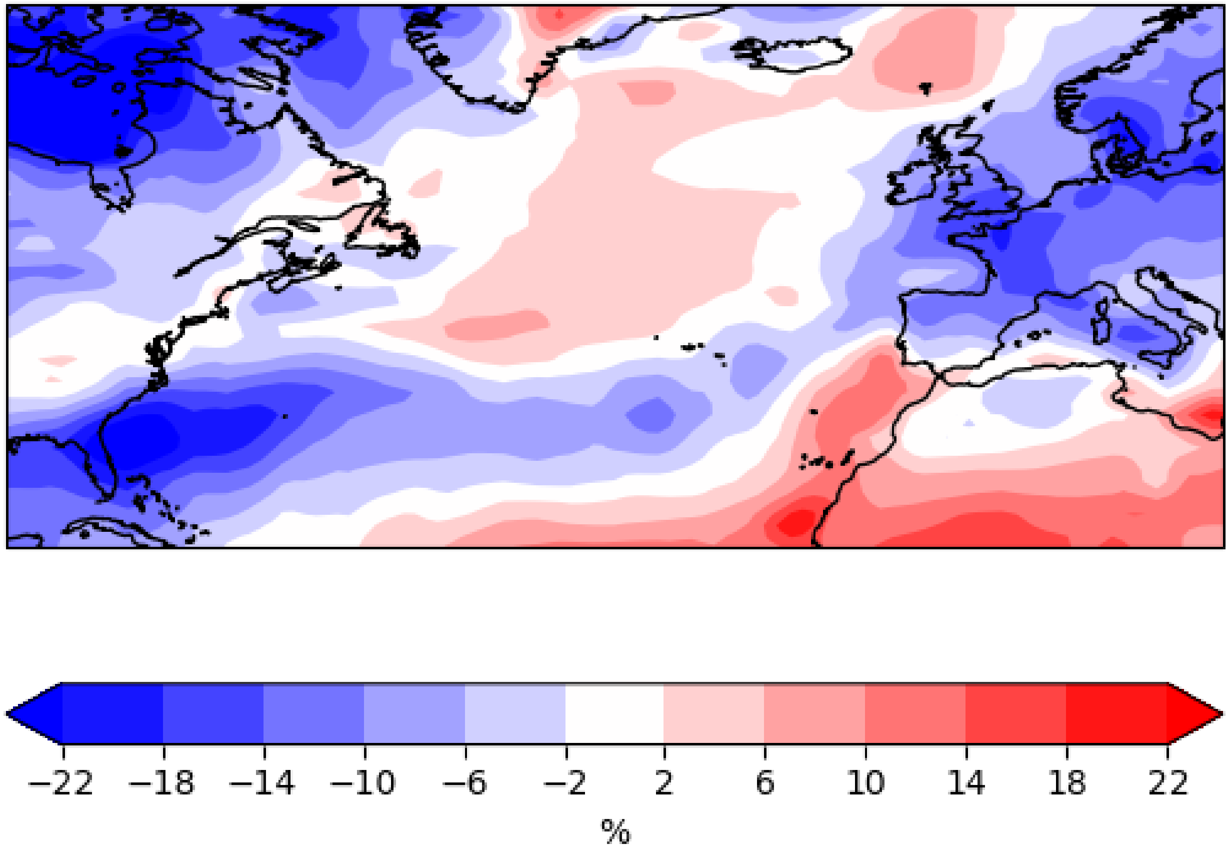

Figure S42: **Mean anomaly (mPWP minus PI) of seasonal cloud fraction over the North Atlantic from models HadCM3, IPSLCM6A and MIROC4m**. Here, negative values (blue) indicate a reduction in cloud cover in the summer during the mPWP, while positive values (red) indicate an increase in cloud cover during the summer in mPWP PlioMIP2 simulations. Note the strong reduction in summer cloud cover over Europe under mPWP conditions.

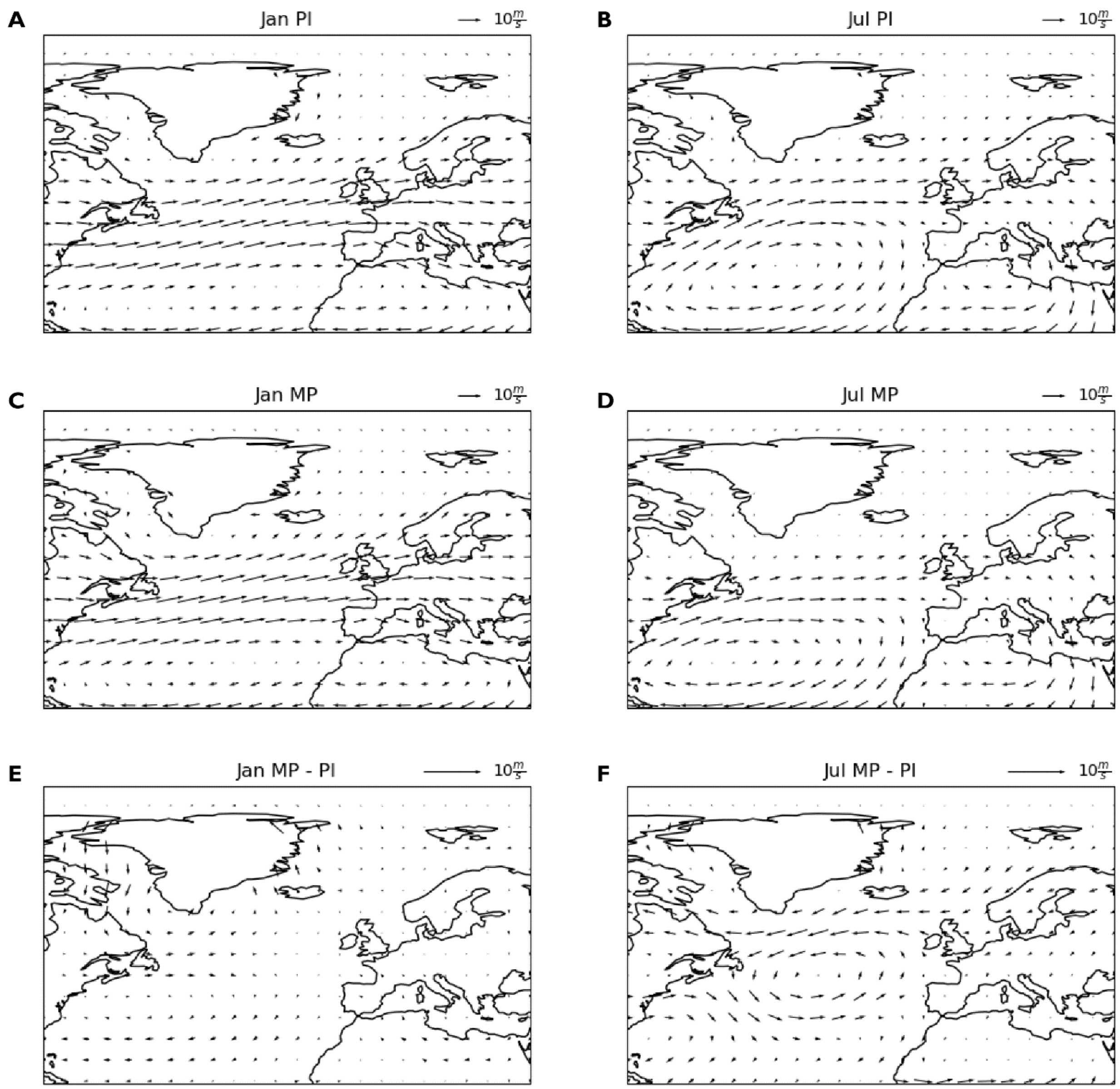

Figure S43: **Multi-model mean outcomes of prevailing wind direction and speed at 850 mb in January (A, C and E) and July (B, D and F) under pre-industrial ("PI"; A & B) and mPWP ("MP"; C & D) conditions and plotted as a difference between mPWP and PI simulations (E & F).** Notice how westerly winds are reduced over Europe in summer under mPWP conditions.

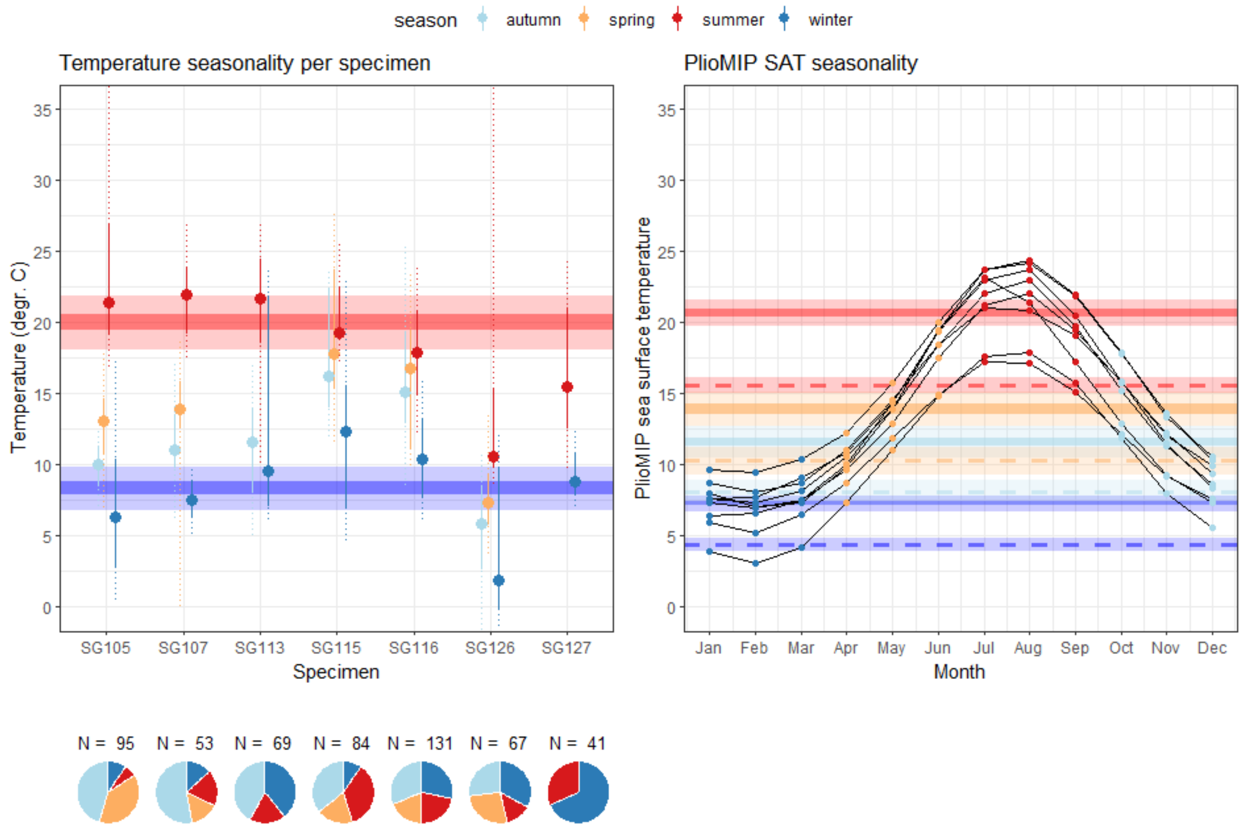

Figure S44: Reconstructed seasonal temperature estimates compared with modelled Surface Air Temperatures. (Top left) Weighted mean seasonal temperatures per specimen (filled circles with solid and dotted vertical lines indicating 68% and 95% confidence levels, respectively) and summer and winter temperatures based on the complete dataset (horizontal shaded bars in red and blue, respectively) with propagated uncertainties (68% and 95% confidence level; see **Methods**). (Top right) Local monthly SAT outcomes of individual PlioMIP2 models with winter (Jan-Mar; blue) and summer (Jul-Sep; red) temperature estimates (horizontal shaded bars showing 68% and 95% CL). Dashed lines in the same colours indicate mean SAT outcomes (with uncertainty; 95% CL(*9*)) from pre-industrial control simulations using the same models. (Bottom left) Clumped isotope sample sizes and seasonal representation of samples per specimen.

5. Modern temperature data

5.1 Local and regional records of sea surface temperature since the Industrial Revolution

Recent (1854-2023) SST data for the southern North Sea basin was obtained from two sources: Firstly, regional averages of SST from the North Sea region (51-55°N, 2-4°E) were extracted from the Extended Reconstructed Sea Surface Temperature product (erSST) curated by the National Oceanic and Atmospheric Administration (NOAA; Washington D.C., USA)(*41*). Secondly, local SST records were obtained from the long-term coastal monitoring station at the Royal Dutch Institute for Sea Research (NIOZ)(*42*). The two different sources were included to allow differentiation between the southern North Sea average SST (including deeper parts of the basin) and very shallow and coastal SST measurements close to the tidal flats of the Wadden Sea. Based on the paleontological and sedimentological work done at the site from which the mPWP fossil shells were obtained, the latter setting is likely to be a closer analogue to the living environment of the animals used for the presented seasonality reconstructions(*35*–*37*).

5.2 Regional SST data from the NOAA erSST dataset

Data from the NOAA erSST v5 dataset were downloaded as 169 individual ASCII files (one per year between 1852 and 2023) containing SST values for each 1° by 1° grid cell at monthly resolution. SST data was aggregated in long format before extracting monthly averages and standard deviations of SST values within the target region of the southern North Sea basin (51-55°N, 2-4°E). During data processing, plots of SST variability within the southern North Sea region were made for each month of each year. These plots are provided in the online supplement ("NOAA_ERSST_monthplots" in "11_process_comparison_data"). Finally, monthly southern North Sea SST values were aggregated into seasonal bins, assigning winter, spring, summer, and autumn seasons using the same criteria as used for PlioMIP2 data (see **section 4.2**). Uncertainties on seasonal SST values from erSST were calculated at the 95% confidence level by propagating the variability within the target region (as measured by the standard deviation of SST values between grid cells) as well as variability between months belonging to the same season. The full data processing procedure is recorded in the online supplement (step 11a). The resulting mean seasonal SST values for each year with their uncertainties were smoothed using a Locally Estimated Scatterplot Smoothing (LOESS) procedure (*123*) with a span of 10% of the length of the record for plotting in **Figure 3** of the main manuscript (**Fig. S45**; see the online supplement step 11b).

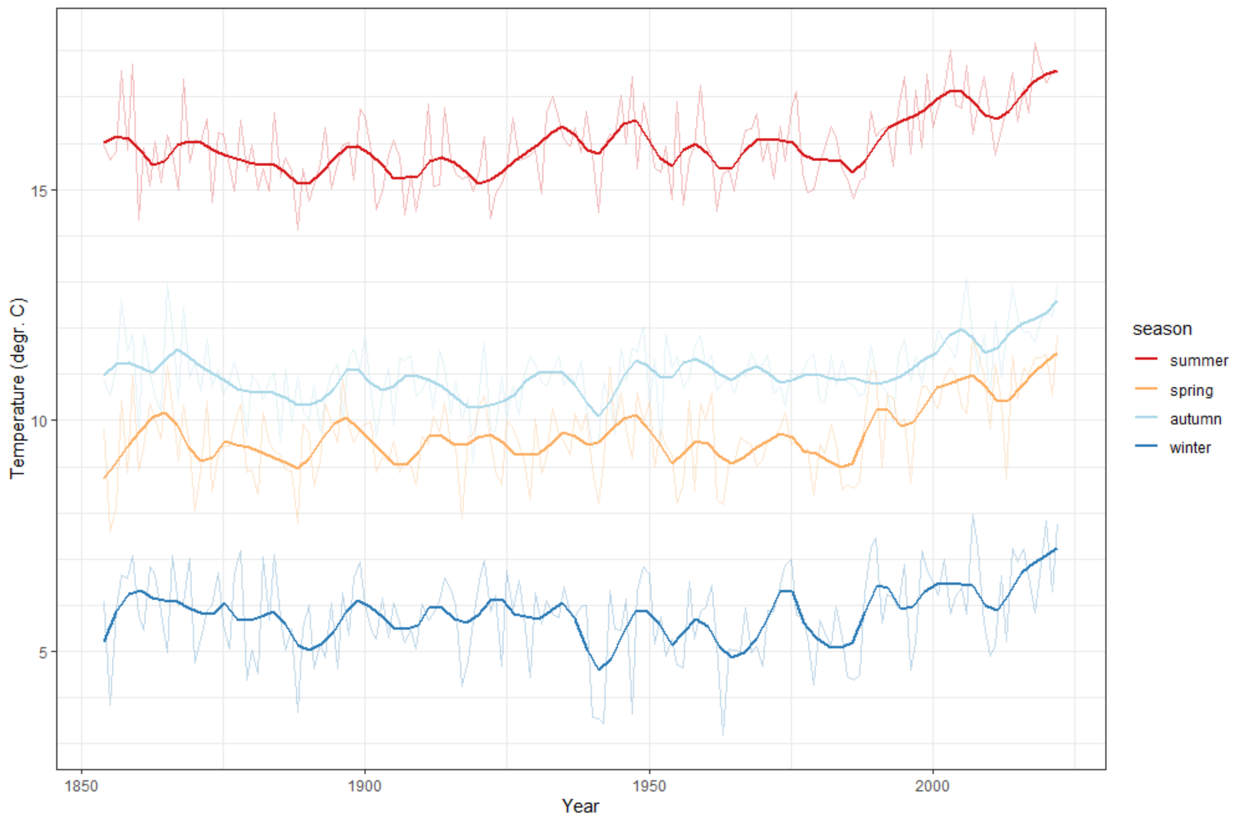

Figure S45: **Seasonal sea surface temperature records from the NOAA ERSST v5 dataset.** Thin lines indicate mean temperatures per season, while thicker lines show loess smoothed temperature records with a smoothing span of 10% of the record.

5.3 Local SST data from the NIOZ jetty dataset

Local SST data were obtained from the Marsdiep tidal inlet south of the island of Texel (NW Netherlands; 53°00'06.5"N 4°47'20.5"E). This inlet serves the Marsdiep basin, with a surface area of ~600 km^2 and an average depth of ~4.5m (*124*). Daily SST measurements have been carried out since 1861 CE in the Marsdiep inlet at 08:00 AM local time. Over this time period, the location and method of these measurements has been modernized, and since 2000 CE SST measurements have been taken by continuous, electronic (10 second resolution) recorders using a temperature sensor placed approximately 1 meter below the low tide water level (total depth: 2 m at low spring tide(*42*)). From this high resolution data the 08:00 AM values (10 minute median) are used to continue the long-term time series, using monthly means to avoid tidal phase bias. Previous studies have reconciled differences in methodology and measurement location over the period 1861 CE until present, making available a continuous monthly SST record of this shallow marine locality(*42*, *125*, *126*). This monthly dataset was provided in the online supplement ("NIOZ_jetty_T_S_dat2.csv" in "North_Sea_records"). The SST data was aggregated into three-monthly time bins, following seasonal definitions in **section 4.2**, and smoothed using a LOESS filter with a span of 10% for plotting (see **section 5.2**). All calculations used to process the local SST data from the NIOZ jetty are provided in the online supplement (step 11a and 11b). Plots of the entire dataset and smoothing results are provided in **Figure. S46**.

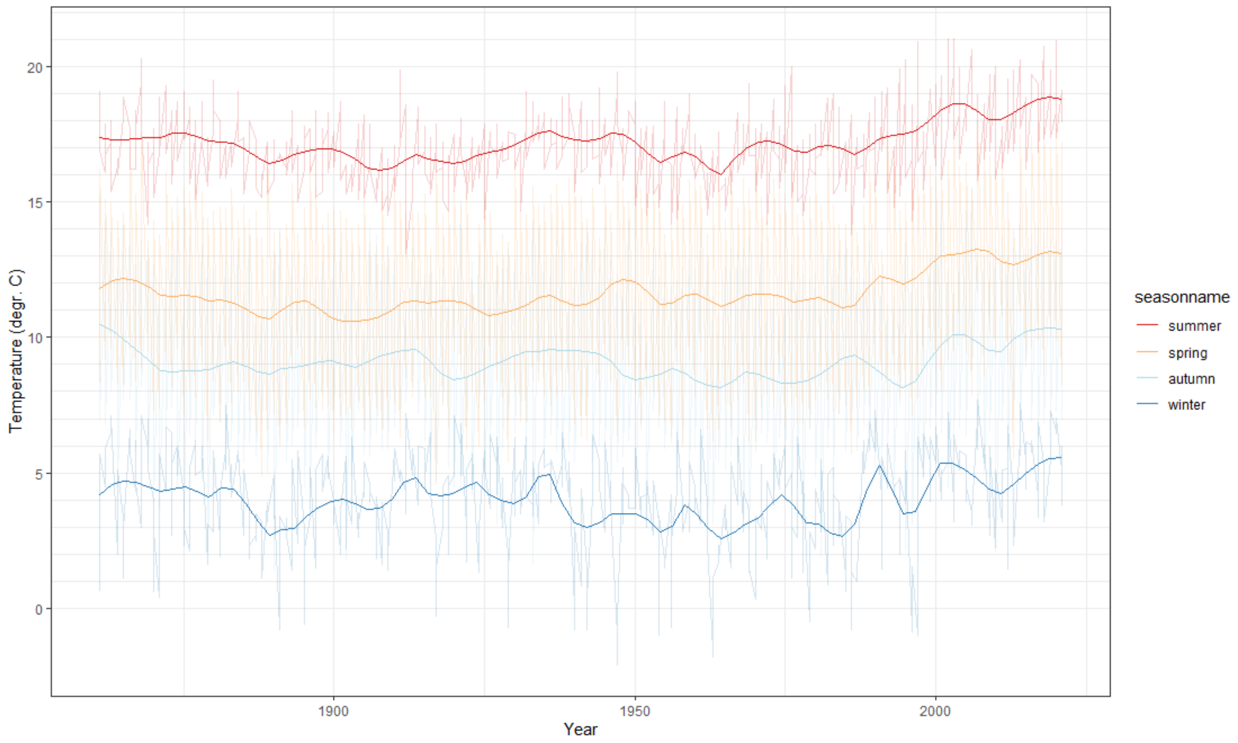

Figure S46: Local seasonal sea surface temperature records from the NIOZ jetty dataset. Thin lines indicate mean temperatures per season, while thicker lines show loess smoothed temperature records with a smoothing span of 10% of the record.

6. CMIP6 model output

Outcomes of SST projections from GCMs in the Coupled Model Intercomparison Project 6 (CMIP6) run using Shared Socioeconomic Pathway (SSP) 2-4.5 were obtained from the Interactive Atlas provided by Working Group I of the Intergovernmental Panel on Climate Change (IPCC(*128*)). Both historical (1850-2014 CE) control runs and climate projections between 2015 and 2100 CE from CMIP6 models were used to create a continuous time series of projected regional temperature data from 1850 CE to 2100 CE. This enabled comparison of the historical model data with observed regional and local SST data (see **section 5**) while at the same time allowing comparison of the projected future climate data under the SSP2-4.5 scenario with the PlioMIP model outcomes and reconstructions for the mPWP. **Table S4** lists the GCM models within the CMIP6 ensemble which provided SST data for the southern North Sea region (51-55°N, 2-4°E) under the SSP2-4.5 scenario and were used in this study. Details of the boundary conditions for the CMIP6 model experiments and the specifics of all 34 models involved in the model intercomparison project can be found in (*48*) and references within. Details of the specifics of the SSP2-4.5 scenario for which these models were run are described in (*4*). Comma delimited data files containing the SST output for all models involved are provided in the online supplement ("CMIP6" in "CMIP_data/IPCC Atlas"). Multi-model mean SSTs based on an average of all CMIP6 models were calculated by aggregating monthly data from each model and then binning the SST data into three-monthly seasonal SST bins, following the seasonal definition as presented in **section 4.2**. The Northern Europe Reference Region, as defined in (*127*) was chosen as a target region for comparison with the other model outcomes and observations in this study. The resulting multi-model mean seasonal SST estimates from the CMIP6 models are provided in

the online supplement (“CMIP6_seasonal_stats.csv” in “11_process_comparison_data”) and calculations used to process CMIP6 data and produce **Figure 3** from this data are provided in the online supplement (step 11a and 11b). An overview of the seasonal SST outcomes of several key time slices from the transient CMIP6 model runs is presented in **Table S5**.

ACCESS-CM2	CNRM-CM6-1	HadGEM3-GC31-LL	MPI-ESM1-2-HR
ACCESS-ESM1-5	CNRM-CM6-1-HR	IITM-ESM	MPI-ESM1-2-LR
AWI-CM-1-1-MR	CNRM-ESM2-1	INM-CM4-8	MRI-ESM2-0
BCC-CSM2-MR	EC-Earth3	INM-CM5-0	NESM3
CAMS-CSM1-0	EC-Earth3-Veg	IPSL-CM6A-LR	NorESM2-LM
CanESM5	EC-Earth3-Veg-LR	KACE-1-0-G	NorESM2-MM
CESM2	FGOALS-g3	KIOST-ESM	UKESM1-0-LL
CESM2-WACCM	GFDL-CM4	MIROC6	
CMCC-CM2-SR5	GFDL-ESM4	MIROC-ES2L	

Table S4: List of GCM models from the CMIP6 ensemble whose SST outcomes were used for both historical and future SSP2-4.5 based SST projections in the southern North Sea.

	Time period	Season	mean Sea Surface Temperature (degr. C)	95% confidence level on mean SST
**Past**	1850-1900	winter	5.5	0.3
		spring	9.3	0.5
		summer	15.7	0.7
		autumn	11.1	0.2
	1961-1990	winter	5.6	0.2
		spring	9.2	0.5
		summer	15.5	0.8
		autumn	11.1	0.2
	1981-2010	winter	6.1	0.2
		spring	9.7	0.6
		summer	16.1	0.8
		autumn	11.7	0.2
	1986-2005	winter	6.1	0.2
		spring	9.7	0.5
		summer	16.1	0.8
		autumn	11.7	0.2
	1995-2014	winter	6.4	0.2
		spring	10.1	0.5
		summer	16.5	0.8
		autumn	12.0	0.2
**Future**	Near Term (2021-2040) SSP2-4.5	winter	6.9	0.3
		spring	11.0	0.4
		summer	17.9	0.5
		autumn	12.7	0.3
	Medium Term (2041-2060) SSP2-4.5	winter	7.2	0.3
		spring	11.3	0.4
		summer	18.3	0.5
		autumn	13.0	0.3
	Long Term (2081-2100) SSP2-4.5	winter	7.7	0.3
		spring	11.9	0.4
		summer	19.1	0.5
		autumn	13.8	0.3

Table S5: Overview of CMIP6 mean winter, spring, summer and autumn SSTs with 95% confidence intervals in the southern North Sea Basin for key time slices in the past and future.

REFERENCES AND NOTES

1. A. M. Haywood, H. J. Dowsett, A. M. Dolan, Integrating geological archives and climate models for the mid-Pliocene warm period. *Nat. Commun.* **7**, 10646 (2016).

2. H. J. Dowsett, K. M. Foley, D. K. Stoll, M. A. Chandler, L. E. Sohl, M. Bentsen, B. L. Otto-Bliesner, F. J. Bragg, W.-L. Chan, C. Contoux, A. M. Dolan, A. M. Haywood, J. A. Jonas, A. Jost, Y. Kamae, G. Lohmann, D. J. Lunt, K. H. Nisancioglu, A. Abe-Ouchi, G. Ramstein, C. R. Riesselman, M. M. Robinson, N. A. Rosenbloom, U. Salzmann, C. Stepanek, S. L. Strother, H. Ueda, Q. Yan, Z. Zhang, Sea surface temperature of the mid-Piacenzian ocean: A data-model comparison. *Sci. Rep.* **3**, 2013 (2013).

3. E. de la Vega, T. B. Chalk, P. A. Wilson, R. P. Bysani, G. L. Foster, Atmospheric CO2 during the Mid-Piacenzian Warm Period and the M2 glaciation. *Sci. Rep.* **10**, 1–8 (2020).

4. IPCC, *Climate Change 2021: The Physical Science Basis. Contribution of Working Group I to the Sixth Assessment Report of the Intergovernmental Panel on Climate Change* (Cambridge Univ. Press, 2021).

5. M. Meinshausen, Z. R. J. Nicholls, J. Lewis, M. J. Gidden, E. Vogel, M. Freund, U. Beyerle, C. Gessner, A. Nauels, N. Bauer, J. G. Canadell, J. S. Daniel, A. John, P. B. Krummel, G. Luderer, N. Meinshausen, S. A. Montzka, P. J. Rayner, S. Reimann, S. J. Smith, M. van den Berg, G. J. M. Velders, M. K. Vollmer, R. H. J. Wang, The shared socio-economic pathway (SSP) greenhouse gas concentrations and their extensions to 2500. *Geosci. Model Dev.* **13**, 3571–3605 (2020).

6. Y. Liang, N. P. Gillett, A. H. Monahan, Climate model projections of 21st century global warming constrained using the observed warming trend. *Geophys. Res. Lett.* **47**, e2019GL086757 (2020).

7. K. D. Burke, J. W. Williams, M. A. Chandler, A. M. Haywood, D. J. Lunt, B. L. Otto-Bliesner, Pliocene and Eocene provide best analogs for near-future climates. *Proc. Natl. Acad. Sci. U.S.A.* **115**, 13288–13293 (2018).

8. Climate Action Tracker, 2100 Warming Projections: Emissions and expected warming based on pledges and current policies, 2022; https://climateactiontracker.org/global/temperatures/.

9. A. M. Haywood, J. C. Tindall, H. J. Dowsett, A. M. Dolan, K. M. Foley, S. J. Hunter, D. J. Hill, W.-L. Chan, A. Abe-Ouchi, C. Stepanek, G. Lohmann, D. Chandan, W. R. Peltier, N. Tan, C. Contoux, G. Ramstein, X. Li, Z. Zhang, C. Guo, K. H. Nisancioglu, Q. Zhang, Q. Li, Y. Kamae, M. A. Chandler, L. E. Sohl, B. L. Otto-Bliesner, R. Feng, E. C. Brady, A. S. Von der Heydt, M. L. J. Baatsen, D. J. Lunt, The Pliocene Model Intercomparison Project Phase 2: Large-scale climate features and climate sensitivity. *Clim. Past* **16**, 2095–2123 (2020).

10. J. Cohen, J. A. Screen, J. C. Furtado, M. Barlow, D. Whittleston, D. Coumou, J. Francis, K. Dethloff, D. Entekhabi, J. Overland, J. Jones, Recent Arctic amplification and extreme mid-latitude weather. *Nat. Geosci.* **7**, 627–637 (2014).

11. D. Coumou, J. Lehmann, J. Beckmann, The weakening summer circulation in the Northern Hemisphere mid-latitudes. *Science* **348**, 324–327 (2015).

12. S. Hofer, A. J. Tedstone, X. Fettweis, J. L. Bamber, Decreasing cloud cover drives the recent mass loss on the Greenland Ice Sheet. *Sci. Adv.* **3**, e1700584 (2017).

13. E. Rousi, F. Selten, S. Rahmstorf, D. Coumou, Changes in North Atlantic atmospheric circulation in a warmer climate favor winter flooding and summer drought over Europe. *J. Clim.* **34**, 2277–2295 (2021).

14. D. Coumou, G. Di Capua, S. Vavrus, L. Wang, S. Wang, The influence of Arctic amplification on mid-latitude summer circulation. *Nat. Commun.* **9**, 2959 (2018).

15. H. J. Dowsett, A. M. Haywood, P. J. Valdes, M. M. Robinson, D. J. Lunt, D. J. Hill, D. K. Stoll, K. M. Foley, Sea surface temperatures of the mid-Piacenzian Warm Period: A comparison of PRISM3 and HadCM3. *Palaeogeogr. Palaeoclimatol. Palaeoecol.* **309**, 83–91 (2011).

16. T. D. Herbert, L. C. Peterson, K. T. Lawrence, Z. Liu, Tropical ocean temperatures over the past 3.5 million years. *Science* **328**, 1530–1534 (2010).

17. K. T. Lawrence, S. Sosdian, H. E. White, Y. Rosenthal, North Atlantic climate evolution through the Plio-Pleistocene climate transitions. *Earth Planet. Sci. Lett.* **300**, 329–342 (2010).

18. J. C. Wit, G.-J. Reichart, S. J. A Jung, D. Kroon, Approaches to unravel seasonality in sea surface temperatures using paired single-specimen foraminiferal δ18O and Mg/Ca analyses. *Paleoceanography* **25**, PA4220 (2010).

19. G. Jia, X. Wang, W. Guo, L. Dong, Seasonal distribution of archaeal lipids in surface water and its constraint on their sources and the TEX86 temperature proxy in sediments of the South China Sea. *J. Geophys. Res. Biogeo.* **122**, 592–606 (2017).

20. M. H. Conte, M.-A. Sicre, C. Rühlemann, J. C. Weber, S. Schulte, D. Schulz-Bull, T. Blanz, Global temperature calibration of the alkenone unsaturation index (UK′37) in surface waters and comparison with surface sediments. *Geochem. Geophys. Geosyst.* **7**, Q02005 (2006).

21. J. C. Tindall, A. M. Haywood, U. Salzmann, A. M. Dolan, T. Fletcher, The warm winter paradox in the Pliocene northern high latitudes. *Clim. Past* **18**, 1385–1405 (2022).

22. D. K. Moss, L. C. Ivany, D. S. Jones, Fossil bivalves and the sclerochronological reawakening. *Paleobiology* **47**, 551–573 (2021).

23. B. R. Schöne, J. Fiebig, Seasonality in the North Sea during the Allerød and Late Medieval Climate Optimum using bivalve sclerochronology. *Int. J. Earth Sci.* **98**, 83–98 (2009).

24. A. L. A. Johnson, A. M. Valentine, B. R. Schöne, M. J. Leng, S. Goolaerts, Sclerochronological evidence of pronounced seasonality from the late Pliocene of the southern North Sea basin and its implications. *Clim. Past* **18**, 1203–1229 (2022).

25. D. J. Reynolds, J. D. Scourse, P. R. Halloran, A. J. Nederbragt, A. D. Wanamaker, P. G. Butler, C. A. Richardson, J. Heinemeier, J. Eiriksson, K. L. Knudsen, Annually resolved North Atlantic marine climate over the last millennium. *Nat. Commun.* **7**, 13502 (2016).

26. D. Bajnai, J. Fiebig, A. Tomašových, S. Milner Garcia, C. Rollion-Bard, J. Raddatz, N. Löffler, C. Primo-Ramos, U. Brand, Assessing kinetic fractionation in brachiopod calcite using clumped isotopes. *Sci. Rep.* **8**, 1–12 (2018).

27. N. J. de Winter, J. Vellekoop, R. Vorsselmans, A. Golreihan, J. Soete, S. V. Petersen, K. W. Meyer, S. Casadio, R. P. Speijer, P. Claeys, An assessment of latest Cretaceous *Pycnodonte vesicularis* (Lamarck, 1806) shells as records for palaeoseasonality: A multi-proxy investigation. *Clim. Past* **14**, 725–749 (2018).

28. L. C. Ivany, Reconstructing paleoseasonality from accretionary skeletal carbonates—Challenges and opportunities. *Paleontol. Soc. Papers* **18**, 133–166 (2012).

29. D. A. Smale, T. Wernberg, E. C. J. Oliver, M. Thomsen, B. P. Harvey, S. C. Straub, M. T. Burrows, L. V. Alexander, J. A. Benthuysen, M. G. Donat, M. Feng, A. J. Hobday, N. J. Holbrook, S. E. Perkins-Kirkpatrick, H. A. Scannell, A. Sen Gupta, B. L. Payne, P. J. Moore, Marine heatwaves threaten global biodiversity and the provision of ecosystem services. *Nat. Clim. Change* **9**, 306–312 (2019).

30. J. Garrabou, D. Gómez-Gras, A. Medrano, C. Cerrano, M. Ponti, R. Schlegel, N. Bensoussan, E. Turicchia, M. Sini, V. Gerovasileiou, N. Teixido, A. Mirasole, L. Tamburello, E. Cebrian, G. Rilov, J.-B. Ledoux, J. B. Souissi, F. Khamassi, R. Ghanem, M. Benabdi, S. Grimes, O. Ocaña, H. Bazairi, B. Hereu, C. Linares, D. K. Kersting, G. la Rovira, J. Ortega, D. Casals, M. Pagès-Escolà, N. Margarit, P. Capdevila, J. Verdura, A. Ramos, A. Izquierdo, C. Barbera, E. Rubio-Portillo, I. Anton, P. López-Sendino, D. Díaz, M. Vázquez-Luis, C. Duarte, N. Marbà, E. Aspillaga, F. Espinosa, D. Grech, I. Guala, E. Azzurro, S. Farina, M. Cristina Gambi, G. Chimienti, M. Montefalcone, A. Azzola, T. P. Mantas, S. Fraschetti, G. Ceccherelli, S. Kipson, T. Bakran-Petricioli, D. Petricioli, C. Jimenez, S. Katsanevakis, I. T. Kizilkaya, Z. Kizilkaya, S. Sartoretto, R. Elodie, S. Ruitton, S. Comeau, J.-P. Gattuso, J.-G. Harmelin, Marine heatwaves drive recurrent mass mortalities in the Mediterranean Sea. *Glob. Change Biol.* **28**, 5708–5725 (2022).

31. S. Manes, M. J. Costello, H. Beckett, A. Debnath, E. Devenish-Nelson, K.-A. Grey, R. Jenkins, T. M. Khan, W. Kiessling, C. Krause, S. S. Maharaj, G. F. Midgley, J. Price, G. Talukdar, M. M. Vale, Endemism increases species' climate change risk in areas of global biodiversity importance. *Biol. Conserv.* **257**, 109070 (2021).

32. A. Toimil, I. J. Losada, R. J. Nicholls, R. A. Dalrymple, M. J. F. Stive, Addressing the challenges of climate change risks and adaptation in coastal areas: A review. *Coas. Eng.* **156**, 103611 (2020).

33. D. Huyghe, M. Daëron, M. de Rafelis, D. Blamart, M. Sébilo, Y.-M. Paulet, F. Lartaud, Clumped isotopes in modern marine bivalves. *Geochim. Cosmochim. Acta* **316**, 41–58 (2022).

34. N. J. de Winter, R. Witbaard, I. J. Kocken, I. A. Müller, J. Guo, B. Goudsmit, M. Ziegler, Temperature dependence of clumped isotopes (Δ47) in aragonite. *Geophys. Res. Lett.* **49**, e2022GL099479 (2022).

35. F. P. Wesselingh, F. S. Busschers, S. Goolaerts, Observations on the Pliocene sediments exposed at Antwerp International Airport (northern Belgium) constrain the stratigraphic position of the Broechem fauna. *Geol. Belgica* **23**, 315–321 (2020).

36. R. Marquet, Ecology and evolution of Pliocene bivalves from the Antwerp Basin. *Bull. Inst. R. Sci. Nat. Belg. Sci. Terre* **74**, 205–212 (2004).

37. N. Vandenberghe, S. Louwye, An introduction to the Neogene stratigraphy of northern Belgium: Present status. *Geol. Belgica* **23**, 97–112 (2020).

38. M. Quante, F. Colijn, *North Sea Region Climate Change Assessment* (Springer Nature, 2016).

39. J. Deckers, S. Louwye, S. Goolaerts, The internal division of the Pliocene Lillo Formation: Correlation between Cone Penetration Tests and lithostratigraphic type sections. *Geol. Belgica* **23**, 333–343 (2020).

40. A. M. Haywood, H. J. Dowsett, A. M. Dolan, D. Rowley, A. Abe-Ouchi, B. Otto-Bliesner, M. A. Chandler, S. J. Hunter, D. J. Lunt, M. Pound, U. Salzmann, The Pliocene Model Intercomparison Project (PlioMIP) Phase 2: Scientific objectives and experimental design. *Clim. Past* **12**, 663–675 (2016).

41. B. Huang, P. W. Thorne, V. F. Banzon, T. Boyer, G. Chepurin, J. H. Lawrimore, M. J. Menne, T. M. Smith, R. S. Vose, H.-M. Zhang, Extended reconstructed sea surface temperature, version 5 (ERSSTv5): Upgrades, validations, and intercomparisons. *J. Clim.* **30**, 8179–8205 (2017).

42. H. M. van Aken, Variability of the water temperature in the western Wadden Sea on tidal to centennial time scales. *J. Sea Res.* **60**, 227–234 (2008).

43. W. de Nooijer, Q. Zhang, Q. Li, Q. Zhang, X. Li, Z. Zhang, C. Guo, K. H. Nisancioglu, A. M. Haywood, J. C. Tindall, S. J. Hunter, H. J. Dowsett, C. Stepanek, G. Lohmann, B. L. Otto-Bliesner, R.

Feng, L. E. Sohl, M. A. Chandler, N. Tan, C. Contoux, G. Ramstein, M. L. J. Baatsen, A. S. von der Heydt, D. Chandan, W. R. Peltier, A. Abe-Ouchi, W.-L. Chan, Y. Kamae, C. M. Brierley, Evaluation of Arctic warming in mid-Pliocene climate simulations. *Clim. Past* **16**, 2325–2341 (2020).

44. Z. Song, M. Latif, W. Park, Y. Zhang, Influence of model bias on simulating North Atlantic sea surface temperature during the mid-Pliocene. *Paleocean. Paleoclimatol.* **33**, 884–893 (2018).

45. F. Cook, R. O. Smith, M. Roughan, N. J. Cullen, N. Shears, M. Bowen, Marine heatwaves in shallow coastal ecosystems are coupled with the atmosphere: Insights from half a century of daily in situ temperature records. *Front. Climate* **4**, 1012022 (2022).

46. J. Sharples, O. N. Ross, B. E. Scott, S. P. R. Greenstreet, H. Fraser, Inter-annual variability in the timing of stratification and the spring bloom in the North-western North Sea. *Cont. Shelf Res.* **26**, 733–751 (2006).

47. A. Valentine, A. L. A. Johnson, M. J. Leng, H. J. Sloane, P. S. Balson, Isotopic evidence of cool winter conditions in the mid-Piacenzian (Pliocene) of the southern North Sea Basin. *Palaeogeogr. Palaeoclimatol. Palaeoecol.* **309**, 9–16 (2011).

48. B. C. O'Neill, C. Tebaldi, D. P. van Vuuren, V. Eyring, P. Friedlingstein, G. Hurtt, R. Knutti, E. Kriegler, J.-F. Lamarque, J. Lowe, G. A. Meehl, R. Moss, K. Riahi, B. M. Sanderson, The Scenario Model Intercomparison Project (ScenarioMIP) for CMIP6. *Geosci. Model Dev.* **9**, 3461–3482 (2016).

49. J. E. Tierney, C. J. Poulsen, I. P. Montañez, T. Bhattacharya, R. Feng, H. L. Ford, B. Hönisch, G. N. Inglis, S. V. Petersen, N. Sagoo, C. R. Tabor, K. Thirumalai, J. Zhu, N. J. Burls, G. L. Foster, Y. Goddéris, B. T. Huber, L. C. Ivany, S. K. Turner, D. J. Lunt, J. C. McElwain, B. J. W. Mills, B. L. Otto-Bliesner, A. Ridgwell, Y. G. Zhang, Past climates inform our future. *Science* **370**, eaay3701 (2020).

50. M. P. Byrne, P. A. O'Gorman, Trends in continental temperature and humidity directly linked to ocean warming. *Proc. Natl. Acad. Sci. U.S.A.* **115**, 4863–4868 (2018).

51. C. Clotten, R. Stein, K. Fahl, S. De Schepper, Seasonal sea ice cover during the warm Pliocene: Evidence from the Iceland Sea (ODP Site 907). *Earth Planet. Sci. Lett.* **481**, 61–72 (2018).

52. J. Knies, P. Cabedo-Sanz, S. T. Belt, S. Baranwal, S. Fietz, A. Rosell-Melé, The emergence of modern sea ice cover in the Arctic Ocean. *Nat. Commun.* **5**, 5608 (2014).

53. T. W. N. Haine, T. Martin, The Arctic-Subarctic sea ice system is entering a seasonal regime: Implications for future Arctic amplification. *Sci. Rep.* **7**, 4618 (2017).

54. A. Dai, D. Luo, M. Song, J. Liu, Arctic amplification is caused by sea-ice loss under increasing CO2. *Nat. Commun.* **10**, 121 (2019).

55. J. Kyselý, R. Huth, Changes in atmospheric circulation over Europe detected by objective and subjective methods. *Theor. Appl. Climatol.* **85**, 19–36 (2006).

56. J. Kyselý, Influence of the persistence of circulation patterns on warm and cold temperature anomalies in Europe: Analysis over the 20th century. *Global Planet. Change* **62**, 147–163 (2008).

57. P. A. Stott, N. Christidis, F. E. L. Otto, Y. Sun, J.-P. Vanderlinden, G. J. van Oldenborgh, R. Vautard, H. von Storch, P. Walton, P. Yiou, F. W. Zwiers, Attribution of extreme weather and climate-related events. *WIREs Clim. Change* **7**, 23–41 (2016).

58. B. Schuldt, A. Buras, M. Arend, Y. Vitasse, C. Beierkuhnlein, A. Damm, M. Gharun, T. E. E. Grams, M. Hauck, P. Hajek, H. Hartmann, E. Hiltbrunner, G. Hoch, M. Holloway-Phillips, C. Körner, E. Larysch, T. Lübbe, D. B. Nelson, A. Rammig, A. Rigling, L. Rose, N. K. Ruehr, K. Schumann, F. Weiser, C. Werner, T. Wohlgemuth, C. S. Zang, A. Kahmen, A first assessment of the impact of the extreme 2018 summer drought on Central European forests. *Basic Appl. Ecol.* **45**, 86–103 (2020).

59. E. Steirou, L. Gerlitz, H. Apel, X. Sun, B. Merz, Climate influences on flood probabilities across Europe. *Hydrol. Earth Syst. Sci.* **23**, 1305–1322 (2019).

60. N. A. Kamenos, North Atlantic summers have warmed more than winters since 1353, and the response of marine zooplankton. *Proc. Natl. Acad. Sci. U.S.A.* **107**, 22442–22447 (2010).

61. C. Schär, P. L. Vidale, D. Lüthi, C. Frei, C. Häberli, M. A. Liniger, C. Appenzeller, The role of increasing temperature variability in European summer heatwaves. *Nature* **427**, 332–336 (2004).

62. T. L. Frölicher, E. M. Fischer, N. Gruber, Marine heatwaves under global warming. *Nature* **560**, 360–364 (2018).

63. J. F. Piatt, J. K. Parrish, H. M. Renner, S. K. Schoen, T. T. Jones, M. L. Arimitsu, K. J. Kuletz, B. Bodenstein, M. García-Reyes, R. S. Duerr, R. M. Corcoran, R. S. A. Kaler, G. J. McChesney, R. T. Golightly, H. A. Coletti, R. M. Suryan, H. K. Burgess, J. Lindsey, K. Lindquist, P. M. Warzybok, J. Jahncke, J. Roletto, W. J. Sydeman, Extreme mortality and reproductive failure of common murres resulting from the northeast Pacific marine heatwave of 2014-2016. *PLOS ONE* **15**, e0226087 (2020).

64. T. P. Hughes, J. T. Kerry, M. Álvarez-Noriega, J. G. Álvarez-Romero, K. D. Anderson, A. H. Baird, R. C. Babcock, M. Beger, D. R. Bellwood, R. Berkelmans, T. C. Bridge, I. R. Butler, M. Byrne, N. E. Cantin, S. Comeau, S. R. Connolly, G. S. Cumming, S. J. Dalton, G. Diaz-Pulido, C. M. Eakin, W. F. Figueira, J. P. Gilmour, H. B. Harrison, S. F. Heron, A. S. Hoey, J.-P. A. Hobbs, M. O. Hoogenboom, E. V. Kennedy, C. Kuo, J. M. Lough, R. J. Lowe, G. Liu, M. T. McCulloch, H. A. Malcolm, M. J. McWilliam, J. M. Pandolfi, R. J. Pears, M. S. Pratchett, V. Schoepf, T. Simpson, W. J. Skirving, B. Sommer, G. Torda, D. R. Wachenfeld, B. L. Willis, S. K. Wilson, Global warming and recurrent mass bleaching of corals. *Nature* **543**, 373–377 (2017).

65. Scripps Institution of Oceanography, L. Cavole, A. Demko, R. Diner, A. Giddings, I. Koester, C. Pagniello, M.-L. Paulsen, A. Ramirez-Valdez, S. Schwenck, N. Yen, M. Zill, P. Franks, Biological impacts of the 2013–2015 warm-water anomaly in the Northeast Pacific: Winners, losers, and the future. *Oceanography* **29**, 273–285 (2016).

66. H.-O. Pörtner, D. C. Roberts, M. M. B. Tignor, E. S. Poloczanska, K. Mintenbeck, A. Alegría, M. Craig, S. Langsdorf, S. Löschke, V. Möller, A. Okem, B. Rama, Eds., *Climate Change 2022: Impacts, Adaptation and Vulnerability. Contribution of Working Group II to the Sixth Assessment Report of the Intergovernmental Panel on Climate Change* (Cambridge Univ. Press, 2022).

67. N. M. A. Wichern, N. J. de Winter, A. L. A. Johnson, S. Goolaerts, F. Wesselingh, M. F. Hamers, P. Kaskes, P. Claeys, M. Ziegler, The fossil bivalve *Angulus benedeni benedeni*: A potential seasonally resolved stable-isotope-based climate archive to investigate Pliocene temperatures in the southern North Sea basin. *Biogeosciences* **20**, 2317–2345 (2022).

68. B. R. Schöne, J. Fiebig, M. Pfeiffer, R. Gleβ, J. Hickson, A. L. Johnson, W. Dreyer, W. Oschmann, Climate records from a bivalved Methuselah (Arctica islandica, Mollusca; Iceland). *Palaeogeogr. Palaeoclimatol. Palaeoecol.* **228**, 130–148 (2005).

69. A. N. Meckler, M. Ziegler, M. I. Millán, S. F. Breitenbach, S. M. Bernasconi, Long-term performance of the Kiel carbonate device with a new correction scheme for clumped isotope measurements. *Rapid Commun. Mass Spectrom.* **28**, 1705–1715 (2014).

70. I. A. Müller, A. Fernandez, J. Radke, J. van Dijk, D. Bowen, J. Schwieters, S. M. Bernasconi, Carbonate clumped isotope analyses with the long-integration dual-inlet (LIDI) workflow: Scratching at the lower sample weight boundaries. *Rapid Commun. Mass Spectrom.* **31**, 1057–1066 (2017).

71. S. T. Murray, M. M. Arienzo, P. K. Swart, Determining the Δ47 acid fractionation in dolomites. *Geochim. Cosmochim. Acta* **174**, 42–53 (2016).

72. S. M. Bernasconi, I. A. Müller, K. D. Bergmann, S. F. Breitenbach, A. Fernandez, D. A. Hodell, M. Jaggi, A. N. Meckler, I. Millan, M. Ziegler, Reducing uncertainties in carbonate clumped isotope analysis through consistent carbonate-based standardization. *Geochem. Geophys. Geosyst.* **19**, 2895–2914 (2018).

73. I. J. Kocken, I. A. Müller, M. Ziegler, Optimizing the use of carbonate standards to minimize uncertainties in clumped isotope data. *Geochem. Geophys. Geosyst.* **20**, 5565–5577 (2019).

74. W. A. Brand, S. S. Assonov, T. B. Coplen, Correction for the 17O interference in δ(13C) measurements when analyzing CO2 with stable isotope mass spectrometry (IUPAC Technical Report). *Pure Appl. Chem.* **82**, 1719–1733 (2010).

75. N. J. de Winter, ShellChron 0.2.8: A new tool for constructing chronologies in accretionary carbonate archives from stable oxygen isotope profiles. *Geosci. Model Dev.*, 1–37 (2021).

76. N. J. de Winter, T. Agterhuis, M. Ziegler, Optimizing sampling strategies in high-resolution paleoclimate records. *Clim. Past* **17**, 1315–1340 (2021).

77. N. Meinicke, M. A. Reimi, A. C. Ravelo, A. N. Meckler, Coupled Mg/Ca and clumped isotope measurements indicate lack of substantial mixed layer cooling in the Western Pacific Warm Pool During the last ~5 million years. *Paleoceanog. Paleoclimatol.* **36**, e2020Pa004115 (2021).

78. K. W. Huntington, J. M. Eiler, H. P. Affek, W. Guo, M. Bonifacie, L. Y. Yeung, N. Thiagarajan, B. Passey, A. Tripati, M. Daëron, Methods and limitations of 'clumped' CO2 isotope (Δ47) analysis by gas-source isotope ratio mass spectrometry. *J. Mass Spectrom.* **44**, 1318–1329 (2009).

79. M. Iturbide, J. Fernández, J. M. Gutiérrez, A. Pirani, D. Huard, A. Al Khourdajie, J. Baño-Medina, J. Bedia, A. Casanueva, E. Cimadevilla, A. S. Cofiño, M. De Felice, J. Diez-Sierra, M. García-Díez, J. Goldie, D. A. Herrera, S. Herrera, R. Manzanas, J. Milovac, A. Radhakrishnan, D. San-Martín, A. Spinuso, K. M. Thyng, C. Trenham, Ö. Yelekçi, Implementation of FAIR principles in the IPCC: The WGI AR6 Atlas repository. *Sci. Data* **9**, 629 (2022).

80. R Core Team, *R: A Language and Environment for Statistical Computing* (R Foundation for Statistical Computing, 2023); https://R-project.org/.

81. J. Schindelin, I. Arganda-Carreras, E. Frise, V. Kaynig, M. Longair, T. Pietzsch, S. Preibisch, C. Rueden, S. Saalfeld, B. Schmid, Fiji: An open-source platform for biological-image analysis. *Nat. Methods* **9**, 676–682 (2012).

82. H. Dowsett, A. Dolan, D. Rowley, R. Moucha, A. M. Forte, J. X. Mitrovica, M. Pound, U. Salzmann, M. Robinson, M. Chandler, K. Foley, A. Haywood, The PRISM4 (mid-Piacenzian) paleoenvironmental reconstruction. *Clim. Past* **12**, 1519–1538 (2016).

83. L. E. Lisiecki, M. E. Raymo, A Pliocene-Pleistocene stack of 57 globally distributed benthic δ18O records. *Paleoceanography* **20**, PA1003 (2005).

84. J. K. Cochran, K. Kallenberg, N. H. Landman, P. J. Harries, D. Weinreb, K. K. Turekian, A. J. Beck, W. A. Cobban, Effect of diagenesis on the Sr, O, and C isotope composition of late Cretaceous mollusks from the Western Interior Seaway of North America. *Am. J. Sci.* **310**, 69–88 (2010).

85. N. J. de Winter, J. Vellekoop, A. J. Clark, P. Stassen, R. P. Speijer, P. Claeys, The giant marine gastropod Campanile giganteum (Lamarck, 1804) as a high-resolution archive of seasonality in the Eocene greenhouse world. *Geochem. Geophys. Geosyst.* **21**, e2019GC008794 (2020).

86. N. Höche, M. Peharda, E. O. Walliser, B. R. Schöne, Morphological variations of crossed-lamellar ultrastructures of Glycymeris bimaculata (Bivalvia) serve as a marine temperature proxy. *Estuarine Coastal Shelf Sci.* **237**, 106658 (2020).

87. G. Crippa, E. Griesshaber, A. G. Checa, E. M. Harper, M. S. Roda, W. W. Schmahl, Orientation patterns of aragonitic crossed-lamellar, fibrous prismatic and myostracal microstructures of modern Glycymeris shells. *J. Struct. Biol.* **212**, 107653 (2020).

88. K. Janiszewska, M. Mazur, M. Machalski, J. Stolarski, From pristine aragonite to blocky calcite: Exceptional preservation and diagenesis of cephalopod nacre in porous Cretaceous limestones. *PLOS ONE* **13**, e0208598 (2018).

89. V. Barbin, K. Ramseyer, J. P. Debenay, E. Schein, M. Roux, D. Decrouez, Cathodoluminescence of recent biogenic carbonates: Environmental and ontogenetic fingerprint. *Geol. Mag.* **128**, 19–26 (1991).

90. V. Barbin, Cathodoluminescence of carbonate shells: Biochemical vs diagenetic process, in *Cathodoluminescence in Geosciences* (Springer, 2000), pp. 303–329.

91. U. Brand, J. Veizer, Chemical diagenesis of a multicomponent carbonate system–1: Trace elements. *J. Sediment. Res.* **50**, 1219–1236 (1980).

92. N. J. de Winter, P. Claeys, Micro X-ray fluorescence (μXRF) line scanning on Cretaceous rudist bivalves: A new method for reproducible trace element profiles in bivalve calcite. *Sedimentology* **64**, 231–251 (2017).

93. M. Pagel, V. Barbin, P. Blanc, D. Ohnenstetter, *Cathodoluminescence in Geosciences* (Springer Science & Business Media, 2013).

94. T. Götte, D. K. Richter, Quantitative aspects of Mn-activated cathodoluminescence of natural and synthetic aragonite. *Sedimentology* **56**, 483–492 (2009).

95. M. Cusack, Biomineral electron backscatter diffraction for palaeontology. *Palaeontology* **59**, 171–179 (2016).

96. L. A. Casella, E. Griesshaber, X. Yin, A. Ziegler, V. Mavromatis, D. Müller, A.-C. Ritter, D. Hippler, E. M. Harper, M. Dietzel, A. Immenhauser, B. R. Schöne, L. Angiolini, W. W. Schmahl, Experimental diagenesis: Insights into aragonite to calcite transformation of *Arctica islandica* shells by hydrothermal treatment. *Biogeosciences* **14**, 1461–1492 (2017).

97. A.-C. Ritter, V. Mavromatis, M. Dietzel, O. Kwiecien, F. Wiethoff, E. Griesshaber, L. A. Casella, W. W. Schmahl, J. Koelen, R. D. Neuser, A. Leis, D. Buhl, A. Niedermayr, S. F. M. Breitenbach, S. M. Bernasconi, A. Immenhauser, Exploring the impact of diagenesis on (isotope) geochemical and microstructural alteration features in biogenic aragonite. *Sedimentology* **64**, 1354–1380 (2017).

98. M. C. Marcano, T. D. Frank, S. B. Mukasa, K. C. Lohmann, M. Taviani, Diagenetic incorporation of Sr into aragonitic bivalve shells: Implications for chronostratigraphic and palaeoenvironmental interpretations. *Depos. Rec.* **1**, 38–52 (2015).

99. S. V. Popov, Formation of bivalve shells and their microstructure. *Paleontol. J.* **48**, 1519–1531 (2014).

100. A. G. Checa, F. J. Esteban-Delgado, A. B. Rodríguez-Navarro, Crystallographic structure of the foliated calcite of bivalves. *J. Struct. Biol.* **157**, 393–402 (2007).

101. P. S. Freitas, L. J. Clarke, H. Kennedy, C. A. Richardson, Ion microprobe assessment of the heterogeneity of Mg/Ca, Sr/Ca and Mn/Ca ratios in Pecten maximus and Mytilus edulis (bivalvia) shell calcite precipitated at constant temperature. *Biogeosciences* **6**, 1209–1227 (2009).

102. N. de Winter, M. Sinnesael, C. Makarona, S. Vansteenberge, P. Claeys, Trace element analyses of carbonates using portable and micro-X-ray fluorescence: Performance and optimization of measurement parameters and strategies. *J. Anal. At. Spectrom.* **32**, 1211–1223 (2017).

103. J. Vellekoop, P. Kaskes, M. Sinnesael, J. Huygh, T. Déhais, J. W. M. Jagt, R. P. Speijer, P. Claeys, A new age model and chemostratigraphic framework for the Maastrichtian type area (southeastern Netherlands, northeastern Belgium). *Newslett. Stratigr.* **55**, 479–501 (2022).

104. I. S. Al-Aasm, J. Veizer, Diagenetic stabilization of aragonite and low-mg calcite, I. Trace elements in rudists. *J. Sediment. Res.* **56**, 763–770 (1986).

105. J. P. Hendry, P. W. Ditchfield, J. D. Marshall, Two-stage neomorphism of Jurassic aragonitic bivalves: Implications for early diagenesis. *J. Sediment. Res.* **65**, 214–224 (1995).

106. M. Carré, I. Bentaleb, O. Bruguier, E. Ordinola, N. T. Barrett, M. Fontugne, Calcification rate influence on trace element concentrations in aragonitic bivalve shells: Evidences and mechanisms. *Geochim. Cosmochim. Acta* **70**, 4906–4920 (2006).

107. B. Lafuente, R. T. Downs, H. Yang, N. Stone, 1. The power of databases: The RRUFF project, in *Highlights in Mineralogical Crystallography* (De Gruyter (O), 2015), pp. 1–30.

108. P. Ghosh, J. Adkins, H. Affek, B. Balta, W. Guo, E. A. Schauble, D. Schrag, J. M. Eiler, 13C–18O bonds in carbonate minerals: A new kind of paleothermometer. *Geochim. Cosmochim. Acta* **70**, 1439–1456 (2006).

109. M. Daëron, D. Blamart, M. Peral, H. P. Affek, Absolute isotopic abundance ratios and the accuracy of $\Delta 47$ measurements. *Chem. Geol.* **442**, 83–96 (2016).

110. IAEA-C-2, Nucleus; https://nucleus.iaea.org/sites/ReferenceMaterials.

111. I. A. Müller, J. D. Rodriguez-Blanco, J.-C. Storck, G. S. D. Nascimento, T. R. R. Bontognali, C. Vasconcelos, L. G. Benning, S. M. Bernasconi, Calibration of the oxygen and clumped isotope thermometers for (proto-)dolomite based on synthetic and natural carbonates. *Chem. Geol.* **525**, 1–17 (2019).

112. S. M. Bernasconi, M. Daëron, K. D. Bergmann, M. Bonifacie, A. N. Meckler, H. P. Affek, N. Anderson, D. Bajnai, E. Barkan, E. Beverly, D. Blamart, L. Burgener, D. Calmels, C. Chaduteau, M. Clog, B. Davidheiser-Kroll, A. Davies, F. Dux, J. Eiler, B. Elliott, A. C. Fetrow, J. Fiebig, S. Goldberg, M. Hermoso, K. W. Huntington, E. Hyland, M. Ingalls, M. Jaggi, C. M. John, A. B. Jost, S. Katz, J. Kelson, T. Kluge, I. J. Kocken, A. Laskar, T. J. Leutert, D. Liang, J. Lucarelli, T. J. Mackey, X. Mangenot, N. Meinicke, S. E. Modestou, I. A. Müller, S. Murray, A. Neary, N. Packard, B. H. Passey, E. Pelletier, S. Petersen, A. Piasecki, A. Schauer, K. E. Snell, P. K. Swart, A. Tripati, D. Upadhyay, T.

Vennemann, I. Winkelstern, D. Yarian, N. Yoshida, N. Zhang, M. Ziegler, InterCarb: A community effort to improve interlaboratory standardization of the carbonate clumped isotope thermometer using carbonate standards. *Geochem. Geophys. Geosyst.* **22**, e2020GC009588 (2021).

113. N. de Winter, T. Agterhuis, M. Ziegler, Optimizing sampling strategies in high-resolution paleoclimate records. *Clim. Past Discuss.* **17**, 1315–1340 (2020).

114. D. Chandan, W. R. Peltier, Regional and global climate for the mid-Pliocene using the University of Toronto version of CCSM4 and PlioMIP2 boundary conditions. *Clim. Past* **13**, 919–942 (2017).

115. M. L. J. Baatsen, A. S. von der Heydt, M. A. Kliphuis, A. M. Oldeman, J. E. Weiffenbach, Warm mid-Pliocene conditions without high climate sensitivity: The CCSM4-Utrecht (CESM 1.0.5) contribution to the PlioMIP2. *Clim. Past* **18**, 657–679 (2022).

116. C. Stepanek, E. Samakinwa, G. Knorr, G. Lohmann, Contribution of the coupled atmosphere–ocean–sea ice–vegetation model COSMOS to the PlioMIP2. *Clim. Past* **16**, 2275–2323 (2020).

117. J. Zheng, Q. Zhang, Q. Li, Q. Zhang, M. Cai, Contribution of sea ice albedo and insulation effects to Arctic amplification in the EC-Earth Pliocene simulation. *Clim. Past* **15**, 291–305 (2019).

118. M. Kelley, G. A. Schmidt, L. S. Nazarenko, S. E. Bauer, R. Ruedy, G. L. Russell, A. S. Ackerman, I. Aleinov, M. Bauer, R. Bleck, V. Canuto, G. Cesana, Y. Cheng, T. L. Clune, B. I. Cook, C. A. Cruz, A. D. Del Genio, G. S. Elsaesser, G. Faluvegi, N. Y. Kiang, D. Kim, A. A. Lacis, A. Leboissetier, A. N. LeGrande, K. K. Lo, J. Marshall, E. E. Matthews, S. McDermid, K. Mezuman, R. L. Miller, L. T. Murray, V. Oinas, C. Orbe, C. P. García-Pando, J. P. Perlwitz, M. J. Puma, D. Rind, A. Romanou, D. T. Shindell, S. Sun, N. Tausnev, K. Tsigaridis, G. Tselioudis, E. Weng, J. Wu, M.-S. Yao, GISS-E2.1: Configurations and climatology. *J. Adv. Model. Earth Syst.* **12**, e2019MS002025 (2020).

119. S. J. Hunter, A. M. Haywood, A. M. Dolan, J. C. Tindall, The HadCM3 contribution to PlioMIP phase 2. *Clim. Past* **15**, 1691–1713 (2019).

120. J.-L. Dufresne, M.-A. Foujols, S. Denvil, A. Caubel, O. Marti, O. Aumont, Y. Balkanski, S. Bekki, H. Bellenger, R. Benshila, S. Bony, L. Bopp, P. Braconnot, P. Brockmann, P. Cadule, F. Cheruy, F. Codron, A. Cozic, D. Cugnet, N. de Noblet, J.-P. Duvel, C. Ethé, L. Fairhead, T. Fichefet, S. Flavoni, P.

Friedlingstein, J.-Y. Grandpeix, L. Guez, E. Guilyardi, D. Hauglustaine, F. Hourdin, A. Idelkadi, J. Ghattas, S. Joussaume, M. Kageyama, G. Krinner, S. Labetoulle, A. Lahellec, M.-P. Lefebvre, F. Lefevre, C. Levy, Z. X. Li, J. Lloyd, F. Lott, G. Madec, M. Mancip, M. Marchand, S. Masson, Y. Meurdesoif, J. Mignot, I. Musat, S. Parouty, J. Polcher, C. Rio, M. Schulz, D. Swingedouw, S. Szopa, C. Talandier, P. Terray, N. Viovy, N. Vuichard, Climate change projections using the IPSL-CM5 Earth System Model: From CMIP3 to CMIP5. *Clim. Dyn.* **40**, 2123–2165 (2013).

121. W.-L. Chan, A. Abe-Ouchi, Pliocene Model Intercomparison Project (PlioMIP2) simulations using the Model for Interdisciplinary Research on Climate (MIROC4m). *Clim. Past* **16**, 1523–1545 (2020).

122. X. Li, C. Guo, Z. Zhang, O. H. Otterå, R. Zhang, PlioMIP2 simulations with NorESM-L and NorESM1-F. *Clim. Past* **16**, 183–197 (2020).

123. W. S. Cleveland, E. Grosse, W. M. Shyu, Local regression models, in *Statistical Models in S* (Wadsworth & Brooks/Cole, 1992).

124. H. Ridderinkhof, Tidal and residual flows in the western Dutch Wadden Sea II: An analytical model to study the constant flow between connected tidal basins. *Netherl. J. Sea Res.* **22**, 185–198 (1988).

125. H. M. Van Aken, 140 years of daily observations in a tidal inlet (Marsdiep). *ICES Mar. Sci. Symp.* **219**, 359–361 (2003).

126. P. C. T. Van der Hoeven, Observations of surface water temperature and salinity, State Office of Fishery Research (RIVO): 1860–1981. *KNMI Sci. Rep. WR* **82**, 2 (1982).

127. IPCC AR6-WGI Atlas, https://interactive-atlas.ipcc.ch/atlas.

128. IPCC, *Climate Change 2013: The Physical Science Basis: Working Group I Contribution to the Fifth Assessment Report of the Intergovernmental Panel on Climate Change* (Cambridge Univ. Press, 2013).
